# Quantum Chemical and Experimental Studies of an Unprecedented Reaction Pathway of Nucleophilic Substitution of 2-Bromomethyl-1,3-thiaselenole with 1,3-Benzothiazole-2-thiol Proceeding Stepwise at Three Different Centers of Seleniranium Intermediates

**DOI:** 10.3390/molecules26216685

**Published:** 2021-11-04

**Authors:** Svetlana V. Amosova, Vladimir A. Shagun, Nataliya A. Makhaeva, Irina A. Novokshonova, Vladimir A. Potapov

**Affiliations:** Siberian Division of the Russian Academy of Sciences, A.E. Favorsky Irkutsk Institute of Chemistry, Favorsky Str. 1, 664033 Irkutsk, Russia; shagun@irioch.irk.ru (V.A.S.); ggm@irioch.irk.ru (N.A.M.); Lotelen@yandex.ru (I.A.N.); v.a.potapov@mail.ru (V.A.P.)

**Keywords:** 2-bromomethyl-1,3-thiaselenole, 1,3-benzothiazole-2-thiol, vinyl sulfides, 2,3-dihydro-1,4-thiaselenine, seleniranium cation, rearrangement

## Abstract

The results of quantum chemical and experimental studies of the reaction of 2-bromomethyl-1,3-thiaselenole with 1,3-benzothiazole-2-thiol made it possible to discover the unprecedented pathway of this reaction, which proceeds stepwise at three different centers of seleniranium intermediates. The first stage includes an attack of thiolate anion at the selenium atom of the seleniranium cation accompanied by ring opening with the formation of (Z)-2-[(1,3-benzothiazol-2-ylsulfanyl)selanyl]ethenyl vinyl sulfide, which is converted to six-membered heterocycle, 2-(2,3-dihydro-1,4-thiaselenin-2-ylsulfanyl)-1,3-benzothiazole, in a 99% yield. The latter compound undergoes rearrangement with ring contraction producing five-membered heterocycle, 2-[(1,3-thiaselenol-2-ylmethyl)sulfanyl]-1,3-benzothiazole, in a 99% yield (the thermodynamic product). The formation of 1,2-bis[(*Z*)-2-(vinylsulfanyl)ethenyl] diselenide is the result of the disproportionation of (Z)-2-[(1,3-benzothiazol-2-ylsulfanyl)selanyl]ethenyl vinyl sulfide. Thus, based on the quantum chemical and experimental studies, a regioselective synthesis of the reaction products in high yields was developed.

## 1. Introduction

**Figure 1:**
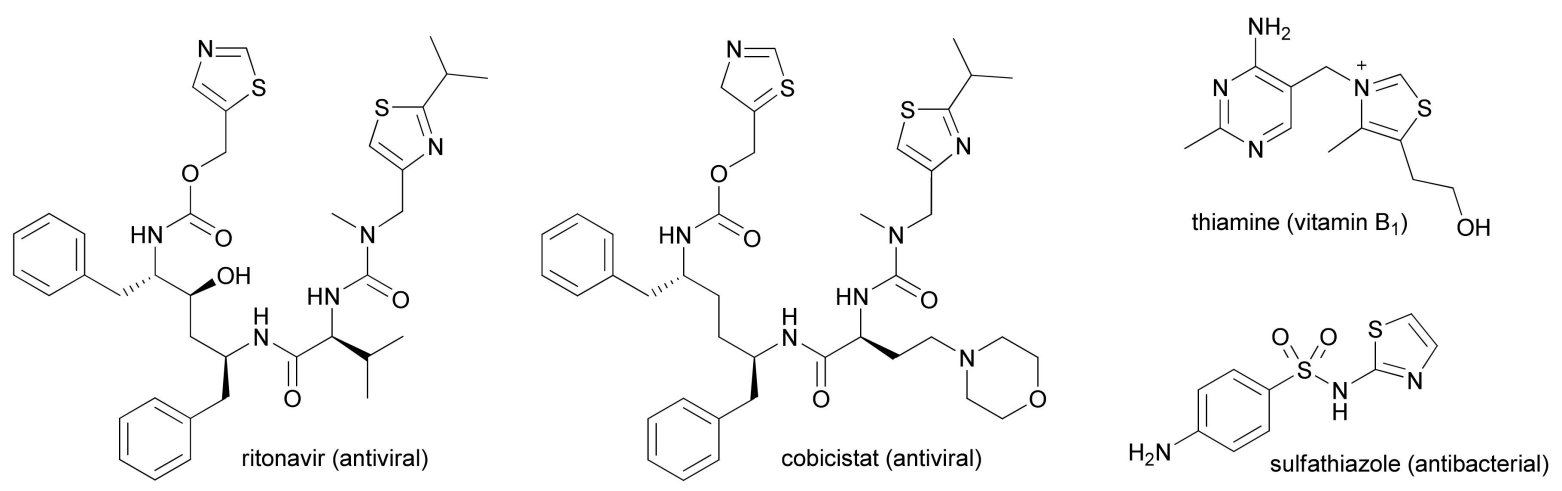
Natural and synthetic drugs containing thiazole ring.

More than 80% of modern drugs contain heterocyclic fragments in their structures [1]. Nitrogen heterocycles are among the most significant structural components of pharmaceuticals. Analysis of the database of U.S. FDA-approved drugs reveals that 59% of unique small-molecule drugs contain a nitrogen heterocycle [1]. The 1,3-thiazole ring is one of most important scaffolds for medicinal chemistry and many 1,3-thiazole derivatives exhibit high biological activity. Thiazole ranks sixth among the 27 classes of the most used nitrogen-containing heterocycles [2]. The thiazole ring is a structural component of more then 30 modern drugs including very significant anti-HIV drugs such as ritonavir and cobicistat, each of which contains two thiazole heterocycles with different functional environments [2]. Besides, the thiazole cycle is a chemically active center of the very important coenzyme thiamine (vitamin B1), antibacterial sulfathiazole, and many other natural and synthetic drugs (Figure 1) [3,4]. Compounds containing a condensed thiazole ring, 2,3-dihydro[1,3]thiazolo[3,2-*a*]pyridines and [1,3]thiazolo[3,2-*a*]quinolines, show high antibacterial [5,6,7,8] and antitumor activity [9,10,11,12]. Platinum group metal complexes containing a benzothiazole moiety exhibit antibacterial [13] and antimicrobial [14] activity.

Sulfur continues to maintain its status as the dominating heteroatom, integrated into a set of 362 sulfur-containing FDA-approved drugs [15]. Application of organoselenium compounds is one of modern trends in bioorganic chemistry. Organoselenium compounds show wide spectrum of biological activities [16,17,18,19,20,21,22,23,24,25,26,27,28,29,30,31]. Functionalized selenides containing benzenesulfonamide moieties were found to inhibit five human isoforms of zinc enzyme carbonic anhydrase. These enzymes are implicated in a variety of diseases, including glaucoma, retinitis pigmentosa, epilepsy, arthritis, and tumors [32]. The chemistry of selenium-containing five- and six-membered heterocycles is rapidly developing, and these compounds show various types of biological activities [33,34,35,36,37].

Selenium-containing electrophilic reagents play one of the main roles in the development of chemo-, regio-, and stereoselective methods for the synthesis of functionalized organoselenium compounds by selenofunctionalization reactions [38,39,40,41,42,43,44,45]. We were the first to use selenium dichloride and dibromide for the synthesis of organoselenium compounds [46,47], and these electrophilic reagents sufficiently expanded the possibilities of selenofunctionalization reactions, demonstrating high efficiency and selectivity [48,49,50,51,52,53].

The anchimeric effect of the selenium atom was quantitatively evaluated by estimation of the rates of nucleophilic substitution reactions in 2,6-dichloro-9-selena[3.3.1]bicyclononane, which was obtained by the addution of selenium dichloride to 1,5-cyclooctadiene [54].

A novel methodology for the synthesis of condensed selenium heterocycles based on the annulation and annulation–methoxylation reactions of selenium dihalides with natural compounds was developed [55]. The complexes of selenium dihalides with 2,6-dihalo-9-selenabicyclo[3.3.1]nonanes, which are the first representatives of coordination compounds with the Se–Se–Se bond, were obtained [56].

The starting compound, 2-bromomethyl-1,3-thiaselenole, was obtained with high selectivity by one-pot synthesis based on the reaction of divinyl sulfide with selenium dibromide [57]. 2-Bromomethyl-1,3-thiaselenole is a unique reagent demonstrating unusual reactivity in nucleophilic substitution reactions as a result of high anchimeric assistance of selenium [54].

**Scheme 1:**
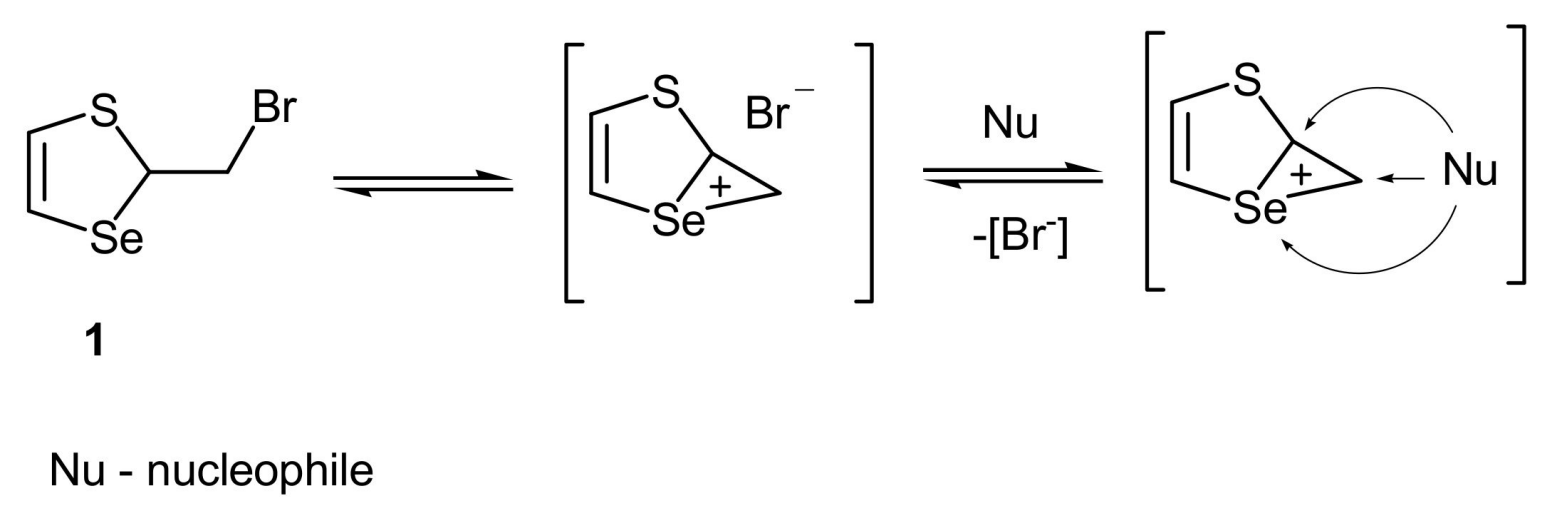
The generation of intermediate seleniranium cation in nucleophilic substitution reactions of thiaselenole **1**, and three possible directions of the reaction by nucleophilic attack at electrophilic centers of the seleniranium cation.

2-Bromomethyl-1,3-thiaselenole (**1**) in solutions exists in equilibrium with intermediate seleniranium cation, which determines its unusual chemical properties [57,58,59,60,61] (Scheme 1). The formation of intermediate seleniranium cation in nucleophilic substitution reactions of thiaselenole **1** with dithiocarbamates was supported by quantum chemical calculations [59]. Nucleophilic attack on seleniranium cation can occur at three electrophilic centers of the seleniranium cation (two carbon atoms and the selenium atom) determining three possible directions of the reaction (Scheme 1).

## 2. Results and Discussion

The present work is a necessary continuation of pioneering studies of the new methodology of nucleophilic substitution at three different centers of seleniranium intermediates in reactions of 2-bromomethyl-1,3-thiaselenole (**1**) with nucleophilic reagents (Scheme 1) [57,58,59,60,61]. The analysis of the data includes a comparison with the previously obtained results on the reactions of 2-bromomethyl-1,3-thiaselenole with sulfur-centered nucleophiles [57,58,59,60]. The reactions of thiaselenole **1** with sulfur-centered nucleophiles with the formation of compounds with the sulfur–selenium bond are of great scientific interest since they include the nucleophilic attack of thiols or thiolate anions at the selenium atom of seleniranium cations. It should be emphasized that the reactions of nucleophilic substitution at the selenium atom of seleniranium cations were unknown before our investigations. However, compounds with the sulfur–selenium bond often play the role of intermediates, which underwent conversion into thermodynamically more stable products.

**Scheme 2:**
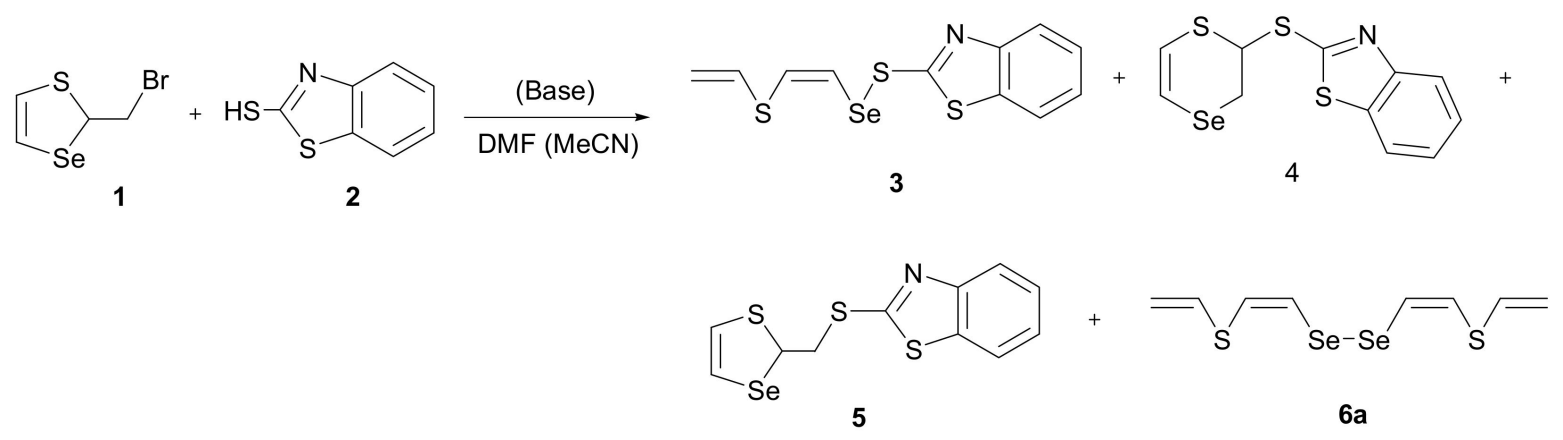
Products of the reaction of thiaselenole **1** with 1,3-benzimidazole-2-thiol **2**.

Depending on the conditions, the reaction of thiaselenole **1** with 1,3-benzothiazole-2-thiol (**2**) leads to the formation of products **3**–**6** (Scheme 2): (*Z*)-2-[(1,3-benzothiazol-2-ylsulfanyl)selanyl]ethenyl vinyl sulfide (**3**), 2-(2,3-dihydro-1,4-thiaselenin-2-ylsulfanyl)-1,3-benzothiazole (**4**), 2-[(1,3-thiaselenol-2-ylmethyl)sulfanyl]-1,3-benzothiazole (**5**), and 1,2-bis[(*Z*)-2-(vinylsulfanyl)ethenyl] diselenide (**6a**).

**Scheme 3:**
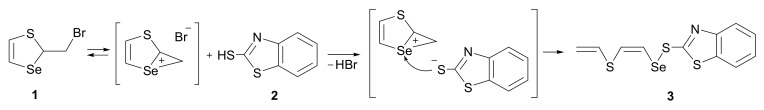
A pathway of the reaction of thiaselenole **1** with thiol **2** with the formation of (Z)-2-[(1,3-benzothiazol-2-ylsulfanyl)selanyl]ethenyl vinyl sulfide (**3**).

The formation of compound **3** with the sulfur–selenium bond is the result of a nucleophilic attack of thiol or thiolate anion at the selenium atom of seleniranium cation (Scheme 3).

We found the reaction of thiaselenole **1** with benzothiazole-2-thiol **2** very promising for advanced experimental and theoretical studies and carried out quantum chemical calculations and optimization of the reaction conditions. The obtained results are discussed in the present article.

**Scheme 4:**
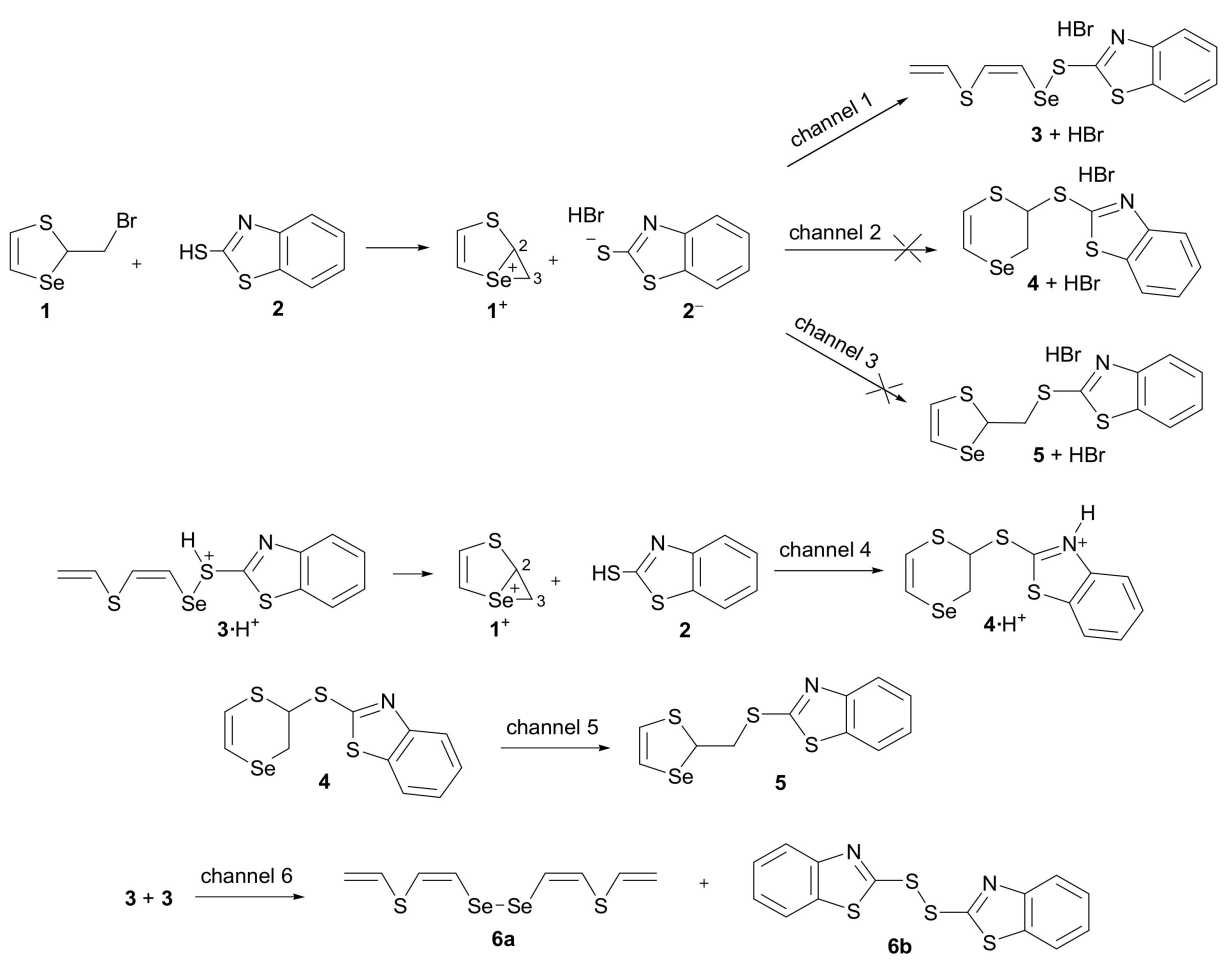
Quantum chemical calculations and analysis of thermodynamic characteristics of multichannel interaction of thiaselenole **1** with thiol **2**.

**Figure 2:**
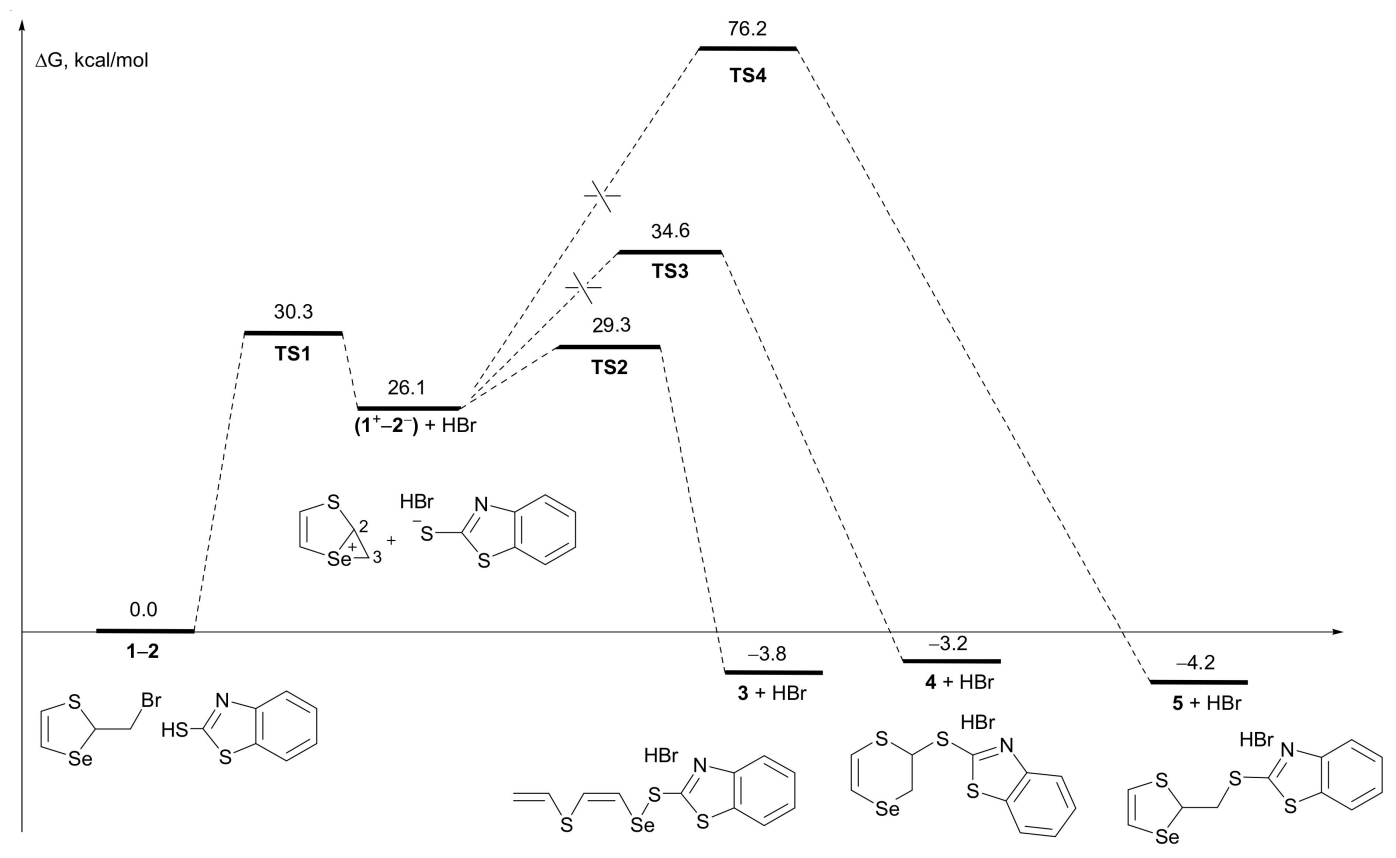
Schematic representations of processes of the formation of selanyl sulfide **3**, six-membered product **4**, and five-membered heterocycle **5** according to B3LYP/6-311^+^G(d,p).

Quantum chemical calculations and analysis of the thermodynamic characteristics of the multichannel interaction of thiaselenole **1** with thiol **2** were carried out (Scheme 4). The first stage of reaction channels **1**–**3** leads to the formation of the ion pair (**1^+^**−**2^−^**). This process proceeds via the **TS1** transition state overcoming a 30.3 kcal/mol barrier (Figure 2). A cleavage of the C–Br bond in thiaselenole **1** leads to the formation of a new C–Se bond and seleniranium cation (**1^+^**). Hydrogen bromide, which released in the reaction, forms a donor–acceptor bond (2.127 Å) with 1,3-benzothiazole-2-thiolate anion (**2^−^**). As a result, the molecular system (**1^+^**−**2^−^**) + HBr is stabilized in a potential well, the depth of which from the side of the reverse transition (**1^+^**−**2^−^**) + HBr → (**1**−**2**) is 4.2 kcal/mol, and the total thermal effect of endothermic rearrangement [(**1**−**2**) → (**1^+^**−**2^−^**) + HBr] is 26.1 kcal/mol (Figure 2).

Based on these studies (Scheme 4, Figure 2), two important conclusions can be drawn: the reaction proceeds via the formation of seleniranium cation and the channel with a nucleophilic attack of the thiolate anion **2^−^** at the selenium atom of the intermediate seleniranium cation is kinetically preferable, with the formation of selanyl sulfide **3** [(**1^+^**−**2^−^**) + HBr → **3** + HBr] (Scheme 4, channel 1). Channel 1 is energetically superior compared to the channels leading to the formation of 2-(2,3-dihydro-1,4-thiaselenin-2-ylsulfanyl)-1,3-benzothiazole (**4**) and 2-[(1,3-thiaselenol-2-ylmethyl)sulfanyl]-1,3-benzothiazole (**5**) by nucleophilic attacks at the carbon atoms C-2 and C-3 of the intermediate seleniranium cation (Scheme 4, channels 2 and 3). The ring opening of thiaselenole **1** proceeds via the **TS2** transition state (Figure 2, channel 1) overcoming the 3.2 kcal/mol barrier. The heat effect of the reaction is 29.9 kcal/mol. The two channels, 4 and 5, or [(**1^+^**−**2^−^**) + HBr → **4** + HBr] and [(**1^+^**−**2^−^**) + HBr → **5** + HBr], respectively, are unable to compete with channel 1 (Figure 2), and the main product of the reaction of thiaselenole **1** with thiol **2** is linear selanyl sulfide **3**.

**Figure 3:**
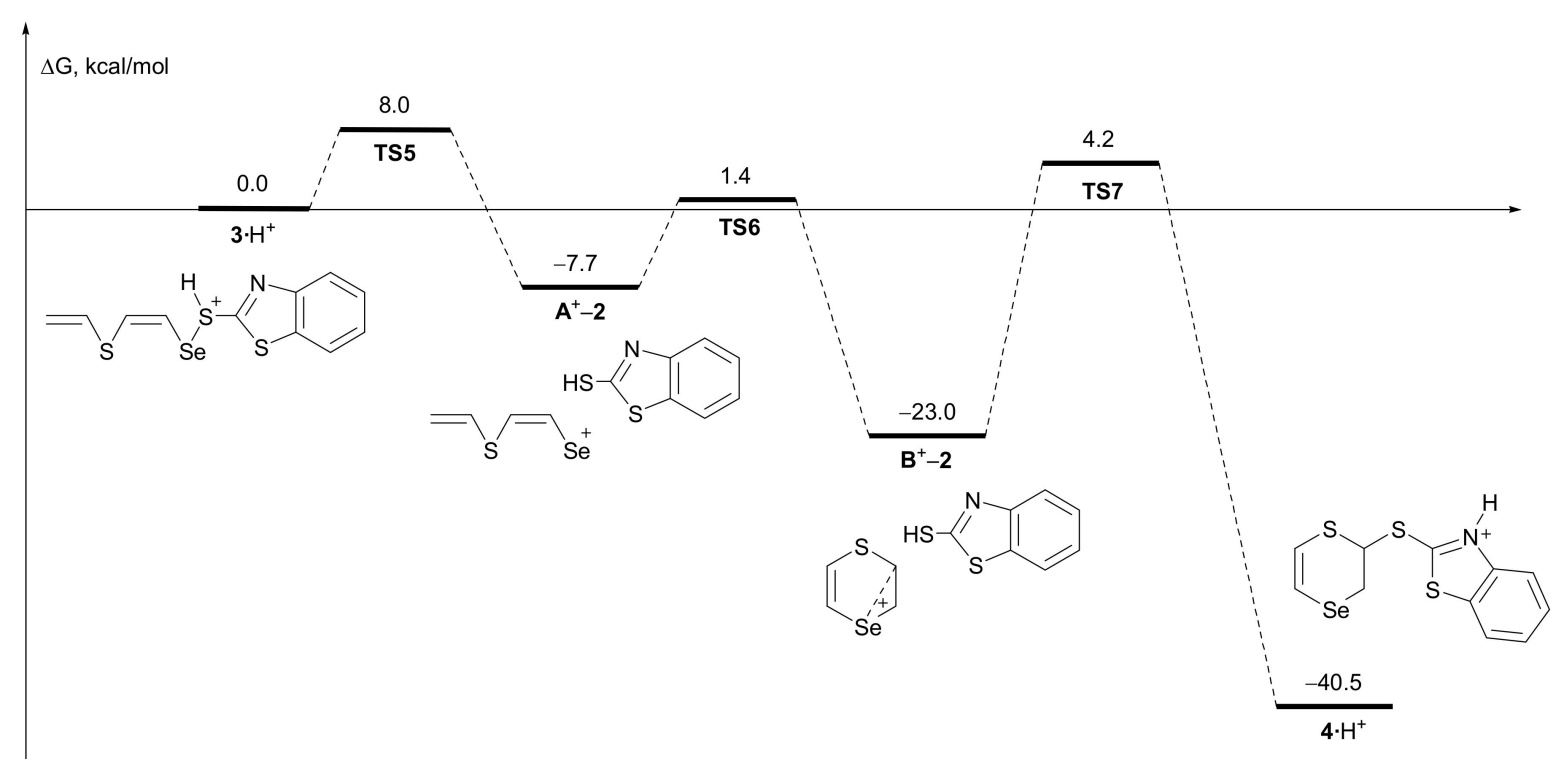
Schematic representation of the process of intramolecular rearrangement **3·**H^+^ → **4·**H^+^ according to B3LYP/6-311+G(d,p).

The obtained data allowed us to direct our investigations towards the search for new reaction conditions in order to increase the yield of selanyl sulfide **3** (varying temperature, the nature of a solvent, the presence of a base, the nature of a base, and concentration of reagents under homogeneous or heterogeneous conditions). An alternative pathway for the formation of six-membered thiaselenine heterocycle **4** from selanyl sulfide **3** by acid-catalyzed cyclization (Figure 3) with the participation of hydrogen bromide, which is released with the formation of selanyl sulfide **3**, was considered. Previously, we studied acid-catalyzed cyclization of a wide range of functionalized selanyl sulfides affording 2,3-dihydro-1,4-thiaselenines in up to 96% yields [60]. It was shown that the cyclization is catalyzed by a number of acids (HClO_4_, CF_3_COOH, and AcOH).

**Figure 4:**
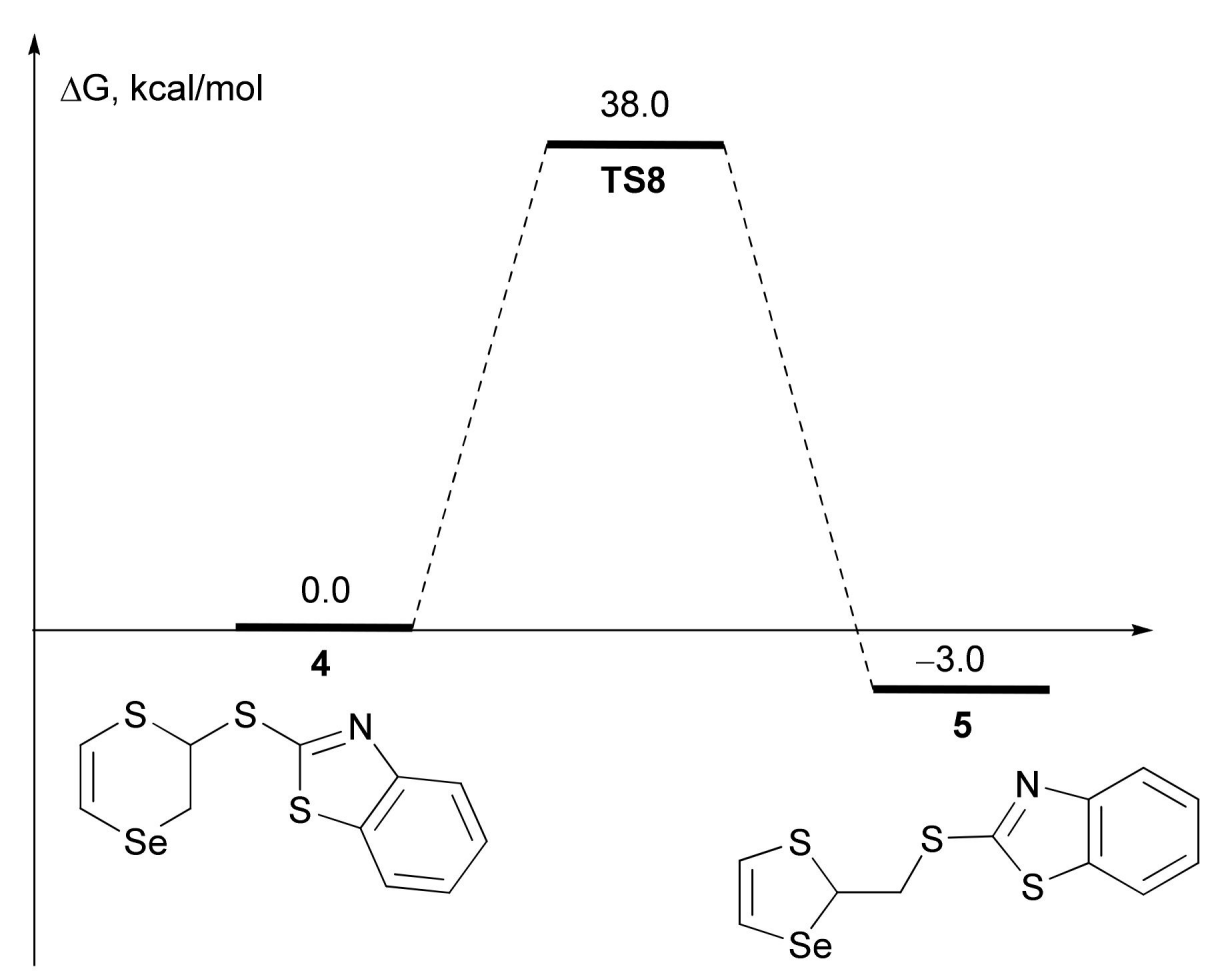
Schematic representation of the process of intramolecular rearrangement **4** → **5** according to B3LYP/6-311^+^G(d,p).

It has been experimentally proven by ^1^H−NMR monitoring that the reaction of thiaselenole **1** with thiol **2** is accompanied by rearrangement of **4** into **5** (acetonitrile, Li_2_CO_3_, r.t.), which proceeds via the intermediate seleniranium cation (Figure 4) [58]. Similarly, the reaction thiaselenole **1** with potassium selenocyanate proceeds via a rearrangement with ring expansion leading to six-membered 2,3-dihydro-1,4-thiaselenin-2-yl selenocyanate (the kinetic product), which in turn undergoes rearrangement with ring contraction into 1,3-thiaselenol-2-ylmethyl selenocyanate (the thermodynamic product) [61].

**Figure 5:**
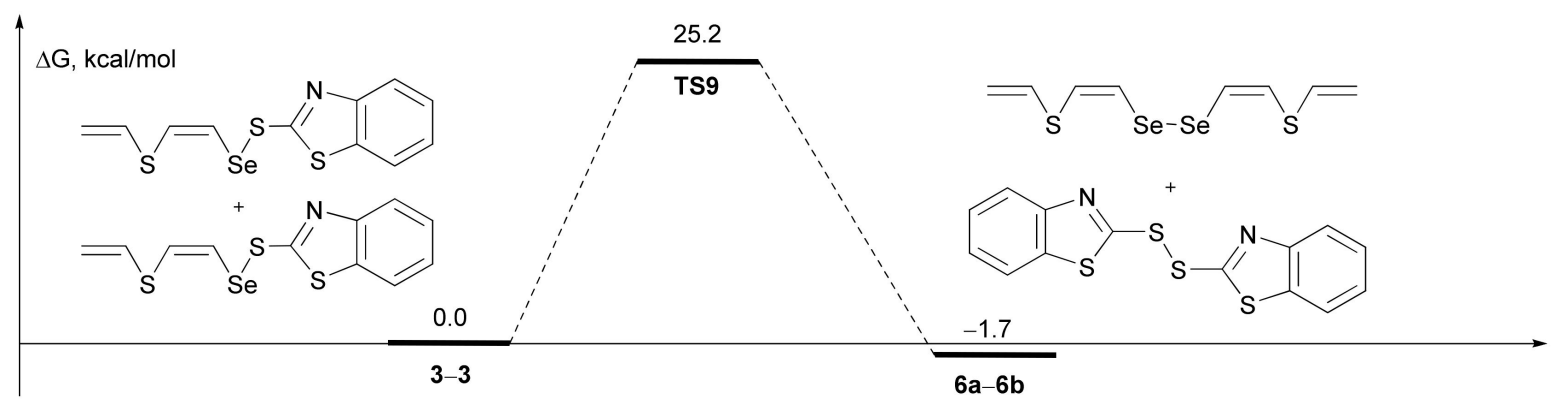
Schematic representation of the disproportionation reaction **3**–**3** → **6a**–**6b**.

The reaction of disproportionation of selanyl sulfide **3** with the formation of two symmetrical products, 1,2-bis[(*Z*)-2-(vinylsulfanyl)ethenyl] diselenide (**6a**) and bis(1,3-benzothiazol-2-yl) disulfide (**6b**), was studied (Figure 5). Similar reactions of the disproportionation of compounds with the sulfur–selenium bond are known [62,63]. They are accelerated under the action of basic or acidic catalysts [63].

It was found that the reaction of thiaselenole **1** with thiol **2** can be carried out without a base in such aprotic bipolar solvent as DMF, which is able to bind evolved hydrogen bromide. Six-membered heterocycle **4** as a major product and five-membered heterocycle **5** as a minor product were formed in 1–1.5 h (Table 1, Runs 1, 2). The latter compound became the major product in 24 h (Table 1, Run 3). The six-membered product **4** was completely rearranged into five-membered heterocycle **5** in a 99% yield (Table 1, Run 4). Thus, new regioselective conditions for the quantitative preparation of heterocycle **5** were found.

Carrying out the reaction in the presence of an equimolar amount of NaHCO_3_ for 1 h made it possible to prevent the rearrangement and to direct the reaction to the formation of the six-membered heterocycle **4** in a 99% yield with high regioselectivity (Table 1, Run 5). These reaction conditions are very convenient (room temperature, 1 h), and the reaction regioselectively led to the desired product **4** in a quantitative yield. Increasing the reaction time under these conditions led to the partial rearrangement of product **4** into five-membered heterocycle **5** (Table 1, Run 6). Thus, the quantum chemical calculations assisted to find new reaction conditions that provided highly regioselective methods for the preparation of heterocycles **4** and **5** in quantitative yields, which did not require additional purification.

Further studies of the reaction were conducted out in acetonitrile. When the reaction was carried out in the presence of equimolar amounts of K_2_CO_3_ at 0 °C for 5 h, it was possible for the first time to detect the target selanyl sulfide **3** in a 9% yield along with the product of its disproportionation **6a** (46% yield) and heterocycle **4** (27% yield) (Table 1, Run 7). It is worth reminding that quantum chemical studies indicate the formation of selanyl sulfide **3** as the kinetic product (Scheme 4, Figure 2).

In the presence of pyridine as a base (0 °C, 3 h), the reaction led to products **4** and **5** in 25% and 61% yields, respectively (Table 1, Run 8). Even when using an equimolar amount of pyridine with respect to thiaselenole **1**, no traces of the pyridine quaternization product with thiaselenole were detected. The reaction of pyridine with thiaselenole **1** was studied by us previously and it was found that the quaternization was accompanied by rearrangement with the ring expansion of thiaselenole with the formation of (2,3-dihydro-1,4-thiaselenin-2-yl)pyridinium bromide [65].

A series of experiments on the reaction of thiaselenole **1** with sodium 1,3-benzothiazole-2-thiolate (anion **2^−^**) was carried out under homogeneous conditions in acetonitrile, varying the concentration of reagents, reaction time, and the method of the reaction mixture treatment (Table 1, Runs 9–12).

The most representative results are included in Table 1 (Runs 9–12). When the treatment consisted of dilution with water and extraction, the reaction mixture contained mainly thiaselenine **4** (45% yield) along with selanyl sulfide **3** (8% yield) and the product of its disproportionation diselenide **6a** (14% yield) with a complete conversion of thiaselenole **1** (Table 1, Run 9).

When another method of the reaction mixture treatment (filtering and acetonitrile removing) was used (Table 1, Run 10), the major product was diselenide **6a** (60% yield), the yield of thiaselenine **4** dropped from 45% to 8%, and the yield of selanyl sulfide **3** increased slightly to 11%. We assumed that increasing the yield of diselenide **6a** occurred on the stage of concentration of products upon removing acetonitrile under reduced pressure (Table 1, Run 10). Traces of sodium 1,3-benzothiazole-2-thiolate can present in the reaction mixture and act as the catalyst of the disproportionation reaction. This was confirmed by the experiment under the same conditions, when the resulted reaction mixture was divided into two equal parts, which were treated in a different mode. When the solvent was removed under reduced pressure from the first part, the 87% conversion of thiaselenole **1** and the formation of products **3**, **4**, and **6a** in 13%, 13%, and 54% yields, respectively, were observed (Table 1, Run 11). Thus, it made it possible to direct the reaction towards the preferential formation of selanyl sulfide **3** and the product of its disproportionation **6a** under these conditions. In the second half of the mixture, the reaction was stopped by dilution with water and extraction with CCl_4_ followed by the solvent removing under reduced pressure. The formation of products **4** (40% yield) and **6a** (30% yield) with a complete conversion of thiaselenole **1** was detected (Table 1, Run 12). Besides, 2,3-dihydro-1,4-thiaselenin-2-ol, the product of the competitive reaction of thiaselenole **1** with water in aqueous acetonitrile, was unexpectedly formed in a 10% yield. We previously obtained this compound by the reaction of thiaselenole **1** with water [64].

Based on the obtained results (Table 1, Runs 11, 12), it can be concluded that the initial reaction mixture contained diselenide **6a** (30% yield), which cannot be converted into other reaction products, and the formation of an additional amount of this compound (54% yield) is the result of disproportionation of selanyl sulfide **3** upon concentration of the solution by removing acetonitrile. The main product of the untreated reaction mixture is believed to be selanyl sulfide **3**, which is converted to thiaselenine **4** in aqueous acetonitrile. This is in good agreement with quantum chemical calculations (Figure 3).

**Scheme 5:**
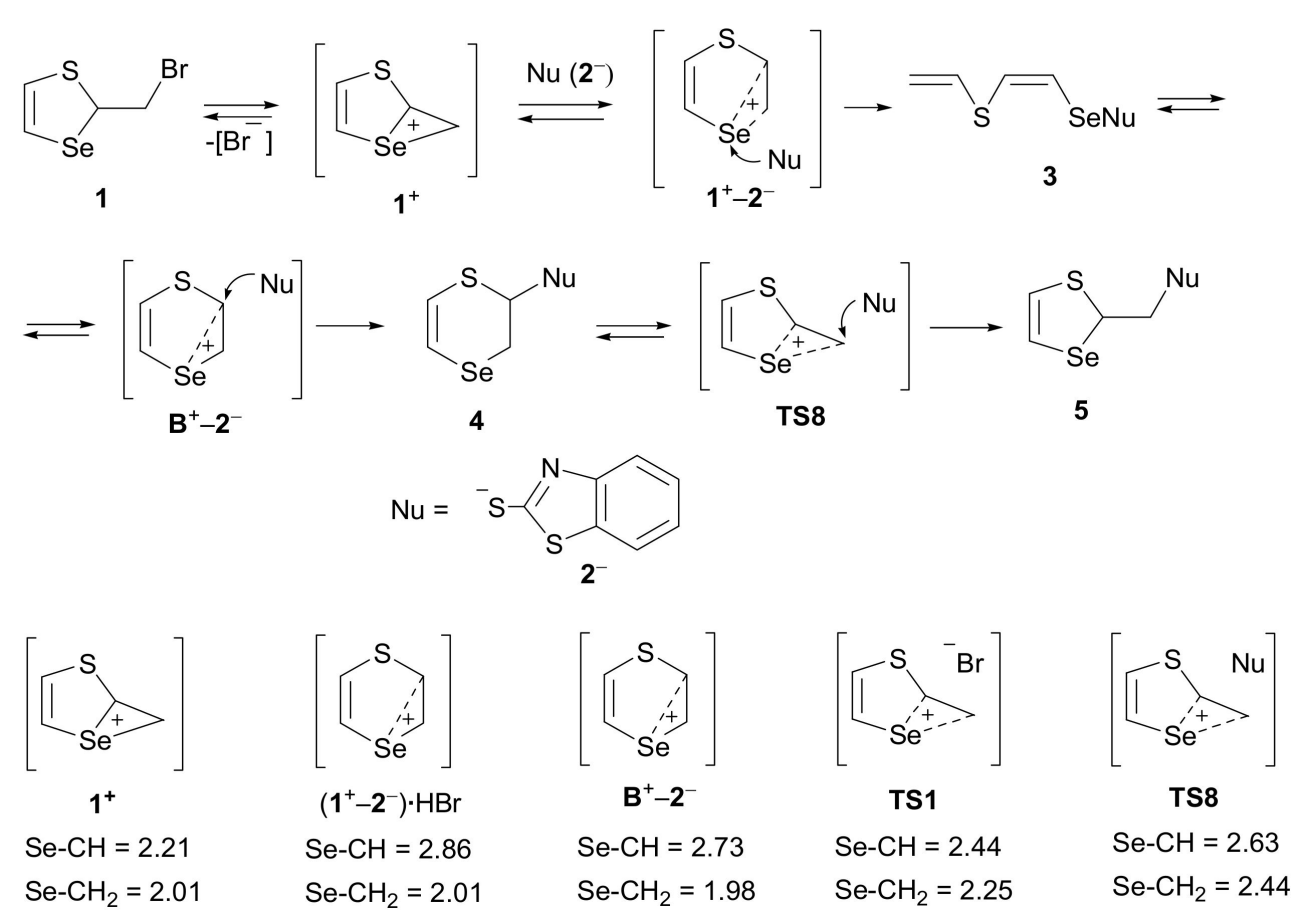
The simplified reaction pathway of multichannel regioselective nucleophilic substitution in thiaselenole **1** by thiol **2** proceeding via three centers of seleniranium intermediates based on the experimental data and quantum chemical calculations (bond lengths (in angstroms) are presented for the selected intermediates and transition states).

The simplified reaction pathway of multichannel regioselective reaction of thiaselenole **1** with thiol **2** based on the experimental data and quantum chemical calculations is presented in Scheme 5. The reaction of nucleophilic substitution in thiaselenole **1** with thiol **2** proceeds stepwise at three different centers of seleniranium intermediates. The first stage: the attack of thiolate anion at the selenium atom of the seleniranium cation generated from thiaselenole **1** proceeds with ring opening and the formation of selanyl sulfide **3**. It is important that the selenium atom conjugated with the sulfanylethenyl fragment is a soft electrophile, its reaction with thiolate anion **2^−^** (which is a soft nucleophile) proceeds with minimal energy consumption, and the formation of selanyl sulfide **3** as a kinetic product. On the second stage, the nucleophilic attack of thiolate anion occurs at the carbon atom of the CH group of seleniranium cation **B^+^**, which is generated from selanyl sulfide **3**. Seleniranium cation **B^+^** is characterized by the elongation of the Se–CH bond (2.73 Å) as compared to the **1^+^** cation (2.21 Å), which leads to its rupture by the nucleophilic attack and the formation of heterocycle **4**. The third stage: the attack of thiolate anion at the carbon atom of the CH_2_ group of the seleniranium cation (transition state **S8**) generated from six-membered thiaselenine **4**. The third stage is accompanied by rearrangement, which proceeds with the ring contraction producing five-membered thermodynamic product **5**.

The stages were optimized and heterocycles **4** and **5** were obtained in quantitative yields. Increasing the concentration of selanyl sulfide **3** in solution led to its disproportionation and the formation of symmetric products **6a** and **6b**. The experimental data are in good agreement with quantum chemical calculations including the analysis of the thermodynamic characteristics of the reaction.

## 3. Materials and Methods

### 3.1. Computational Analysis

The potential energy surface of the reaction of thiaselenole **1** with thiol **2** in the framework of the GAUSSIAN 09 software package was investigated using the basic set 6-311 + G (d, p) [66]. The calculations of molecular structures and the study of the gradient channels connecting them (Scheme 4) were carried out using the density functional theory (DFT) with the three-parameter B3LYP functional [67]. Stationary points were identified by the analysis of the Hessian matrices. The search and localization of transient states (**TS**) was carried out by the method of synchronous transit QST [68]. All the results obtained refer to the gas phase. The analysis of vibration frequencies at the saddle point was carried out and the correspondence of the critical points to the gradient line was proved.

### 3.2. General Information

^1^H (400.1 MHz), ^13^C (100.6 MHz), ^15^N (40.56 MHz), and ^77^Se (76.3 MHz) NMR spectra were recorded on a Bruker DPX-400 spectrometer (Bruker BioSpin GmbH, Rheinstetten, Germany) in 5%–10% solution in CDCl_3_ referenced to HMDS (^1^H−NMR, internal), the residual CDCl_3_ peak (77.00 ppm for ^13^C−NMR), MeNO_2_ (^15^N−NMR, external), and Me_2_Se (^77^Se−NMR, external). Mass spectra were recorded on an Agilent 5975 Series Gas Chromatograph/Mass Selective Detector (GC/MSD) system (Agilent Technologies, Inc., Santa Clara, CA, USA) with electron impact (EI) ionization at 70 eV. The C, H, N, and S elemental analyses were performed on a Thermo Scientific FLASH 2000 Organic Elemental Analyzer (Thermo Fisher Scientific Inc., Milan, Italy). The content of selenium was assessed by iodometric titration. Melting points were determined on a Kofler Hot-Stage Microscope PolyTherm A apparatus (Wagner and Munz GmbH, München, Germany) and uncorrected.

To start, 2-bromomethyl-1,3-thiaselenole **1** was prepared from divinyl sulfide and SeBr_2_ according to the previously described procedure [57]. A commercial 1,3-benzothiazole-2-thiol (**2**) (Sigma-Aldrich, St. Louis, MO, USA) and dried, freshly distilled solvents were used in the reactions. All syntheses using acetonitrile were carried out under an argon atmosphere. Acetonitrile was degassed with argon before the use.

### 3.3. General Procedure for the Reaction of Thiaselenole ***1*** with Thiol ***2*** in DMF

#### 3.3.1. Without Base

A solution of thiol **2** (334 mg, 2 mmol) in DMF (3 mL) was added to a solution of thiaselenole **1** (488 mg, 2 mmol) in DMF (3 mL). The mixture was stirred at room temperature for the prescribed time (Table 1, Runs 1–4), diluted with cold water (40 mL), and extracted with EtOAc (5 × 5 mL). The organic phase was washed with water (5 × 5 mL) and dried over CaCl_2_. After removing the solvent in a vacuum, the residue (a yellow powder) was analyzed by ^1^H−NMR (Table 1). Analysis examples can be found in the Supplementary Materials, Figures S14–S19.

#### 3.3.2. In the Presence of NaHCO_3_

A solution of thiol **2** (167 mg, 1 mmol) in DMF (2 mL) and NaHCO_3_ (84 mg, 1 mmol) were added to a solution of thiaselenole **1** (244 mg, 1 mmol) in DMF (2 mL). The mixture was stirred at room temperature for the prescribed time (Table 1, Runs 5, 6), diluted with cold water (20 mL), and extracted with EtOAc (3 × 5 mL). The organic phase was washed with water (3 × 5 mL) and dried over CaCl_2_. After removing the solvent in a vacuum, the residue (a yellow powder) was analyzed by ^1^H−NMR (Table 1). Analysis examples can be found in the Supplementary Materials, Figures S14–S19.

### 3.4. 2-(2,3-Dihydro-1,4-thiaselenin-2-ylsulfanyl)-1,3-benzothiazole ***4***

A solution of thiol **2** (167 mg, 1 mmol) in DMF (2 mL) and NaHCO_3_ (84 mg, 1 mmol) were added to a solution of thiaselenole **1** (244 mg, 1 mmol) in DMF (2 mL) (Table 1, Run 5). The mixture was stirred at room temperature for 1 h, diluted with cold water (20 mL), and extracted with EtOAc (3 × 5 mL). The organic phase was washed with water (3 × 5 mL) and dried over CaCl_2_. Removing the solvent in vacuum led to compound **4** as a yellow powder. Yield: 327 mg (99%). M.p. 90–91 °C. The NMR data can be found in the Supplementary Materials.

### 3.5. 2-[(1,3-Thiaselenol-2-ylmethyl)sulfanyl]-1,3-benzothiazole ***5***

A solution of thiol **2** (334 mg, 2 mmol) in DMF (3 mL) was added to a solution of thiaselenole **1** (488 mg, 2 mmol) in DMF (3 mL) (Table 1, Run 4). The mixture was stirred at room temperature for 48 h, diluted with cold water (40 mL), and extracted with EtOAc (5 × 5 mL). The organic phase was washed with water (5 × 5 mL) and dried over CaCl_2_. After removing the solvent in a vacuum, compound **5** was obtained as a white-yellow powder. Yield: 653 mg (99%). M.p. 109–110 °C. The NMR data can be found in the Supplementary Materials.

### 3.6. Reaction of Thiaselenole ***1*** with Benzothiazole ***2*** in Acetonitrile in the Presence of K_2_CO_3_

Thiol **2** (302 mg, 1.81 mmol) and K_2_CO_3_ (250 mg, 1.81 mmol) were added to a solution of thiaselenole **1** (442 mg, 1.81 mmol) in acetonitrile (10 mL) (Table 1, Run 7). The mixture was stirred at 0 °C for 5 h. The mixture was filtered, and the solvent was removed in a vacuum. The residue (355 mg, a yellow oil) contained (according to ^1^H−NMR data) product **3** (54 mg, 9% yields), product **4** (164 mg, 27% yields), and product **6a** (137 mg, 46% yields) (a 15:46:39 molar ratio of compounds **3**, **4**, and **6a**). Compounds **3**, **4**, and **6a** were identified by ^1^H−NMR (the analysis of the reaction mixture can be found in the Supplementary Materials, Figures S14–S16).

### 3.7. Reaction of Thiaselenole ***1*** with Benzothiazole ***2*** in Acetonitrile in the Presence of Pyridine 

Pyridine (111 mg, 1.41 mmol) was added to a solution of thiaselenole **1** (344 mg, 1.41 mmol) in acetonitrile (8 mL) (Table 1, Run 8). The mixture was cooled to 0 °C and thiol **2** (235 mg, 1.41 mmol) was added. The mixture was stirred at 0 °C for 3 h, diluted with cold water (10 mL), and extracted with chloroform (3 × 5 mL). The organic phase was dried over CaCl_2_ and the solvent was removed in a vacuum. The residue (397 mg, a yellow powder) contained (according to ^1^H−NMR data) products **4** (115 mg, 25% yield) and **5** (282 mg, 61% yield) (a 29:71 molar ratio of compounds **4** and **5**).

### 3.8. Sodium 1,3-Benzothiazole-2-thiolate (Sodium Thiolate of Benzothiazole ***2***)

Powdered NaOH (80%, 300 mg, 6.00 mmol) was added to a solution of thiol **2** (1002 mg, 6.00 mmol) in dry EtOH (60 mL). The mixture was stirred at room temperature for 0.5 h and the solvent was removed in a vacuum. Sodium 1,3-benzothiazole-2-thiolate was obtained as a light-yellow powder. Yield: 1134 mg (~100%). M.p. 327–328 °C.

### 3.9. Reaction of Thiaselenole ***1*** with Sodium 1,3-Benzothiazole-2-thiolate

- Table 1, Run 9

A solution of thiaselenole **1** (244 mg, 1 mmol) in acetonitrile (6 mL) was added to sodium 1,3-benzothiazole-2-thiolate (189 mg, 1.00 mmol). The mixture was stirred at room temperature for 10 min, diluted with cold water (20 mL), and extracted with chloroform (3 × 10 mL). The organic phase was filtered, washed with water, dried over CaCl_2_, and the solvent was removed in a vacuum. The residue (224 mg, a yellow oil) contained (according to ^1^H−NMR data) compounds **3** (27 mg, 8% yield), **4** (150 mg, 45% yield), and **6a** (47 mg, 14% yield) (a 12:67:21 molar ratio of compounds **3**, **4**, and **6a**).

- 1,2-Bis[(Z)-2-(Vinylsulfanyl)ethenyl] Diselenide **6** (Table 1, Run 10)

A solution of sodium 1,3-benzothiazole-2-thiolate (189 mg, 1 mmol) in acetonitrile (3 mL) was added to a solution of thiaselenole **1** (244 mg, 1 mmol) in acetonitrile (3 mL). The mixture was stirred at room temperature for 0.5 h. The mixture was filtered, and the solvent was removed in a vacuum. The residue (162 mg, a yellow oil) contained (according to ^1^H−NMR data) compounds **3** (37 mg, 11% yield), **4** (26 mg, 8% yield), and **6a** (99 mg, 60% yield) (a 23:16:61 molar ratio of compounds **3**, **4**, and **6a**). Compound **6a** (91 mg, 55% yield) was isolated by column chromatography on silica gel (eluent: hexane). ^1^H−NMR (400 MHz, CDCl_3_), *δ*: 7.04 (d, ^3^*J* = 7.9 Hz, 2H, SCH=CHSe), 6.63 (d, ^3^*J* = 7.9 Hz, 2H, SCH=CHSe), 6.42 (dd, ^3^*J*_cis_ = 9.8 Hz, ^3^*J*_trans_ = 16.4 Hz, 2H, H_2_C=CHS), 5.33 (d, ^3^*J*_cis_ = 9.8 Hz, 1H, H_2_C=CHS), 5.28 (d, ^3^*J*_trans_ = 16.4 Hz, 1H, H_2_C=CHS). ^13^C−NMR (100 MHz, CDCl_3_) *δ*: 114.37 (H_2_C=CHS), 124.95 (SCH=CHSe), 127.72 (SCH=CHSe), 129.61 (H_2_C=CHS). ^77^Se−NMR (76 MHz, CDCl_3_): δ 418.9 ppm. MS (EI) *m*/*z*: 330 (6%) [M]^+^, 165 (75%), 151 (32%), 133 (6%), 85 (100%), 58 (51%), 45 (59%). Found, %: C 29.47; H 2.95, S 19.39, Se 47.87. C_8_H_10_S_2_Se_2_. Calculated, %: C 29.28; H 3.07, S 19.54, Se 48.11.

### 3.10. Reaction of Thiaselenole ***1*** with Sodium 1,3-Benzothiazole-2-thiolate in Acetonitrile at High Dilution. Synthesis of (Z)-2-[(1,3-Benzothiazol-2-ylsulfanyl)selanyl]ethenyl Vinyl Sulfide (***3***) 

A solution of sodium 1,3-benzothiazole-2-thiolate (280 mg, 1.48 mmol) in acetonitrile (13 mL) was added to a solution of thiaselenole **1** (361 mg, 1.48 mmol) in acetonitrile (14 mL). The mixture was stirred at room temperature for 10 min. The mixture was filtered and divided into two equal parts (Table 1, Runs 11, 12).

- Table 1, Run 11

The solvent from the first part of the mixture was removed in a vacuum. The residue (153 mg, a yellow oil) contained (according to ^1^H−NMR data) thiaselenole **1** (23 mg, 87% conversion), and compounds **3** (32 mg, 13% yield), **4** (32 mg, 13% yield), and **6a** (66 mg, 54% yield) (a 19:20:20:41 molar ratio of compounds **1**, **3**, **4**, and **6a**). Compounds **1**, **3**, **4**, and **6a** were identified by ^1^H−NMR (the analysis of the reaction mixture can be found in the Supplementary Materials, Figures S17 and S18).

- Table 1, Run 12

The second part of the mixture was diluted with cold water (7 mL) and extracted with CCl_4_ (7 mL). The organic phase was dried over MgSO_4_ and the solvent was removed in a vacuum. The residue (147 mg, a yellow oil) contained (according to ^1^H−NMR data) products **4** (98 mg, 40% yield), **6a** (36 mg, 30% yield), and 2,3-dihydro-1,4-thiaselenin-2-ol (13 mg, 10% yield) (a 63:23:14 molar ratio of compounds **4**, **6a**, and 2,3-dihydro-1,4-thiaselenin-2-ol). Compounds **4**, **6a**, and 2,3-dihydro-1,4-thiaselenin-2-ol [64] were identified by ^1^H−NMR (the analysis of the reaction mixture can be found in the Supplementary Materials, Figure S19).

## 4. Conclusions

Three successive stages of nucleophilic substitution reaction of thiol **2** with thiaselenole **1** proceeding via the generation of seleniranium cation were optimized. This determines the ease of ring opening on attacking the soft nucleophile **2** at the soft electrophile—the selenium atom in seleniranium cation with the formation of labile selanyl sulfide **3**. The subsequent stage is accompanied by the generation of seleniranium cation from selanyl sulfide **3** by the nucleophilic attack at the carbon atom of the CH group, leading to six-membered thiaselenine **4**. The five-membered thiaselenole **5** is formed by the nucleophilic attack at the carbon atom of the CH_2_ group of the seleniranium cation generated from thiaselenine **4**. The results of quantum chemical studies of these unprecedented reactions are in good agreement with the experimental data.

Based on obtained results, regioselective methods for preparation of heterocycles **4** and **5** in quantitative yields at room temperature were developed. Due to the disproportionation of product **3**, a symmetric highly unsaturated diselenide of (*Z,Z*)-stereochemistry **6a** is formed. Compound **6a** is the promising reagent for organoselenium chemistry. The reduction of diselenide **6a** leads to the generation of (*Z*)-vinylsulfanylethenylselenide anion, which can be involved in further nucleophilic reactions.

The reaction products combine sulfur/selenium- and nitrogen-containing heterocycles, and these combinations represent new scaffolds, which can find applications in organochalcogen and medicinal chemistry. For example, the selenium moiety of the obtained compounds can bring glutathione peroxidase-like activity to the products. It is known that a number of organic selenides exhibit high glutathione peroxidase-like activity [17,18,19,20].

## Figures and Tables

**Table 1:** The influence of conditions on the yields and the ratio of products in the reaction of thiaselenole **1** with thiol **2** (or thiolate).

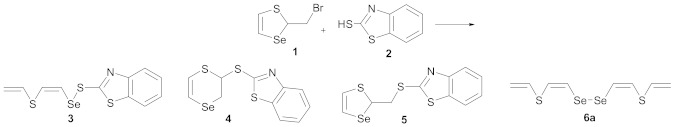										
Run	Solvent	Base, 1 Eq	T, °C	Time, h	Molar Ratio ^a^ 1:4:5:3:6a	Yield ^a^ of 4, %	Yield ^a^ of 5, %	Yield ^a^ of 3, %	Yield ^a^ of 6a, %	Conversion a of 1, %
1	DMF	−	25	1	0:96:4:0:0	91	4	−	−	100
2	DMF	−	25	1.5	0:85:15:0:0	81	14	−	−	100
3	DMF	−	25	24	0:5:95:0:0	5	92	−	−	100
**4**	**DMF**	−	**25**	**48**	**0:0:100:0:0**	−	**99**	−	−	**100**
**5**	**DMF**	**NaHCO_3_**	**25**	**1**	**0:100:0:0:0**	**99**	−	−	−	**100**
6	DMF	NaHCO_3_	25	24	0:39:61:0:0	39	60	−	−	100
7	MeCN	K_2_CO_3_	0	5	0:46:0:15:39	27	−	9	46	100
8	MeCN	C_5_H_5_N	0	3	0:29:71:0:0	25	61	−	−	100
9 ^b^	MeCN	RSNa^c^	25	0.17	0:67:0:12:21	45	−	8	14	100
**10** ^d^	**MeCN**	**RSNa^c^**	**25**	**0.5**	**0:16:0:23:61**	**8**	−	**11**	**60**	**100**
**11** ^e^	**MeCN**	**RSNa^c^**	**25**	**0.17**	**19:20:0:20:41**	**13**	−	**13**	**54**	**87**
12 ^e^	MeCN	RSNa^c^	25	0.17	0:63:0:0:23	40	−	−	30	100

^a^ Due to the lability of intermediate compounds, the ratio and yields of products **1**, **3**–**6** were determined by NMR in the reaction mixture. ^b^ A solution of thiaselenole **1** in acetonitrile (6 mL) was added to a solution of sodium 1,3-benzothiazole-2-thiolate. The reaction mixture was diluted with water and extracted with chloroform. ^c^ Sodium 1,3-benzothiazole-2-thiolate was used as a reagent instead of 1,3-benzothiazole-2-thiol. ^d^ A solution of sodium 1,3-benzothiazole-2-thiolate in acetonitrile (3 mL) was added to a solution of thiaselenole **1** in acetonitrile (3 mL). The reaction mixture was stirred for 0.5 h and the solvent was removed in vacuum. ^e^ The reaction was carried out with 1.48 mmol of the reagents in 27 mL of acetonitrile. The reaction mixture was divided into two equal parts. From one of them the solvent was removed in a vacuum, and unconverted thiaselenole **1** (13% yield, Run 11) was detected along with the reaction products. The second part was diluted with water followed by extraction with CCl_4_, drying and removing the solvent in vacuum (Run 12). The reaction mixture contained 2,3-dihydro-1,4-thiaselenin-2-ol (10% yield) [64].

## Data Availability

Data is available in this article and Supplementary Materials.

## Supplementary Materials

The following are available online, synthesis and ^1^H−, ^13^C−, and ^77^Se−NMR spectra of 2-bromomethyl-1,3-thiaselenole (**1**), ^1^H−, ^13^C−, ^15^N−, and ^77^Se−NMR spectra of the obtained compounds (Figures S1–S13) and ^1^H NMR spectra of the reaction mixtures (Table 1, Runs 7, 11, 12, Figures S14–S19) and results of quantum chemical calculations (Tables S1–S25).
