# Supplementary material for: Quantum Chemical and Experimental Studies of an Unprecedented Reaction Pathway of Nucleophilic Substitution of 2-Bromomethyl-1,3-thiaselenole with 1,3-Benzothiazole-2-thiol Proceeding Stepwise at Three Different Centers of Seleniranium Intermediates
Source: Molecules. 2021 Nov 4;26(21):6685. doi: 10.3390/molecules26216685 (PMC8588063; doi:10.3390/molecules26216685)
Supplement: Supplementary file 1 [file molecules-26-06685-s001.zip › molecules-1421791-supplementary.pdf]

Supplementary Materials

Quantum Chemical and Experimental Studies of an Unprecedented Reaction Pathway of Nucleophilic Substitution of 2-Bromomethyl-1,3-thiaselenole with 1,3-Benzothiazole-2-thiol Proceeding Stepwise at Three Different Centers of Seleniranium Intermediates

Svetlana V. Amosova,* Vladimir A. Shagun, Nataliya A. Makhaeva, Irina A. Novokshonova and Vladimir A. Potapov

A. E. Favorsky Irkutsk Institute of Chemistry, Siberian Division of The Russian Academy of Sciences, 1 Favorsky Str., Irkutsk 664033, Russia; shagun@irioch.irk.ru (V.A.S.); ggm@ irioch.irk.ru (N.A.M.); Lotelen@yandex.ru (I.A.N.); v.a.potapov@mail.ru (V.A.P.).

* Correspondence: amosova@irioch.irk.ru (S.V.A.)

Table of Contents pages

Synthesis of 2-bromomethyl-1,3-thiaselenole 1 2

2-(2,3-Dihydro-1,4-thiaselenin-2-ylsulfanyl)-1,3-benzothiazole 4 3

2-[(1,3-Thiaselenol-2-ylmethyl)sulfanyl]-1,3-benzothiazole 5 3

NMR spectra of the compounds 1, 4, 5 and 6a (Figures S1-S13) 4-16

The analysis of NMR spectra of Runs 7, 11 and 12 (Table 1) (Figures S14-S19) 17-22

Results of quantum chemical calculations (Tables S1-S25) 23-41

Starting 2-bromomethyl-1,3-thiaselenole (1) was prepared from $SeBr_2$ and divinyl sulfide according to the previously described procedure: Amosova, S.V.; Novokshonova, I.A.; Penzik, M.V.; Filippov, A.S.; Albanov, A.I.; Potapov, V.A. Reaction of 2-bromomethyl-1,3-thiaselenole with thiourea: En route to the first representatives of 2-(organylsulfanyl)-2,3-dihydro-1,4-thiaselenines. ***Tetrahedron Lett.*** 2017, ***58***, 4381-4383. https://doi.org/10.1016/j.tetlet.2017.10.011]

2-Bromomethyl-1,3-thiaselenole (1). Modified procedure for the synthesis 2-bromomethyl-1,3-thiaselenole 1. Dried, freshly distilled and argon-degassed CCl_4 was used in the reaction. The reaction was carried out under argon atmosphere. A solution of bromine (5.85 g, 36.63 mmol) in CCl_4 (30 mL) was added dropwise to powdered selenium (2.89 g, 36.63 mmol, carefully ground) in CCl_4 (70 mL) over 15-20 min under an Ar atmosphere and the mixture stirred for 2 h. The formed solution of $SeBr_2$ in CCl_4 (100 mL) and divinyl sulfide (3.15 g, 36.63 mmol) in CCl_4 (10 mL) were simultaneously added dropwise over 1 h to a flask containing CCl_4 (15-20 mL) so that the molar ratio of both reagents in the mixture was 1:1. The reaction mixture was stirred for 3 h, then a solution of pyridine (3.47 g, 43.92 mmol) in CCl_4 (10 mL) was added over 10-15 min, and the mixture stirred overnight. The reaction mixture was filtered, and the solution stirred for 24 h and filtered again. Most of the solvent was removed by rotary evaporation. The residue (25-30 mL) was allowed to stand for 1 h and filtered. The solvent and the remaining pyridine were removed from the filtrate ***in vacuo*** to give high purity thiaselenole 1 (6.35 g, yellow oil) in 71% yield.

^{1}H−NMR (400 MHz, $CDCl_3$): δ: 6.63 (d, $^3J_{H,H}$ = 6.3 Hz, $^2J_{Se,H}$ = 49.3 Hz, 1H, SeC<u>H</u>=HCS), 6.40 (d, $^3J_{H,H}$ = 6.3 Hz, $^3J_{Se,H}$ = 21.0 Hz, 1H, SeCH=<u>H</u>CS), 5.06 (dd, $^3J_{H,H}$ = 7.3 Hz, $^3J_{H,H}$ = 8.2 Hz, $^2J_{Se,H}$ = 24.0 Hz, 1H, SCHSe), 3.63 (dd, $^2J_{H,H}$ = 10.0 Hz, $^3J_{H,H}$ = 8.2 Hz, 1H, CH_2Br), 3.57 (dd, $^2J_{H,H}$ = 10.0 Hz, $^3J_{H,H}$ = 7.3 Hz, 1H, CH_2Br); ^{13}C−NMR (100 MHz, $CDCl_3$): δ: 119.59 (SeCH=H<u>C</u>S), 113.46 ($^1J_{Se,C}$ = 106.6 Hz, Se<u>C</u>H=HCS), 47.65 ($^1J_{Se,C}$ = 71.3 Hz, SCHSe), 37.51 (CH_2Br); ^{77}Se−NMR (76 MHz, $CDCl_3$): δ: 521.57; MS (EI) ***m/z***: 244 ($[M]^+$, 21%), 151 (74%), 84 (99%), 58 (87%), 49 (100%); Anal. Calcd for C_4H_5BrSSe: C 19.69, H 2.07, Br 32.75, S 13.14, Se 32.36. Found: C 19.43, H 2.18, Br 32.64, S 13.44, Se 32.21.

2-(2,3-Dihydro-1,4-thiaselenin-2-ylsulfanyl)-1,3-benzothiazole (4). Yield: 99%, a yellow powder, m.p. 90-91 °C. ^{1}H–NMR (400 MHz, $CDCl_3$): δ: 7.85 (d, $^3J_{H,H}$ = 8.2 Hz, 1H, H-5), 7.70 (d, $^3J_{H,H}$ = 8.0 Hz, 1H, H-8), 7.38 (dd, $^3J_{H\text{-}5,H\text{-}6}$ = 8.2 Hz, $^3J_{H\text{-}6,H\text{-}7}$ = 7.5 Hz, 1H, H-6), 7.26 (dd, $^3J_{H\text{-}7,H\text{-}8}$ = 8.0 Hz, $^3J_{H\text{-}6,H\text{-}7}$ = 7.5 Hz, 1H, H-7), 6.47 (d, $^3J_{H,H}$ = 10.0 Hz, $^2J_{Se,H}$ = 50.0 Hz, 1H, SeC<u>H</u>=CHS), 6.39 (d, $^3J_{H,H}$ = 10.0 Hz, 1H, SeCH=C<u>H</u>S), 5.67 (dd, $^3J_{H,H}$ = 6.9 Hz, $^3J_{H,H}$ = 2.3 Hz, 1H, SCHS), 3.68 (dd, $^2J_{H,H}$ = 12.2 Hz, $^3J_{H,H}$ = 2.3 Hz, 1H, $SeCH_2$), 3.35 (dd, $^2J_{H,H}$ = 12.2 Hz, $^3J_{H,H}$ = 6.9 Hz, 1H, $SeCH_2$); ^{13}C–NMR (100 MHz, $CDCl_3$): δ: 163.34 (C-2), 152.71 (C-4), 135.26 (C-9), 125.97 (C-6), 124.48 (C-7), 121.65 (C-5), 120.93 (C-8), 117.81 ($^2J_{C,Se}$ = 8.2 Hz, SeCH=<u>C</u>HS), 110.02 ($^1J_{C,Se}$ = 116.3 Hz, Se<u>C</u>H=CHS), 45.69 (SCHS), 25.15 ($^1J_{C,Se}$ = 64.6 Hz, $SeCH_2$); ^{15}N–NMR (40 MHz, $CDCl_3$): δ: –79.84; ^{77}Se–NMR (76 MHz, $CDCl_3$): δ: 181.05; MS (EI) m/z: 332 ($[M]^+$, 4%), 272 (6%), 192 (32%), 164 (100%), 151 (7%), 135 (7%), 122 (8%), 108 (24%), 84 (58%), 69 (16%), 63 (10%), 58 (25%), 53 (10%), 45 (23%), 39 (9%). Anal. calcd for $C_{11}H_9NS_3Se$: C 39.99, H 2.75, N 4.24, S 29.12, Se 23.90. Found: C 39.82, H 2.60, N 4.08, S 28.97, Se 24.15.

2-[(1,3-Thiaselenol-2-ylmethyl)sulfanyl]-1,3-benzothiazole (5). Yield: 99%, a white-yellow powder, m.p. 109-110 °C. ^{1}H–NMR (400 MHz, $CDCl_3$): δ: 7.86 (ddd, $^3J_{H\text{-}5,H\text{-}6}$ = 8.3 Hz, $^4J_{H\text{-}5,H\text{-}7}$ = 1.0 Hz, $^5J_{H\text{-}5,H\text{-}8}$ = 0.6 Hz, 1H, H-5), 7.74 (ddd, $^3J_{H\text{-}7,H\text{-}8}$ = 8.1 Hz, $^4J_{H\text{-}6,H\text{-}8}$ = 1.2 Hz, $^5J_{H\text{-}5,H\text{-}8}$ = 0.6 Hz, 1H, H-8), 7.41 (ddd, $^3J_{H\text{-}5,H\text{-}6}$ = 8.3 Hz, $^3J_{H\text{-}6,H\text{-}7}$ = 7.3 Hz, $^4J_{H\text{-}6,H\text{-}8}$ = 1.2 Hz, 1H, H-6), 7.29 (ddd, $^3J_{H\text{-}7,H\text{-}8}$ = 8.1 Hz, $^3J_{H\text{-}6,H\text{-}7}$ = 7.3 Hz, $^4J_{H\text{-}5,H\text{-}7}$ = 1.0 Hz, 1H, H-7), 6.64 (d, $^3J_{H,H}$ = 6.6 Hz, $^2J_{Se,H}$ = 48.4 Hz, 1H, SeC<u>H</u>=CHS), 6.42 (d, $^3J_{H,H}$ = 6.6 Hz, 1H, SeCH=C<u>H</u>S), 5.34 (t, $^3J_{H,H}$ = 7.2 Hz, 1H, SCHSe), 3.73 (dd, $^2J_{H,H}$ = 13.8 Hz, $^3J_{H,H}$ = 7.2 Hz, 1H, CH_2S), 3.67 (dd, $^2J_{H,H}$ = 13.8 Hz, $^3J_{H,H}$ = 7.2 Hz, 1H, CH_2S); ^{13}C–NMR (100 MHz, $CDCl_3$) δ: 165.15 (C-2), 153.04 (C-4), 135.40 (C-9), 126.08 (C-6), 124.42 (C-7), 121.62 (C-5), 121.01 (C-8), 119.85 (SeCH=<u>C</u>HS), 113.74 ($^1J_{C,Se}$ = 106.0 Hz, Se<u>C</u>H=CHS), 46.75 ($^1J_{C,Se}$ = 70.0 Hz, SCHSe), 41.94 ($^2J_{C,Se}$ = 7.8 Hz, CH_2S); ^{15}N–NMR (40 MHz, $CDCl_3$): δ: –83.70; ^{77}Se–NMR (76 MHz, $CDCl_3$): δ 517.6; MS (EI) m/z: 332 ($[M]^+$, 2%), 192 (19%), 164 (100%), 151 (44%), 135 (5%), 122 (11%), 108 (27%), 102 (7%), 95 (4%), 84 (34%), 69 (15%), 63 (10%), 58 (24%), 53 (6%), 45 (27%), 39 (10%). Anal. calcd for $C_{11}H_9NS_3Se$: C 39.99, H 2.75, N 4.24, S 29.12, Se 23.90. Found: C 39.94, H 2.65, N 4.14, S 29.27, Se 23.77.

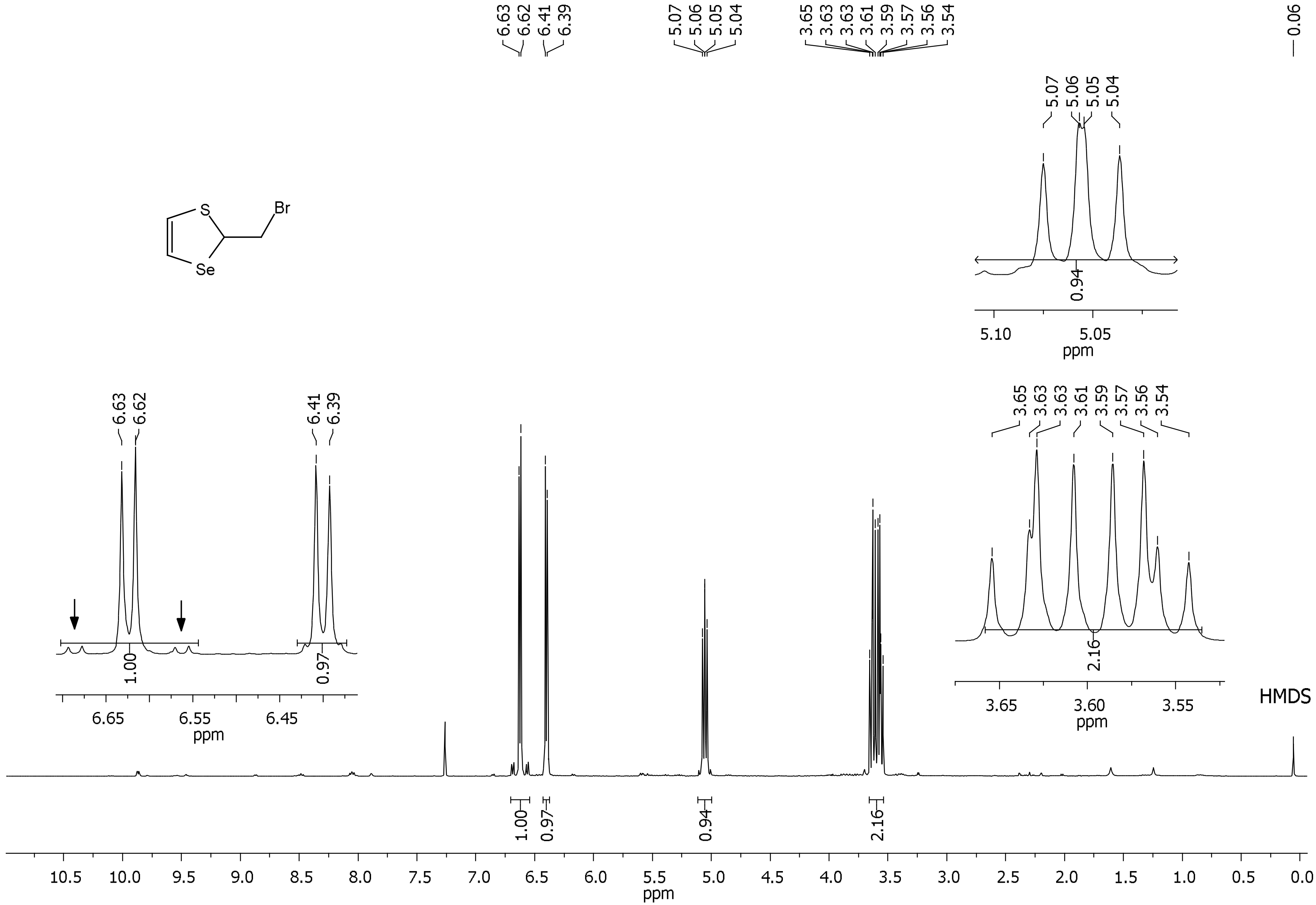

Figure S1. ^{1}H–NMR spectrum of 2-bromomethyl-1,3-thiaselenole (1)

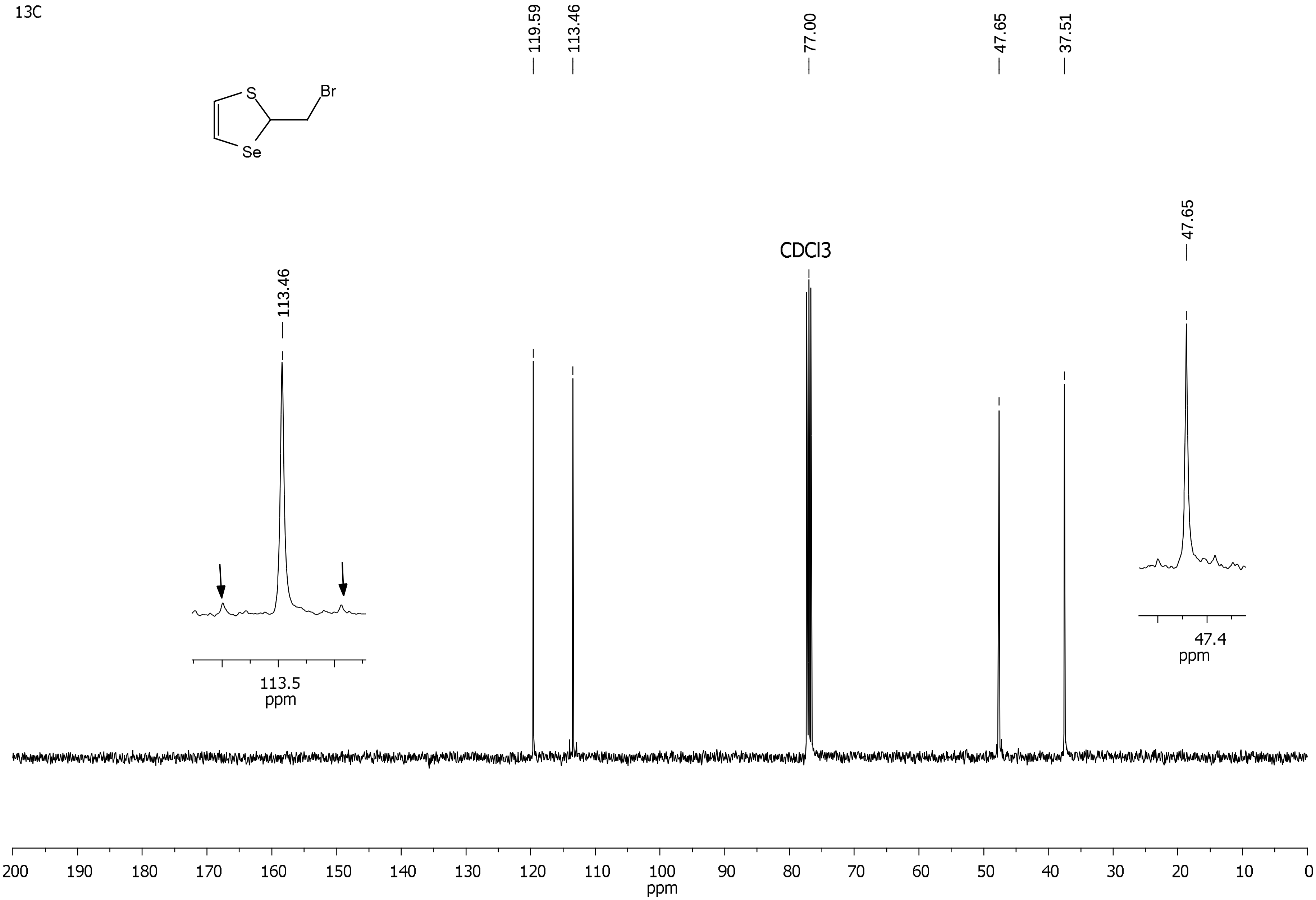

Figure S2. ^{13}C–NMR spectrum of 2-bromomethyl-1,3-thiaselenole (1)

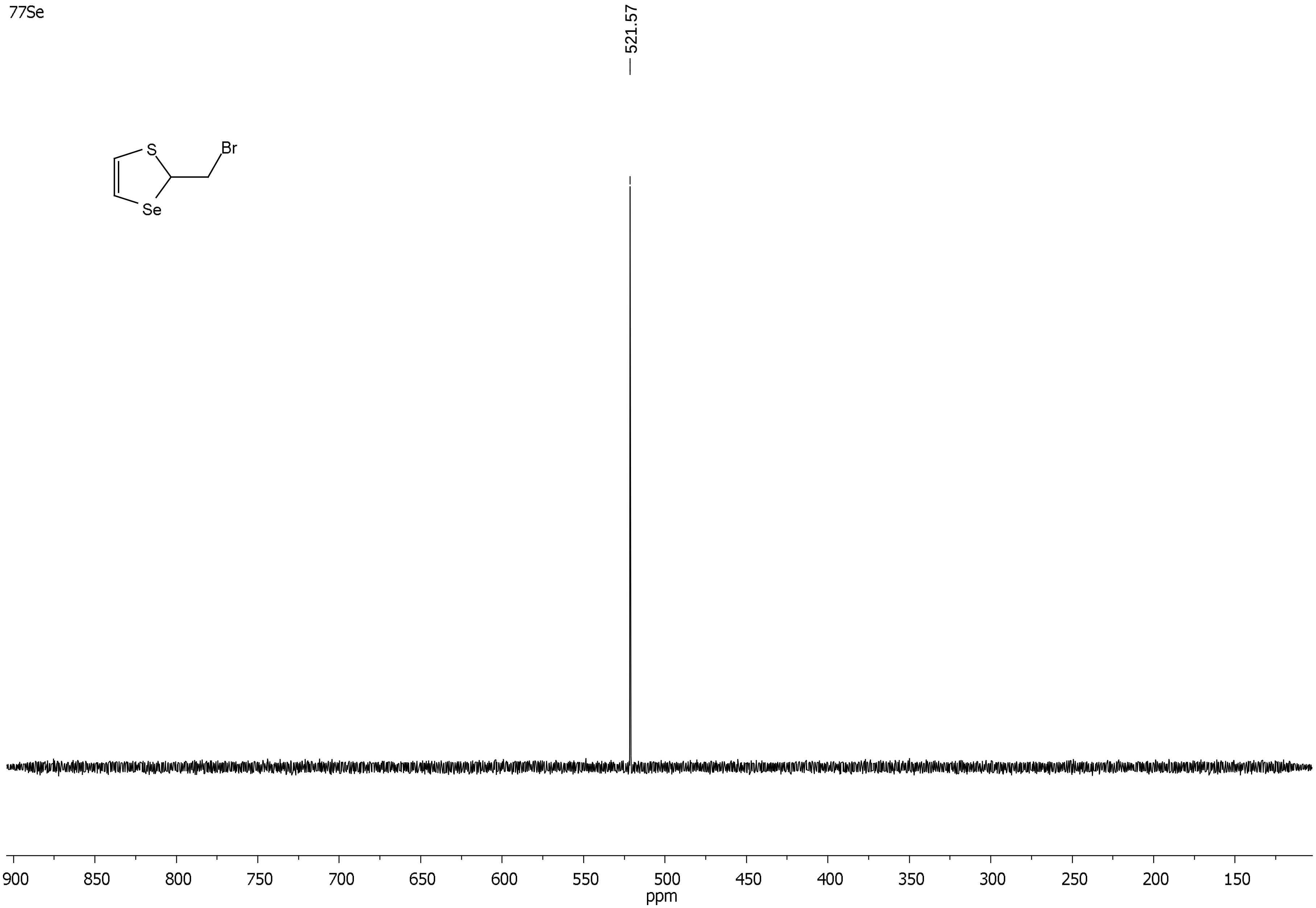

Figure S3. ^{77}Se–NMR spectrum of 2-bromomethyl-1,3-thiaselenole (1)

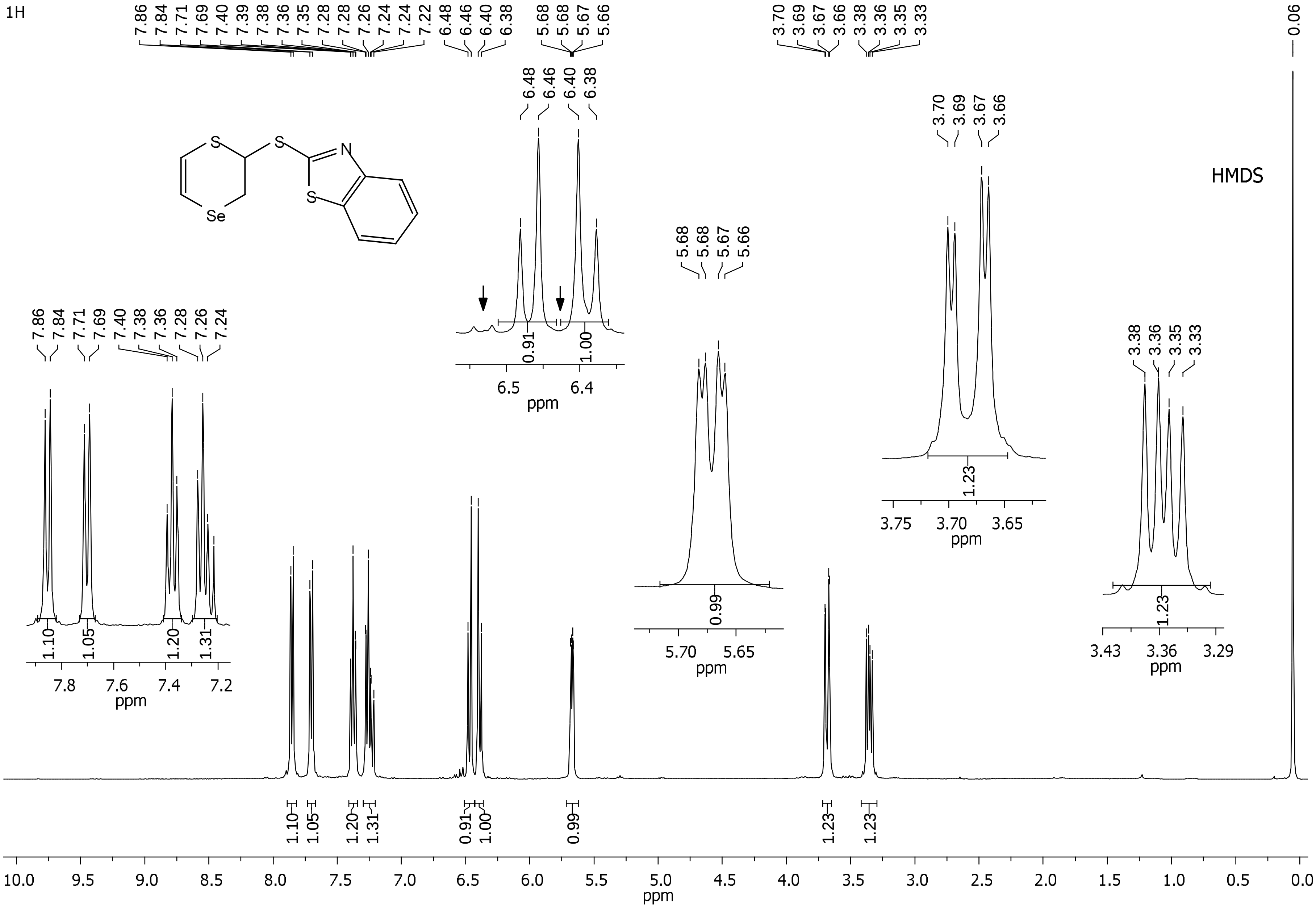

Figure S4. ^{1}H–NMR spectrum of 2-(2,3-dihydro-1,4-thiaselenin-2-ylsulfanyl)-1,3-benzothiazole (4)

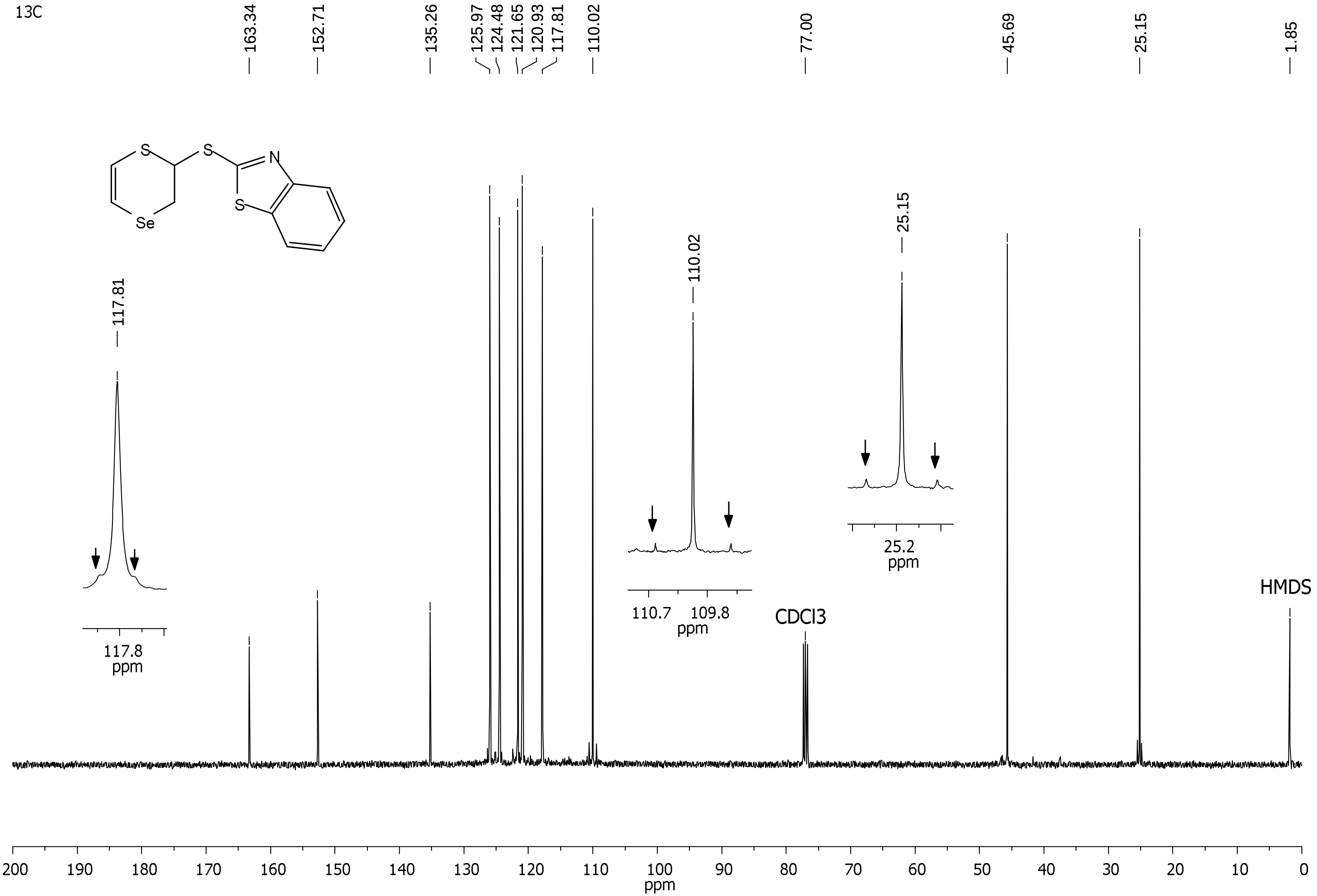

Figure S5. ^{13}C–NMR spectrum of 2-(2,3-dihydro-1,4-thiaselenin-2-ylsulfanyl)-1,3-benzothiazole (4)

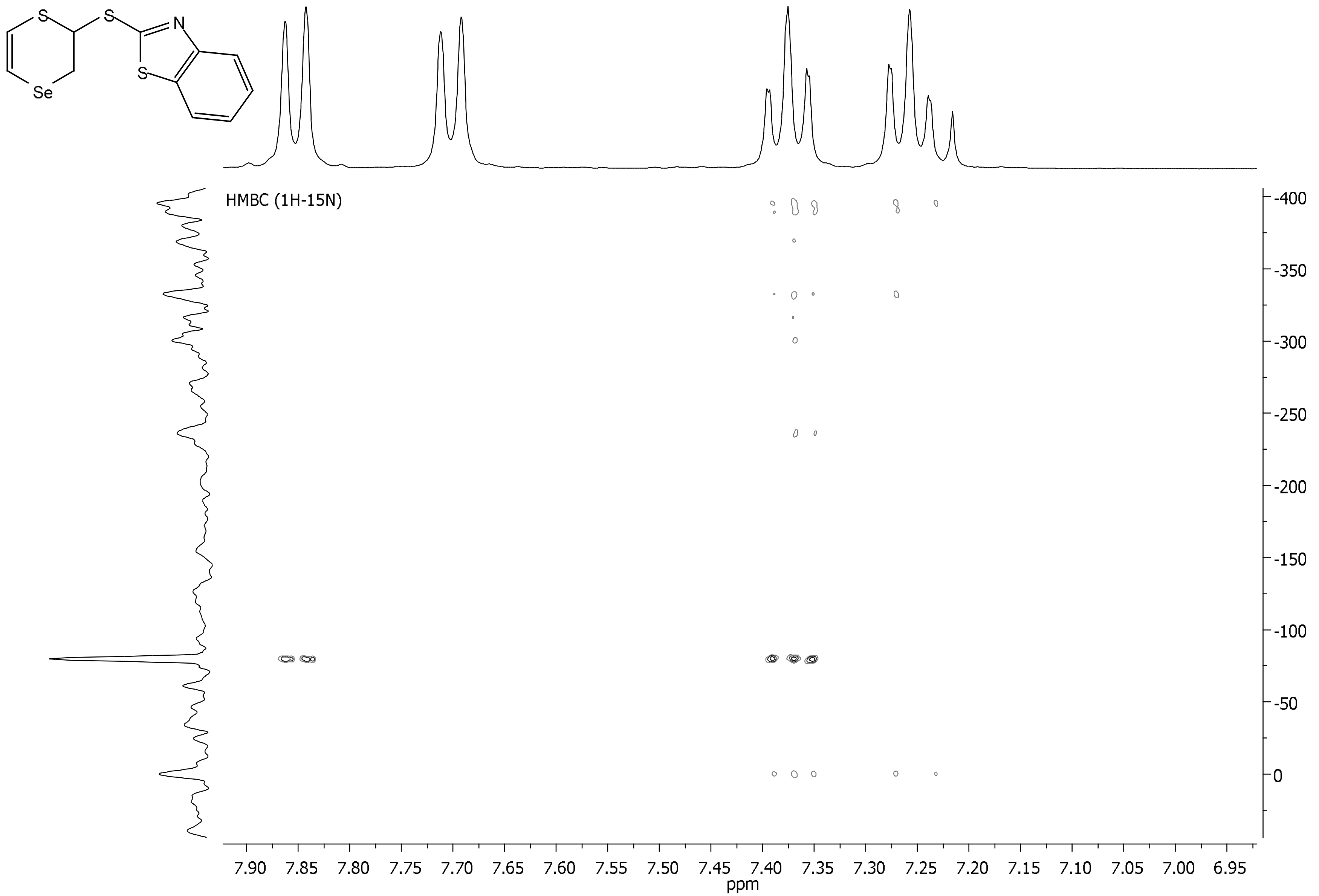

Figure S6. HMBC–(^{1}H–^{15}N)–NMR spectrum of 2-(2,3-dihydro-1,4-thiaselenin-2-ylsulfanyl)-1,3-benzothiazole (4)

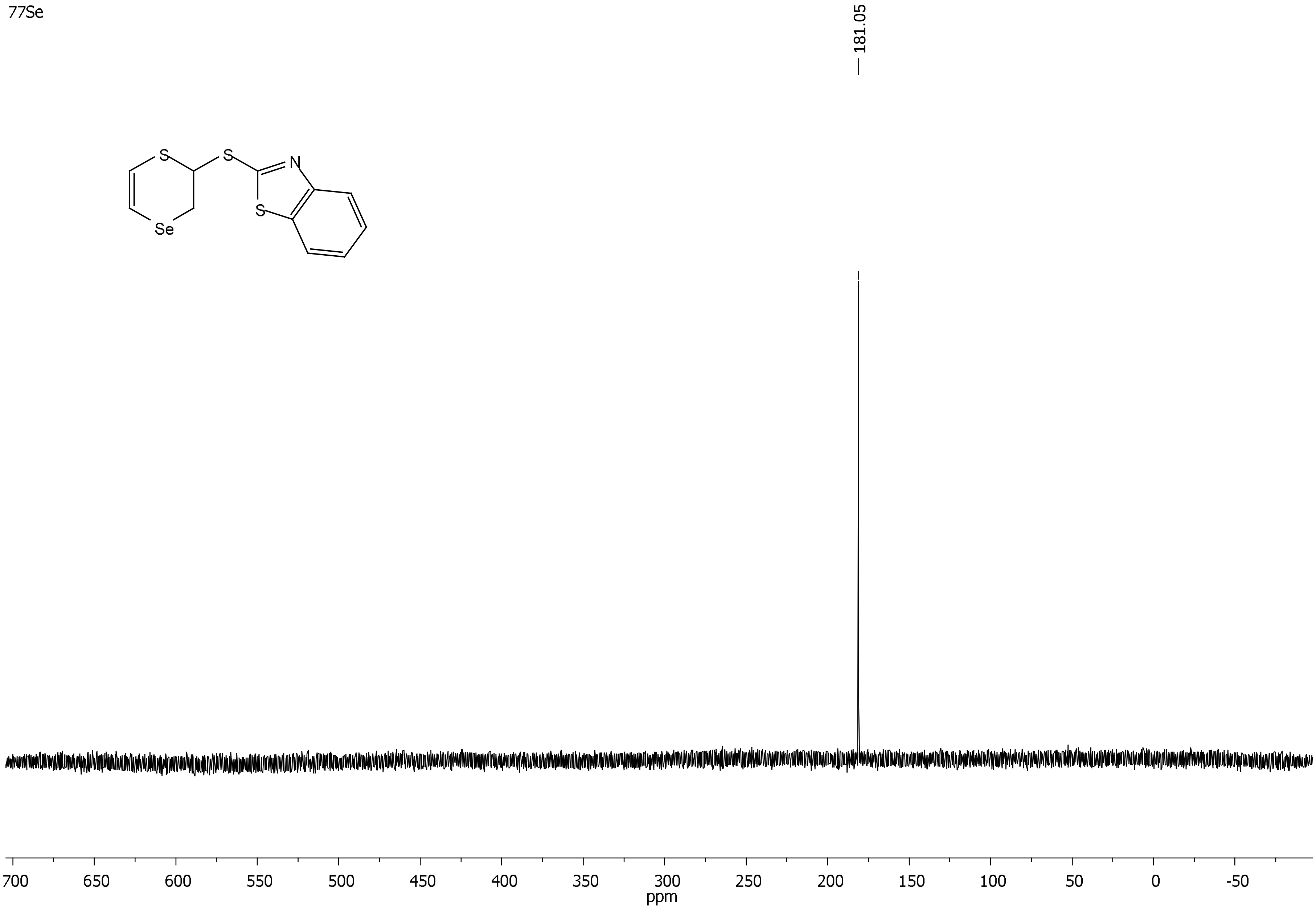

Figure S7. ^{77}Se–NMR spectrum of 2-(2,3-dihydro-1,4-thiaselenin-2-ylsulfanyl)-1,3-benzothiazole (4)

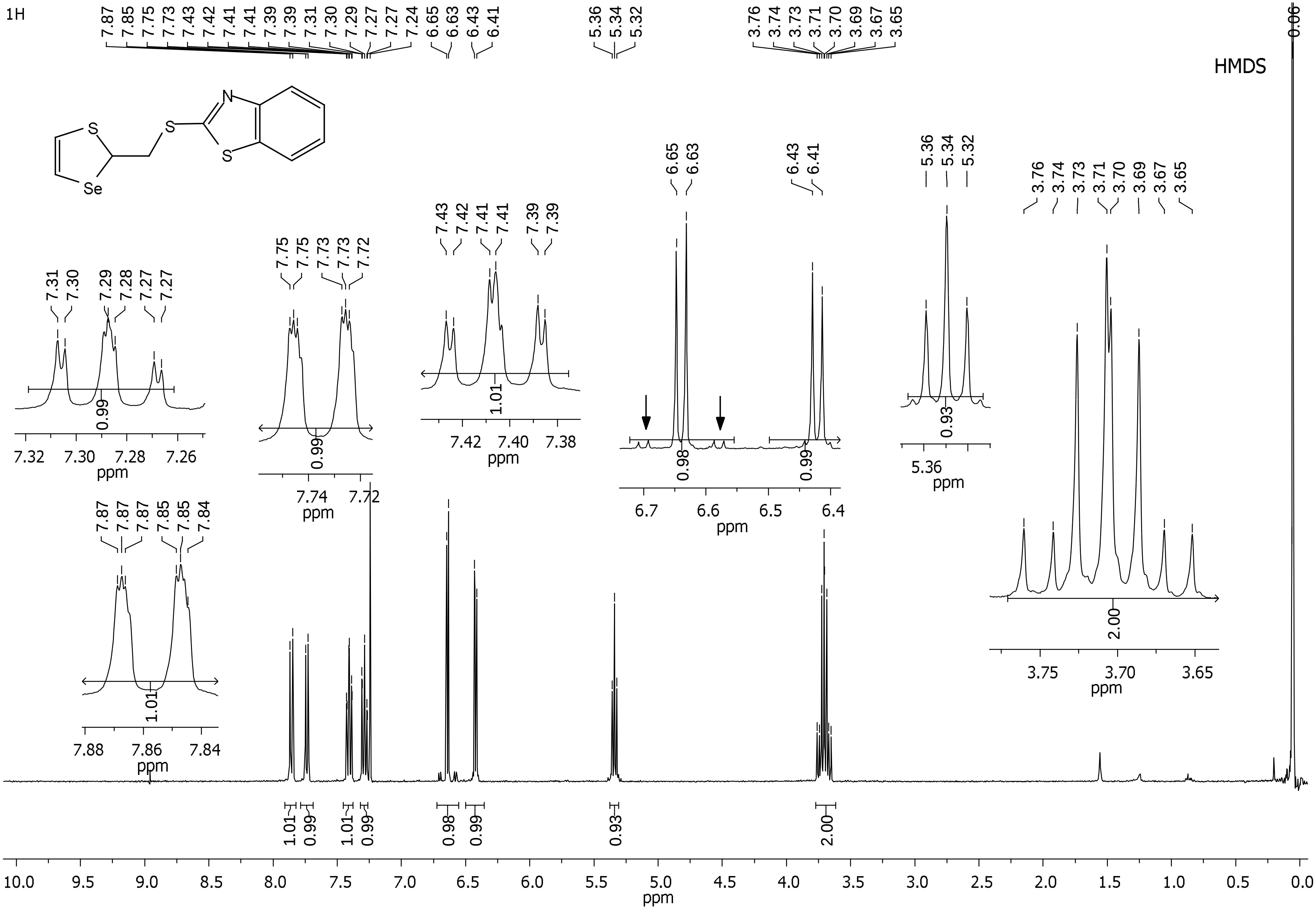

Figure S8. ^{1}H–NMR spectrum of 2-[(1,3-thiaselenol-2-ylmethyl)sulfanyl]-1,3-benzothiazole (5)

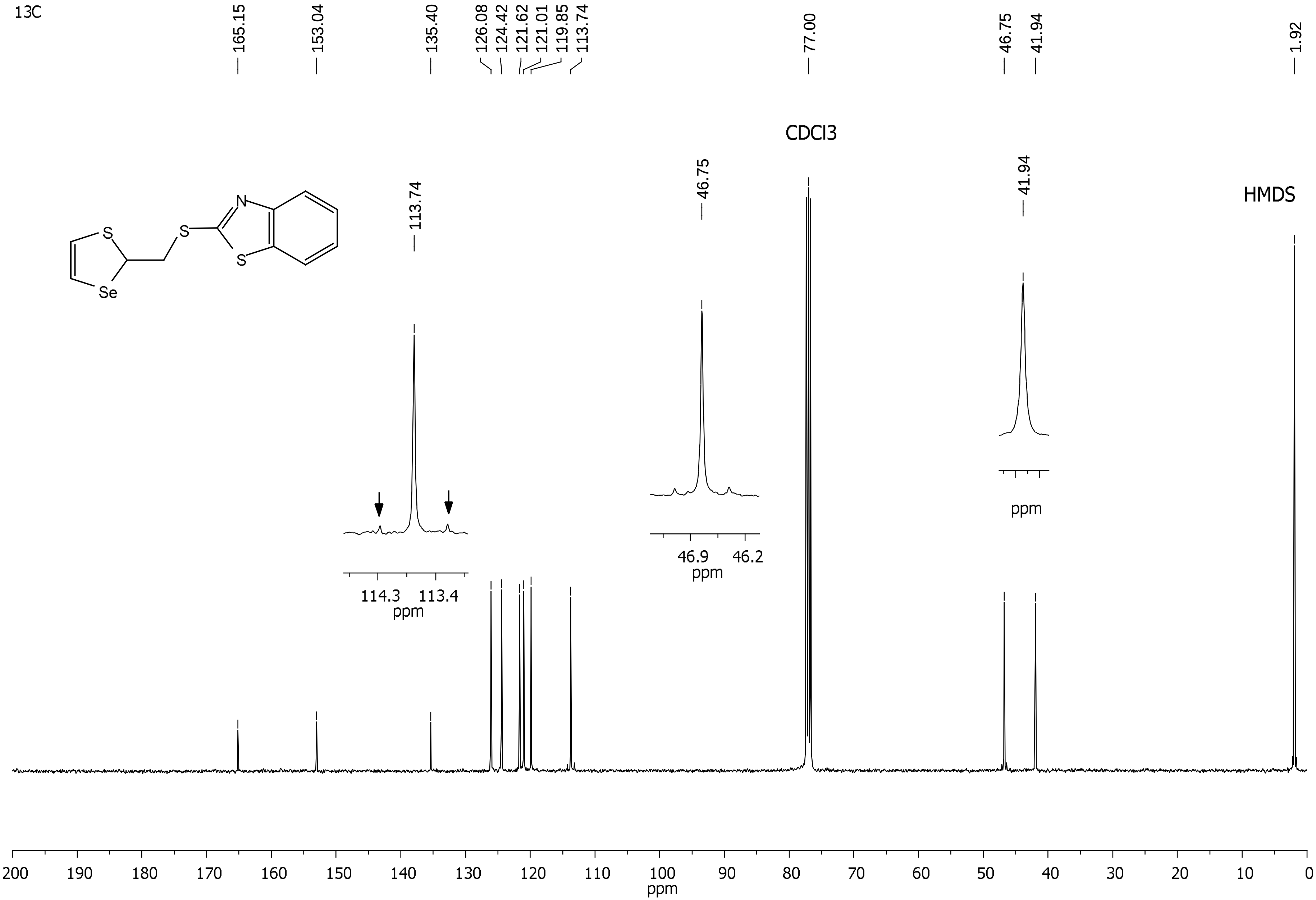

Figure S9. ^{13}C–NMR spectrum of 2-[(1,3-thiaselenol-2-ylmethyl)sulfanyl]-1,3-benzothiazole (5)

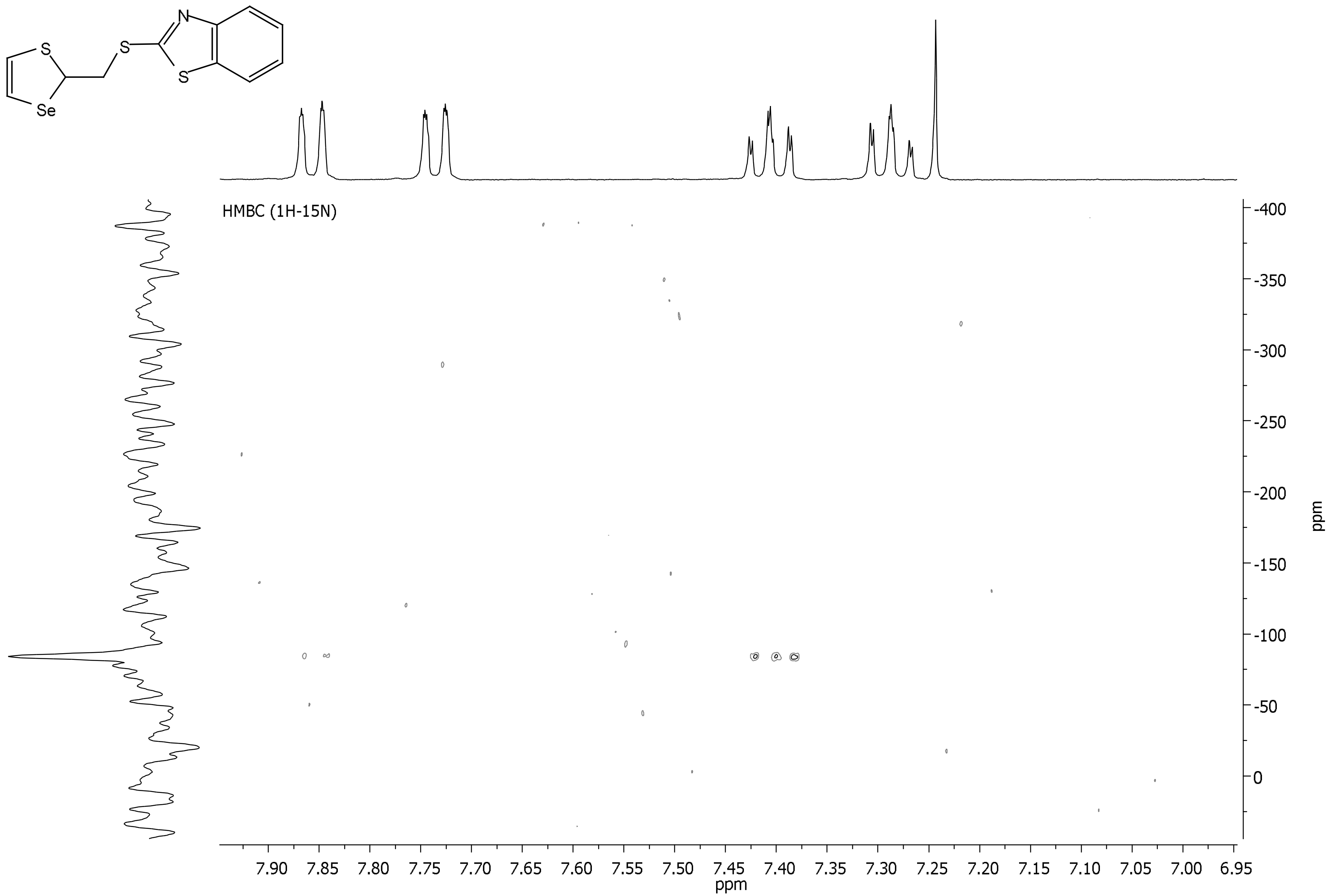

Figure S10. HMBC–(^{1}H–^{15}N)–NMR spectrum of 2-[(1,3-thiaselenol-2-ylmethyl)sulfanyl]-1,3-benzothiazole (5)

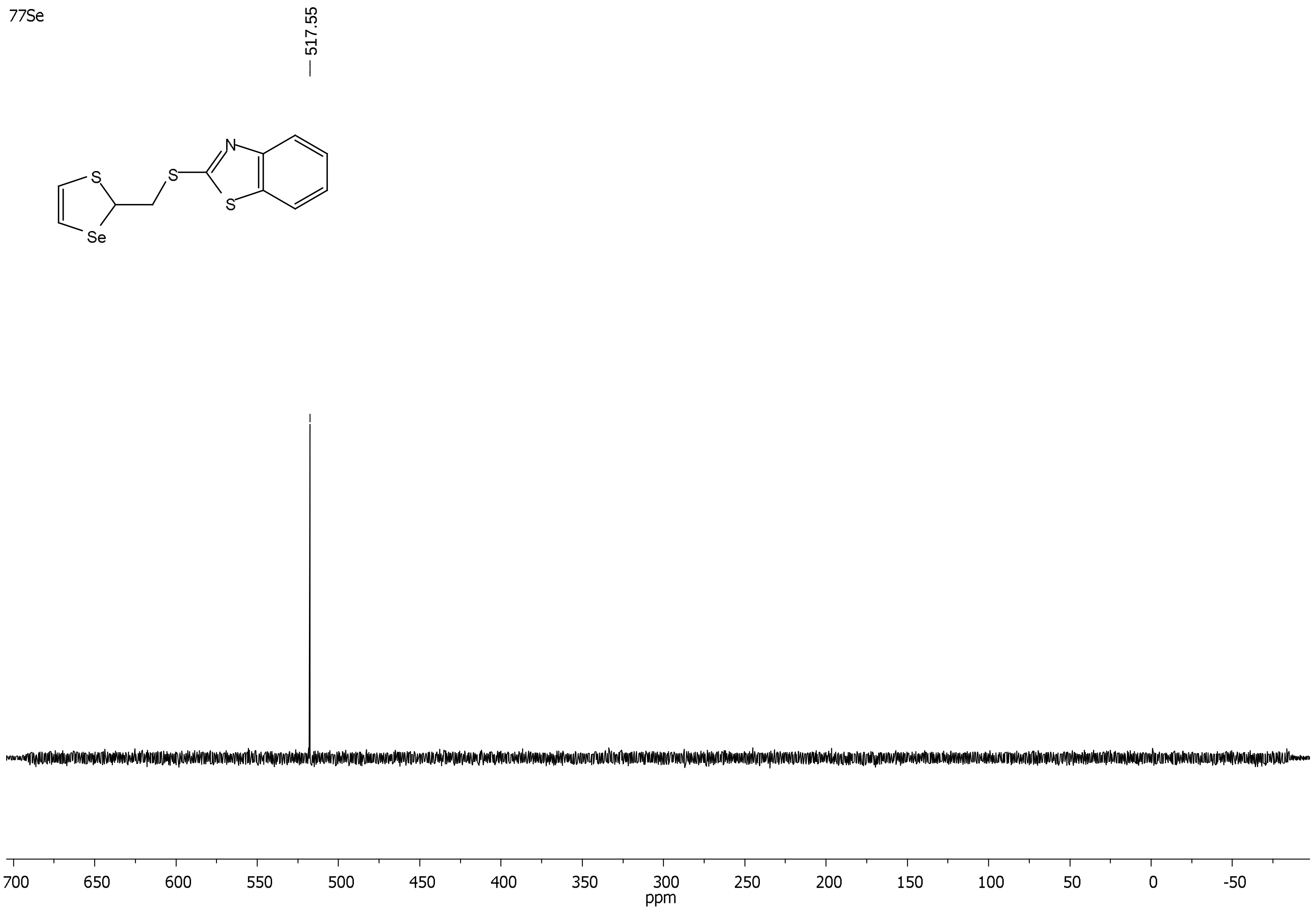

Figure S11. ^{77}Se–NMR spectrum of 2-[(1,3-thiaselenol-2-ylmethyl)sulfanyl]-1,3-benzothiazole (5)

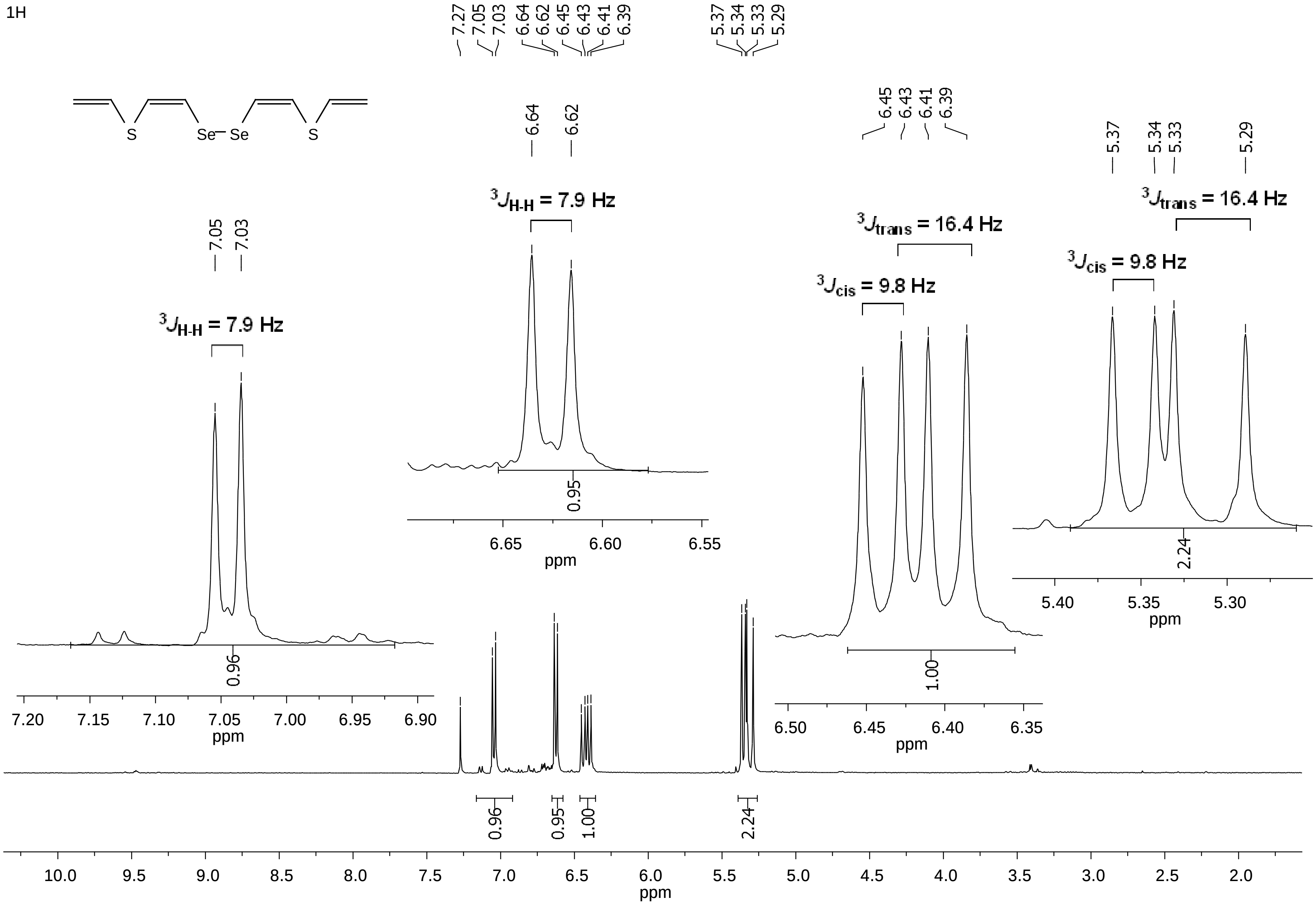

Figure S12. ^{1}H–NMR spectrum of 1,2-bis[(***Z***)-2-(vinylsulfanyl)ethenyl]diselane (6a)

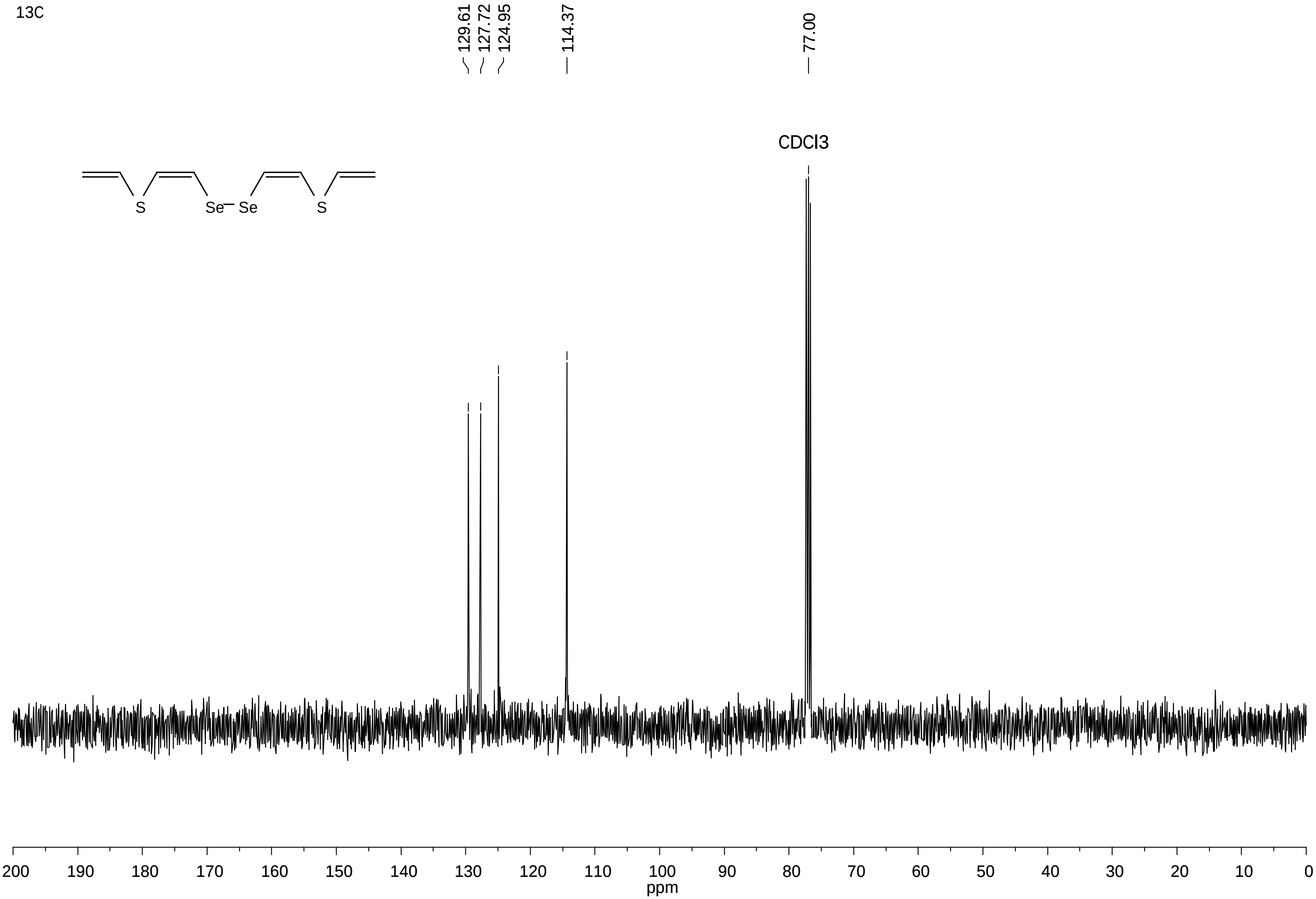

Figure S13. ^{13}C–NMR spectrum of 1,2-bis[(***Z***)-2-(vinylsulfanyl)ethenyl]diselane (6a)

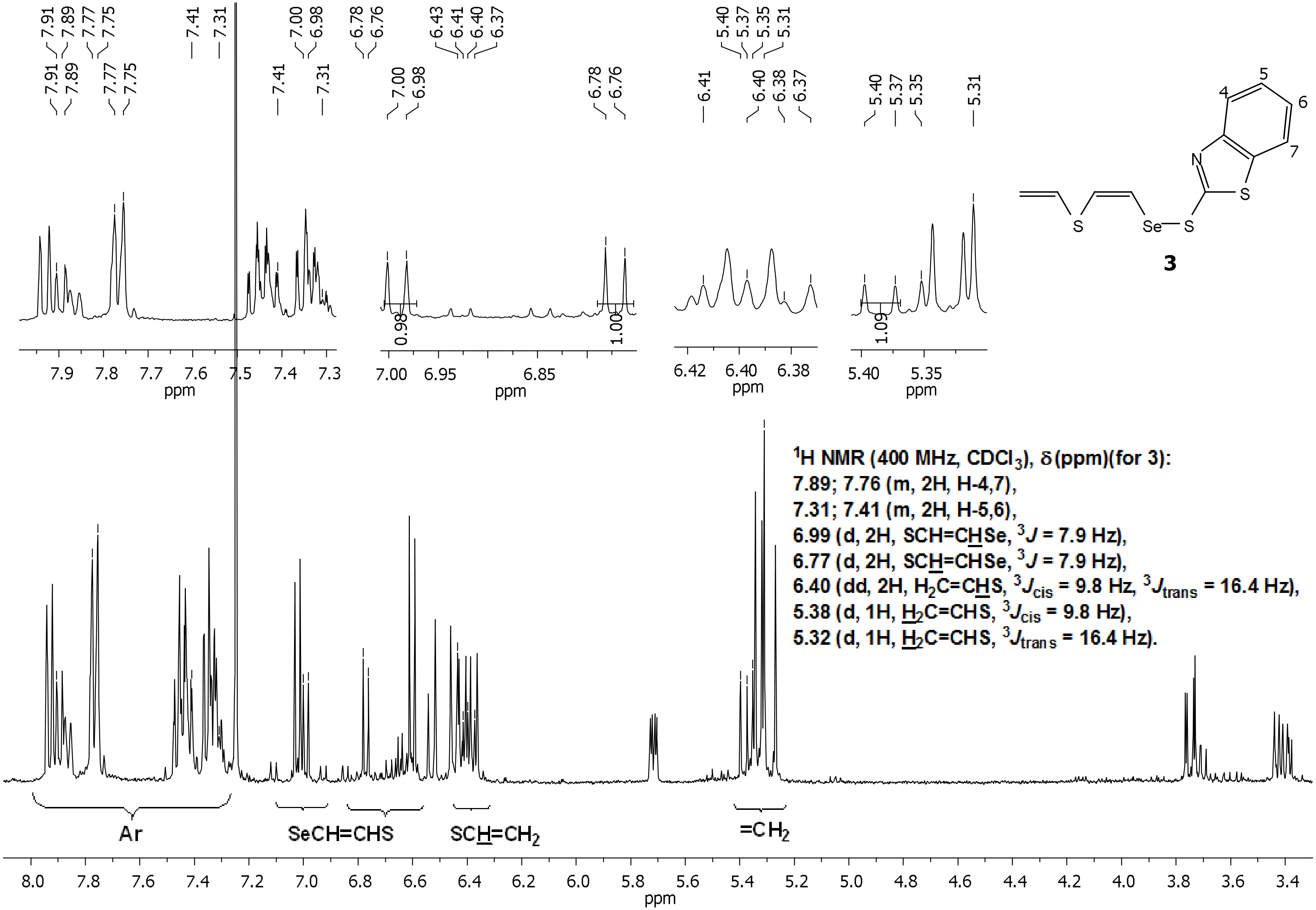

Figure S14. ^{1}H–NMR spectrum of (***Z***)-2-[(1,3-benzothiazol-2-ylsulfanyl)selanyl]ethenyl vinyl sulfide (3) in mixture (a 15 : 46 : 39 molar ratio of compounds 3, 4 and 6a) (Table 1, Run 7)

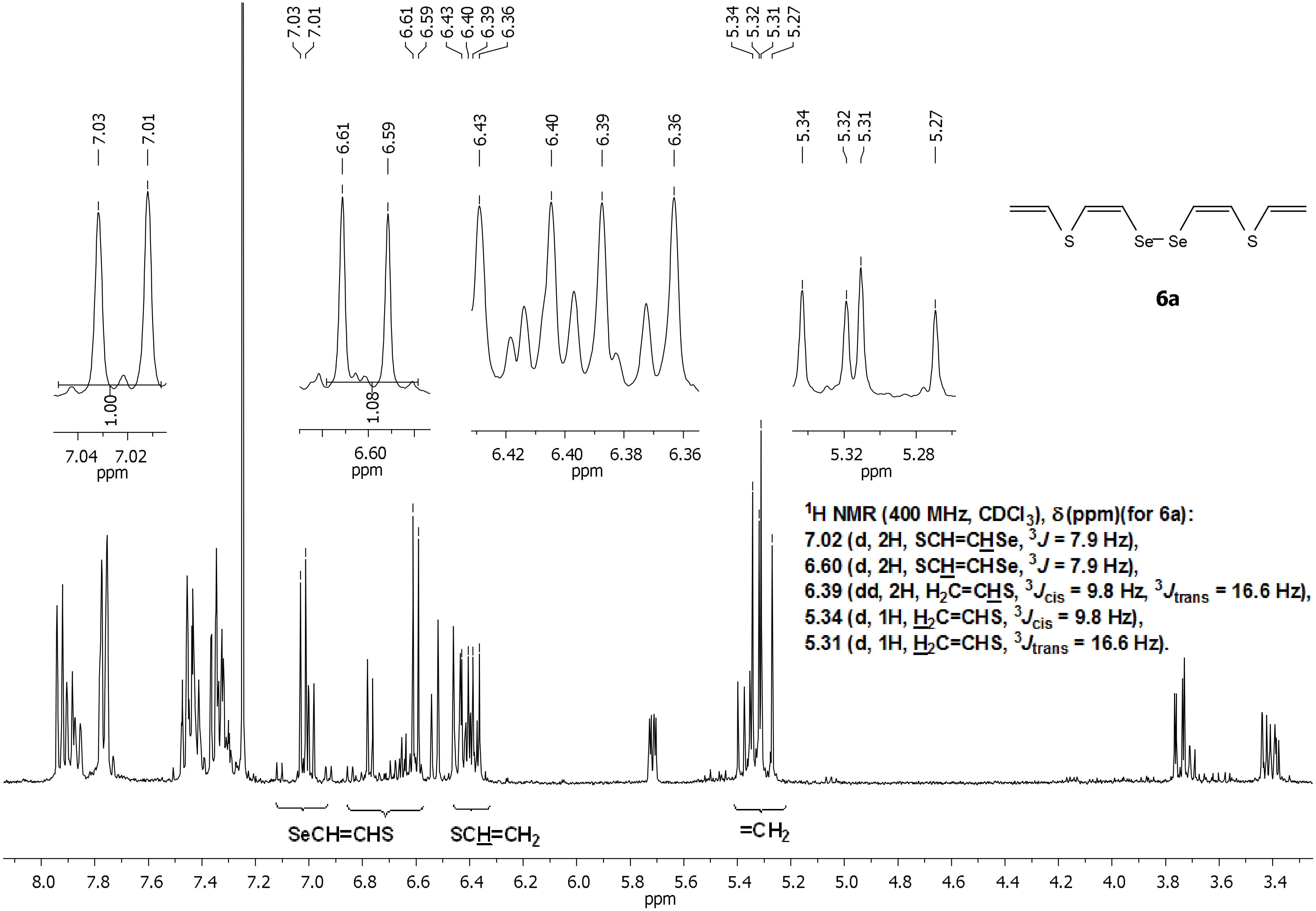

Figure S15. ^{1}H–NMR spectrum of 1,2-bis[(**Z**)-2-(vinylsulfanyl)ethenyl]diselane (6a) in mixture (a 15 : 46 : 39 molar ratio of compounds 3, 4 and 6a) (Table 1, Run 7)

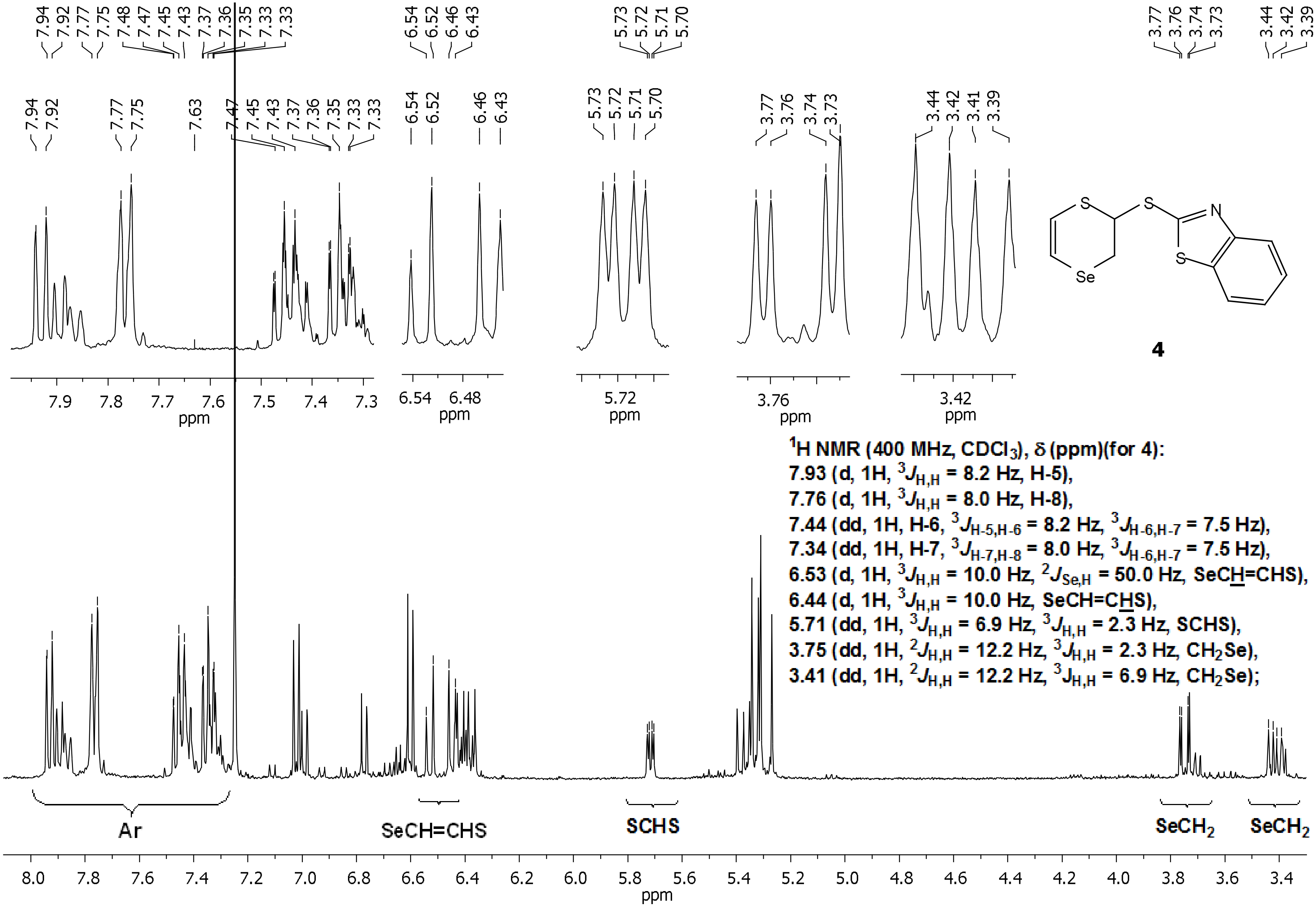

Figure S16. ^{1}H–NMR spectrum of 2-(2,3-dihydro-1,4-thiaselenin-2-ylsulfanyl)-1,3-benzothiazole (4) in mixture (a 15 : 46 : 39 molar ratio of compounds 3, 4 and 6a) (Table 1, Run 7)

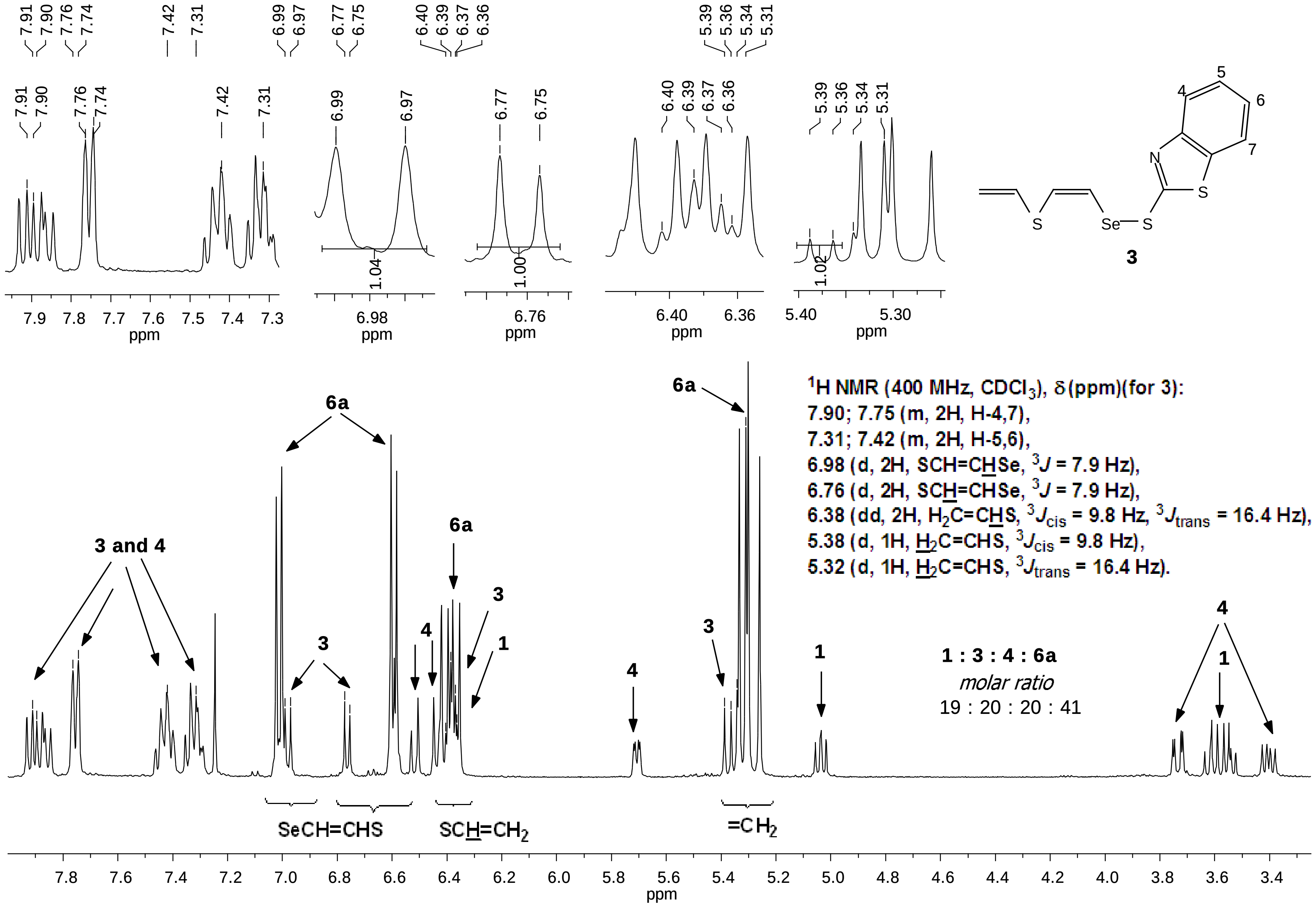

Figure S17. 1H–NMR spectrum of (***Z***)-2-[(1,3-benzothiazol-2-ylsulfanyl)selanyl]ethenyl vinyl sulfide (3) in mixture (a 19 : 20 : 20 : 41 molar ratio of compounds 1, 3, 4 and 6a) (Table 1, Run 11)

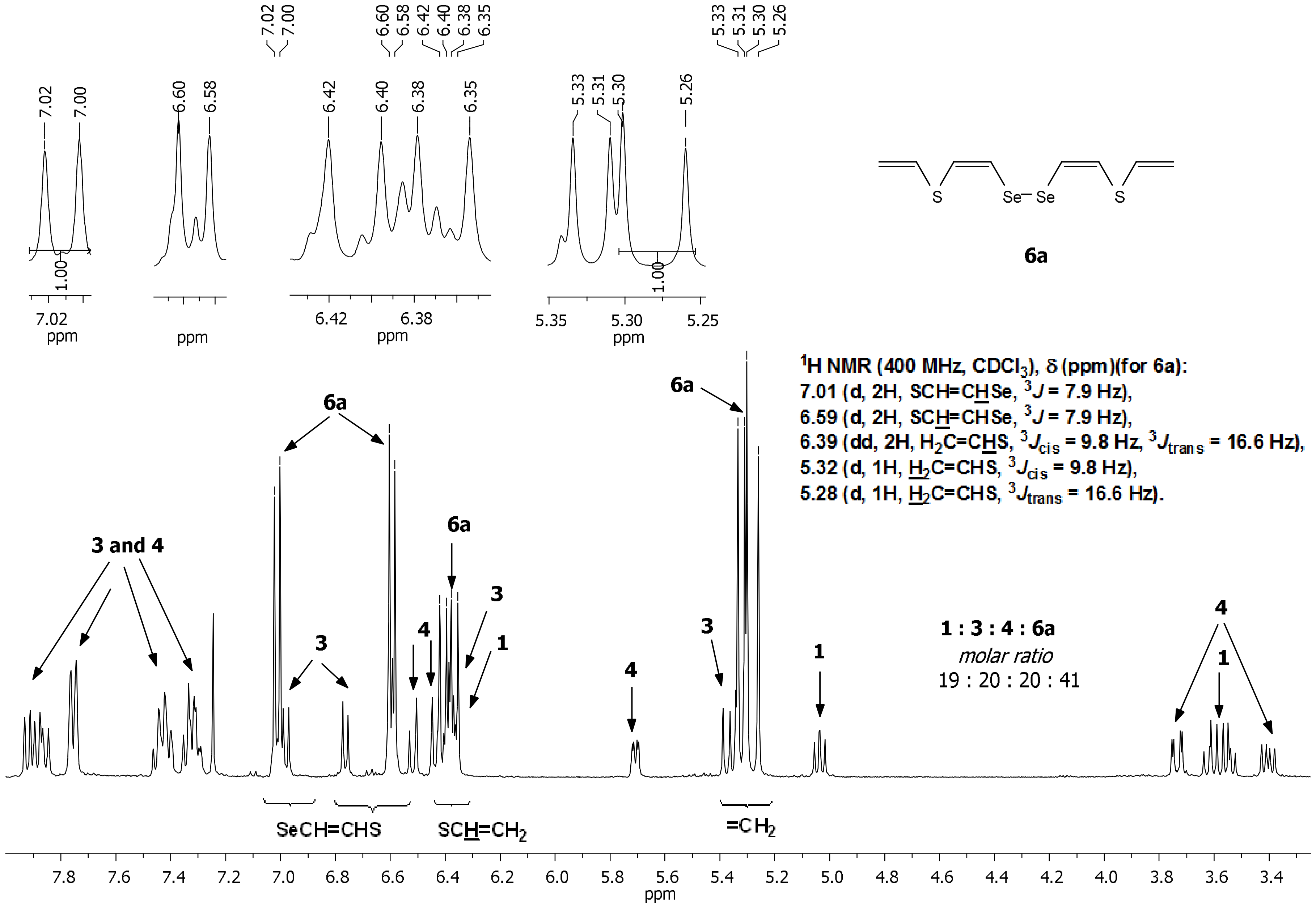

Figure S18. ^{1}H–NMR spectrum of 1,2-bis[(**Z**)-2-(vinylsulfanyl)ethenyl]diselane (6a) in mixture (a 19 : 20 : 20 : 41 molar ratio of compounds 1, 3, 4 and 6a) (Table 1, Run 11)

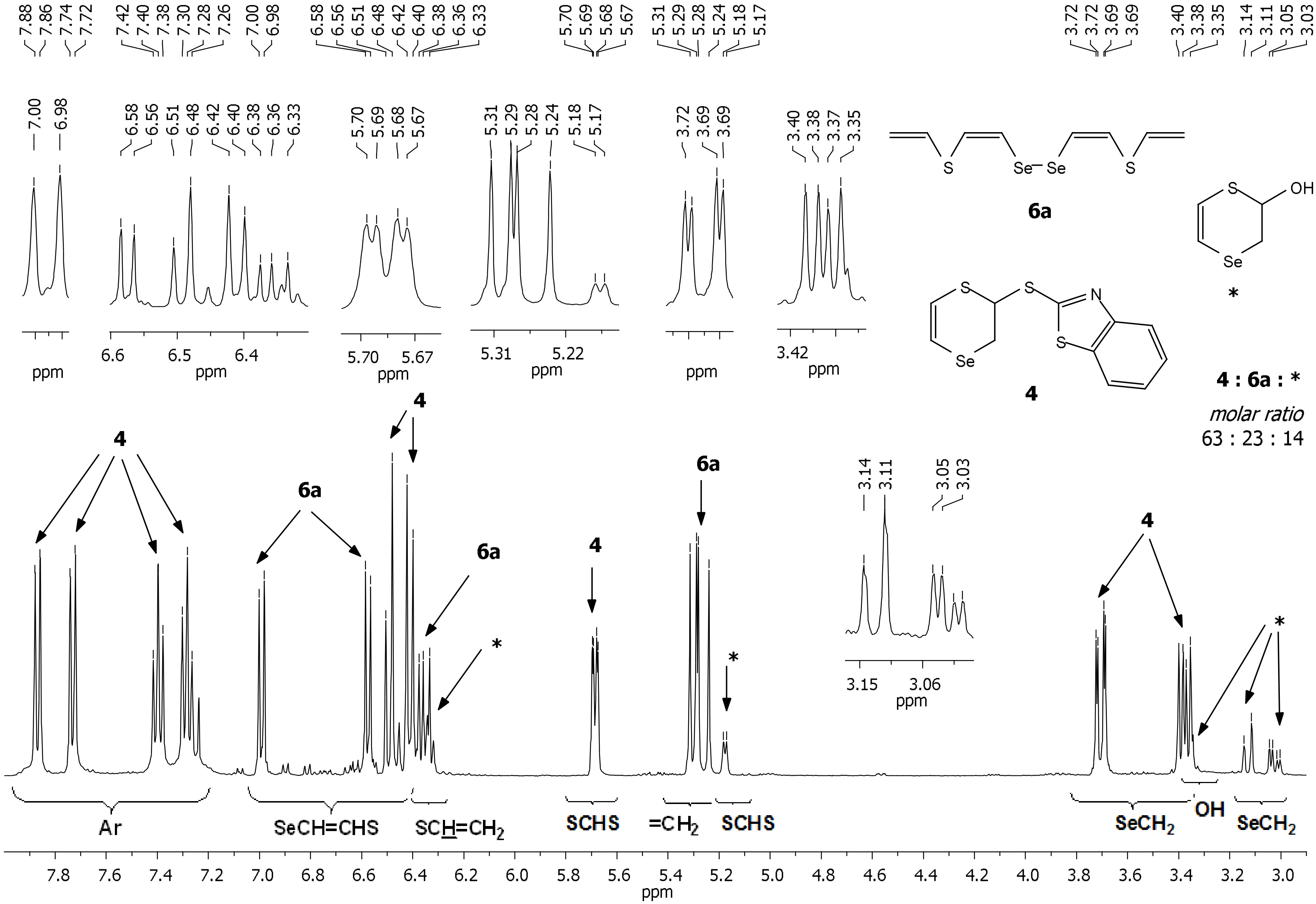

Figure S19. ^{1}H–NMR spectrum of mixture: compounds 4, 6a and 2,3-dihydro-1,4-thiaselenin-2-ol (*) (molar ratio 63 : 23 : 14) (Table 1, Run 12)

Quantum chemical calculations B3LYP/6-311+G(d,p)

Table S1. Structure 1

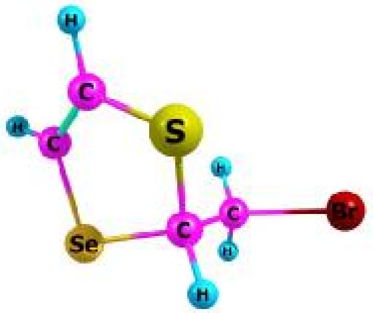

6	1.744517000	1.741861000	0.536842000
6	2.479841000	0.651274000	0.741964000
1	1.968395000	2.709654000	0.968815000
1	3.368147000	0.612543000	1.357331000
16	0.325239000	1.682836000	-0.516744000
6	0.131457000	-0.148074000	-0.518743000
34	1.926021000	-0.952601000	-0.140046000
6	-0.856495000	-0.666304000	0.509508000
1	-0.866996000	-1.754040000	0.528322000
1	-0.657395000	-0.273648000	1.503046000
35	-2.724227000	-0.138752000	0.073740000
1	-0.148654000	-0.447666000	-1.526365000

Temperature 298.150 Kelvin. Pressure 1.00000 Atm.
Zero-point correction= 0.079622 (Hartree/Particle)
Thermal correction to Energy= 0.087576
Thermal correction to Enthalpy= 0.088521
Thermal correction to Gibbs Free Energy= 0.044164
Sum of electronic and zero-point Energies= -5529.282728
Sum of electronic and thermal Energies= -5529.274774
Sum of electronic and thermal Enthalpies= -5529.273830
Sum of electronic and thermal Free Energies= -5529.318186

Table S2. Structure 2

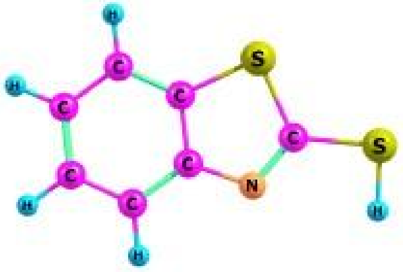

6	2.075078000	1.255771000	-0.000128000
6	0.811917000	0.668880000	-0.000053000
6	0.646033000	-0.733840000	-0.000024000
6	1.775654000	-1.559108000	-0.000047000
6	3.035202000	-0.976658000	-0.000101000
6	3.184610000	0.417404000	-0.000150000
6	-1.488639000	-0.187068000	0.000102000
7	-0.668036000	-1.177962000	0.000067000
1	2.194362000	2.332340000	-0.000167000
1	1.644239000	-2.634024000	-0.000014000
1	3.916622000	-1.607240000	-0.000118000
1	4.177935000	0.850717000	-0.000199000
16	-0.767834000	1.439754000	-0.000035000
16	-3.249706000	-0.335084000	0.000172000
1	-3.215395000	-1.683066000	0.000253000

Temperature 298.150 Kelvin. Pressure 1.00000 Atm.
Zero-point correction= 0.101134 (Hartree/Particle)
Thermal correction to Energy= 0.109357
Thermal correction to Enthalpy= 0.110301
Thermal correction to Gibbs Free Energy= 0.067535
Sum of electronic and zero-point Energies= -1120.916425
Sum of electronic and thermal Energies= -1120.908201
Sum of electronic and thermal Enthalpies= -1120.907257
Sum of electronic and thermal Free Energies= -1120.950024

Table S3. Structure TS1 [(1−2) → (1^+−2^-)·HBr]

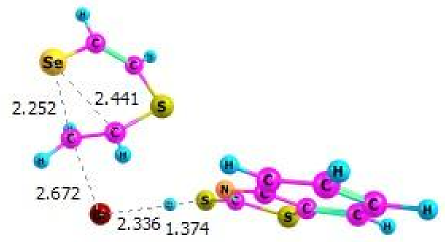

6	-2.450064000	-0.824617000	2.157440000
6	-3.557999000	-1.372449000	1.643128000
1	-2.333729000	-0.620448000	3.214811000
1	-4.397010000	-1.662394000	2.264014000
16	-0.973576000	-0.631388000	1.198908000
6	-1.688114000	-0.387276000	-0.354872000
34	-3.753767000	-1.681160000	-0.222339000
6	-2.881138000	0.353002000	-0.638423000
1	-3.146983000	0.500771000	-1.670735000
1	-3.381730000	0.938851000	0.114227000
1	-1.043160000	-0.657236000	-1.181586000
6	5.156023000	-1.401426000	-0.176905000
6	4.086061000	-0.539483000	0.055142000
6	2.779720000	-0.844445000	-0.387623000
6	2.554857000	-2.039639000	-1.080768000
6	3.619988000	-2.897541000	-1.316264000
6	4.910549000	-2.582175000	-0.868724000
6	2.313658000	1.096726000	0.570345000
7	1.807471000	0.093550000	-0.069815000
1	6.154144000	-1.159131000	0.167280000
1	1.555473000	-2.277743000	-1.424608000
1	3.453178000	-3.823591000	-1.854111000
1	5.729025000	-3.265119000	-1.064073000
16	4.068978000	1.012522000	0.870355000
16	1.451118000	2.500590000	1.182014000
35	-1.423582000	2.532325000	-1.155571000
1	0.406405000	2.427605000	0.292718000

Temperature 298.150 Kelvin. Pressure 1.00000 Atm.

Zero-point correction= 0.178889 (Hartree/Particle)
Thermal correction to Energy= 0.196344
Thermal correction to Enthalpy= 0.197288
Thermal correction to Gibbs Free Energy= 0.128592
Sum of electronic and zero-point Energies= -6650.154448
Sum of electronic and thermal Energies= -6650.136993
Sum of electronic and thermal Enthalpies= -6650.136049
Sum of electronic and thermal Free Energies= -6650.204746

Table S4. Structure (1^+−2^-)·HBr

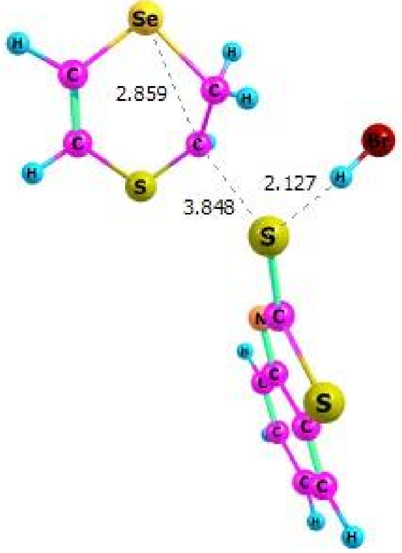

6	2.401659000	-0.814962000	-1.754583000
6	3.685931000	-0.955533000	-1.396026000
1	2.117433000	-0.615558000	-2.780414000
1	4.451482000	-0.920265000	-2.163541000
16	0.987258000	-1.176224000	-0.760342000
6	1.543027000	-1.161422000	0.785112000
34	4.368395000	-1.206089000	0.348305000
6	2.756018000	-0.442734000	1.238550000
1	2.912481000	-0.554461000	2.309135000
1	2.730121000	0.618157000	0.982720000
1	0.836025000	-1.564765000	1.499149000
6	-5.515057000	-0.513991000	0.003023000
6	-4.175533000	-0.273077000	-0.297433000
6	-3.132884000	-0.888283000	0.432700000
6	-3.452431000	-1.762187000	1.478811000
6	-4.786028000	-2.004090000	1.776708000
6	-5.810082000	-1.385360000	1.045603000
6	-1.856270000	0.278855000	-0.963027000
7	-1.849615000	-0.552427000	0.034404000
1	-6.308272000	-0.037080000	-0.560035000

1	-2.654401000	-2.232983000	2.040387000
1	-5.040783000	-2.678655000	2.586031000
1	-6.845477000	-1.586916000	1.294780000
16	-3.480568000	0.751822000	-1.538431000
16	-0.443007000	0.940000000	-1.754290000
35	0.828637000	2.958950000	0.960721000
1	0.251921000	2.170442000	-0.164260000

Temperature 298.150 Kelvin. Pressure 1.00000 Atm.
Zero-point correction= 0.178856 (Hartree/Particle)
Thermal correction to Energy= 0.197284
Thermal correction to Enthalpy= 0.198228
Thermal correction to Gibbs Free Energy= 0.125568
Sum of electronic and zero-point Energies= -6650.158098
Sum of electronic and thermal Energies= -6650.139670
Sum of electronic and thermal Enthalpies= -6650.138726
Sum of electronic and thermal Free Energies= -6650.211386

Table S5. Structure TS2 [(1^{+}–2^{-})·HBr → 3·HBr]

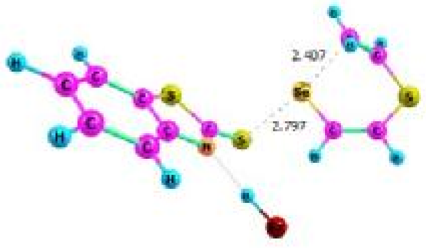

6	-3.359704000	0.460022000	1.249495000
6	-2.259499000	0.330590000	0.497246000
1	-3.463488000	1.300586000	1.925205000
1	-1.486847000	1.088210000	0.541795000
16	-4.811159000	-0.541311000	1.148284000
6	-4.164814000	-2.062828000	0.614983000
34	-1.868032000	-1.102193000	-0.701201000
6	-2.913761000	-2.549514000	0.913315000
1	-2.646097000	-3.541084000	0.570589000
1	-2.339442000	-2.141247000	1.733966000
6	4.776068000	-1.749018000	0.202726000
6	3.596217000	-1.102226000	-0.167410000
6	3.032507000	-0.086935000	0.640073000
6	3.661435000	0.281751000	1.838592000
6	4.830257000	-0.365795000	2.202542000
6	5.383481000	-1.372090000	1.392813000
6	1.502350000	-0.054750000	-0.996414000
7	1.871616000	0.470120000	0.141471000
1	5.207343000	-2.523585000	-0.419500000
1	3.228900000	1.061280000	2.453319000
1	5.328003000	-0.091462000	3.125117000
1	6.300001000	-1.861962000	1.700391000
16	2.593657000	-1.330895000	-1.580688000
16	0.123041000	0.416695000	-1.946287000
35	-0.190061000	3.267671000	-0.037866000
1	0.785106000	2.061874000	0.292502000
1	-4.845440000	-2.624437000	-0.016271000

Temperature 298.150 Kelvin. Pressure 1.00000 Atm.
Zero-point correction= 0.176895 (Hartree/Particle)
Thermal correction to Energy= 0.193225
Thermal correction to Enthalpy= 0.194169
Thermal correction to Gibbs Free Energy= 0.129299
Sum of electronic and zero-point Energies= -6650.158717
Sum of electronic and thermal Energies= -6650.142386
Sum of electronic and thermal Enthalpies= -6650.141442
Sum of electronic and thermal Free Energies= -6650.206312

Table S6. Structure 3·HBr

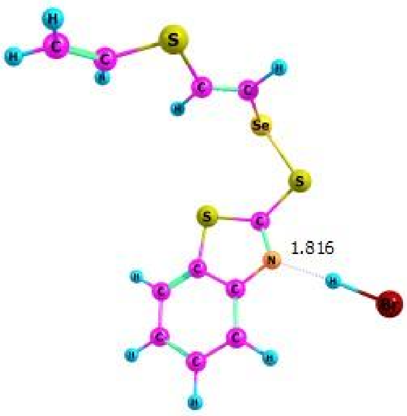

6	3.431632000	-0.321964000	0.319610000
6	2.797841000	-1.482271000	0.106335000
1	3.372539000	0.489203000	-0.398965000
1	2.816405000	-2.306251000	0.809763000
16	4.423662000	-0.005088000	1.737364000
6	4.885466000	1.686924000	1.464378000
34	1.893338000	-1.791400000	-1.551228000
6	5.252594000	2.503744000	2.448287000
1	5.232858000	2.206456000	3.490452000
1	5.595350000	3.506474000	2.223229000
6	-1.327895000	3.595813000	-1.153006000
6	-1.197265000	2.217919000	-0.985595000
6	-2.165841000	1.467050000	-0.288321000
6	-3.290515000	2.103803000	0.248168000
6	-3.421917000	3.475000000	0.077443000
6	-2.451627000	4.214579000	-0.615555000
6	-0.776077000	-0.198101000	-0.788380000
7	-1.884216000	0.109930000	-0.195078000
1	-0.581756000	4.169926000	-1.688828000
1	-4.033684000	1.525001000	0.782598000
1	-4.287712000	3.982732000	0.485789000
1	-2.577985000	5.284104000	-0.736349000
16	0.088806000	1.155003000	-1.540042000
16	-0.260615000	-1.890042000	-0.878393000
35	-3.917254000	-1.813022000	1.538848000
1	-2.972959000	-0.992596000	0.752757000
1	4.908803000	1.995876000	0.424128000

Temperature 298.150 Kelvin. Pressure 1.00000 Atm.
Zero-point correction= 0.177186 (Hartree/Particle)
Thermal correction to Energy= 0.197052
Thermal correction to Enthalpy= 0.197996
Thermal correction to Gibbs Free Energy= 0.119489
Sum of electronic and zero-point Energies= -6650.211105
Sum of electronic and thermal Energies= -6650.191239
Sum of electronic and thermal Enthalpies= -6650.190295
Sum of electronic and thermal Free Energies= -6650.268802

Table S7. Structure TS3 [$(1^{+}–2^{-})$·HBr → 4·HBr]

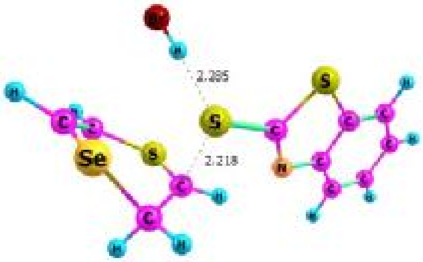

6	2.929394000	0.100919000	1.495557000
6	3.526850000	-0.287326000	0.364362000
1	3.390638000	0.836684000	2.143049000
1	4.473766000	0.164436000	0.090563000
16	1.280251000	-0.257090000	2.026391000
6	0.735961000	-1.462328000	0.983813000
34	2.893961000	-1.537955000	-0.913914000
6	1.626901000	-2.437904000	0.296760000
1	1.042884000	-3.113462000	-0.324698000
1	2.209416000	-3.009550000	1.024538000
1	-0.327923000	-1.664000000	1.088116000
6	-5.025007000	0.639076000	-0.692173000
6	-3.688481000	0.249907000	-0.610946000
6	-3.247928000	-0.682259000	0.356789000
6	-4.173267000	-1.226504000	1.256499000
6	-5.504056000	-0.838732000	1.176255000
6	-5.927963000	0.086776000	0.210794000
6	-1.279381000	-0.328652000	-0.618558000
7	-1.896166000	-0.983369000	0.322169000
1	-5.354538000	1.354525000	-1.436041000

1	-3.837662000	-1.941278000	1.998579000
1	-6.226462000	-1.255881000	1.868486000
1	-6.971194000	0.377418000	0.166248000
16	-2.319563000	0.759255000	-1.580352000
16	0.430208000	-0.473202000	-0.977846000
35	1.578518000	2.927548000	0.165380000
1	1.138950000	1.619747000	-0.395208000

Temperature 298.150 Kelvin. Pressure 1.00000 Atm.
Zero-point correction= 0.179857 (Hartree/Particle)
Thermal correction to Energy= 0.196140
Thermal correction to Enthalpy= 0.197084
Thermal correction to Gibbs Free Energy= 0.132165
Sum of electronic and zero-point Energies= -6650.150243
Sum of electronic and thermal Energies= -6650.133960
Sum of electronic and thermal Enthalpies= -6650.133016
Sum of electronic and thermal Free Energies= -6650.197935

Table S8. Structure 4·HBr

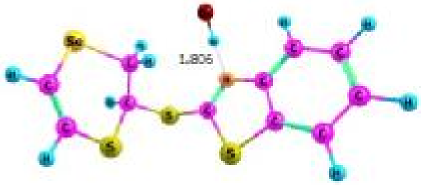

6	3.338194000	-2.177541000	-0.605703000
6	3.848065000	-1.162666000	-1.303797000
1	3.602486000	-3.197778000	-0.867549000
1	4.535550000	-1.359489000	-2.117466000
16	2.234316000	-2.187288000	0.778174000
6	1.842061000	-0.411998000	1.092438000
34	3.455654000	0.690720000	-1.066637000
6	1.717597000	0.442336000	-0.158033000
1	1.019583000	0.013054000	-0.873632000
1	1.390087000	1.449166000	0.101014000
6	-3.844233000	-2.452606000	-0.833359000
6	-2.838563000	-1.756075000	-0.159190000
6	-2.711752000	-0.353699000	-0.279422000
6	-3.605869000	0.365195000	-1.086171000
6	-4.600679000	-0.329308000	-1.751980000
6	-4.719087000	-1.724799000	-1.626276000
6	-0.996984000	-0.707606000	1.095232000
7	-1.669688000	0.194294000	0.446406000
1	-3.940296000	-3.527295000	-0.740989000
1	-3.504060000	1.439651000	-1.174802000
1	-5.300259000	0.209005000	-2.380093000
1	-5.508360000	-2.242666000	-2.158497000
16	-1.586698000	-2.357713000	0.900895000
16	0.343114000	-0.337532000	2.198808000
35	-1.136085000	3.432342000	0.365290000
1	-1.363110000	1.973847000	0.463896000
1	2.602697000	-0.000846000	1.755321000

Temperature 298.150 Kelvin. Pressure 1.00000 Atm.
Zero-point correction= 0.181038 (Hartree/Particle)
Thermal correction to Energy= 0.198876
Thermal correction to Enthalpy= 0.199820
Thermal correction to Gibbs Free Energy= 0.128436
Sum of electronic and zero-point Energies= -6650.205469
Sum of electronic and thermal Energies= -6650.187631
Sum of electronic and thermal Enthalpies= -6650.186687
Sum of electronic and thermal Free Energies= -6650.258071

Table S9. Structure 3·H⁺(N)

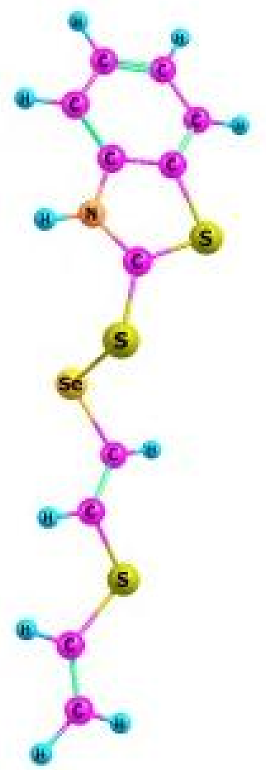

6	-5.113585000	1.337731000	-0.464070000
6	-3.915192000	0.792335000	-0.008564000
6	-3.802054000	-0.577427000	0.252102000
6	-4.876099000	-1.446352000	0.069864000
6	-6.069354000	-0.899306000	-0.383754000
6	-6.187050000	0.473441000	-0.647514000
6	-1.651255000	0.120437000	0.787917000
7	-2.514443000	-0.898098000	0.695062000
1	-5.208172000	2.396442000	-0.669700000
1	-4.784506000	-2.506657000	0.272694000
1	-6.923985000	-1.546396000	-0.536709000
1	-7.130830000	0.869287000	-1.001400000
16	-2.384811000	1.617622000	0.314822000
16	-0.020191000	-0.125222000	1.311522000
34	1.076990000	-0.774281000	-0.648783000
6	2.575748000	0.361013000	-0.700255000
1	2.460610000	1.324124000	-1.182432000
6	3.764610000	-0.076549000	-0.221445000
1	3.839107000	-1.045953000	0.262222000
16	5.236416000	0.825710000	-0.352574000
6	6.420913000	-0.295175000	0.364418000
1	6.031389000	-1.268477000	0.642094000
6	7.707487000	0.020607000	0.464649000
1	8.097971000	0.989055000	0.173864000
1	8.415546000	-0.707593000	0.841468000
1	-2.241320000	-1.845898000	0.930704000

Temperature 298.150 Kelvin. Pressure 1.00000 Atm.

Zero-point correction= 0.182219 (Hartree/Particle)

Thermal correction to Energy= 0.197713

Thermal correction to Enthalpy= 0.198657

Thermal correction to Gibbs Free Energy= 0.134818

Sum of electronic and zero-point Energies= -4075.813080

Sum of electronic and thermal Energies= -4075.797585

Sum of electronic and thermal Enthalpies= -4075.796641

Sum of electronic and thermal Free Energies= -4075.860481

Table S10. Structure 3·H^{+}(S)

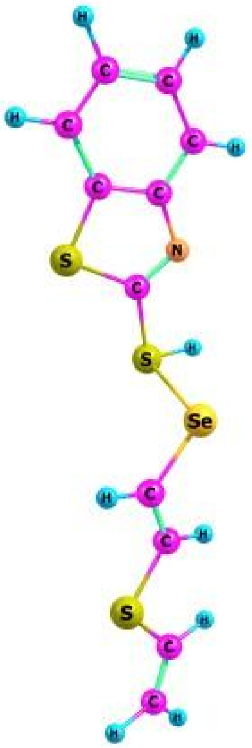

6	-5.177292000	-1.375896000	0.553213000
6	-3.985258000	-0.840582000	0.067160000
6	-3.884347000	0.521429000	-0.318509000
6	-5.004280000	1.361878000	-0.214478000
6	-6.185047000	0.829250000	0.268961000
6	-6.270059000	-0.524213000	0.648655000
6	-1.828608000	-0.094677000	-0.763061000
7	-2.641877000	0.908025000	-0.773678000
1	-5.254870000	-2.415108000	0.846224000
1	-4.922791000	2.399970000	-0.510874000
1	-7.061135000	1.459673000	0.358361000
1	-7.209552000	-0.912320000	1.023470000
16	-2.445418000	-1.640188000	-0.187483000
16	-0.147805000	0.057065000	-1.299563000
34	1.220220000	0.980992000	0.689578000
6	2.647817000	-0.180758000	0.798927000
1	2.532582000	-1.079684000	1.394343000
6	3.845574000	0.130522000	0.207409000
1	3.940237000	1.033616000	-0.388603000
16	5.245030000	-0.833246000	0.387445000
6	6.478891000	0.078089000	-0.506675000
1	6.140939000	0.995925000	-0.974349000
6	7.732616000	-0.358828000	-0.582242000
1	8.067873000	-1.275387000	-0.110375000
1	8.469179000	0.211648000	-1.134651000
1	-0.345760000	1.256374000	-1.895985000

Temperature 298.150 Kelvin. Pressure 1.00000 Atm.
Zero-point correction= 0.178645 (Hartree/Particle)
Thermal correction to Energy= 0.196176
Thermal correction to Enthalpy= 0.197121
Thermal correction to Gibbs Free Energy= 0.126667
Sum of electronic and zero-point Energies= -4075.776559
Sum of electronic and thermal Energies= -4075.759028
Sum of electronic and thermal Enthalpies= -4075.758084
Sum of electronic and thermal Free Energies= -4075.828537

Table S11. Structure TS [3·H+(N) → 3·H+(S)]

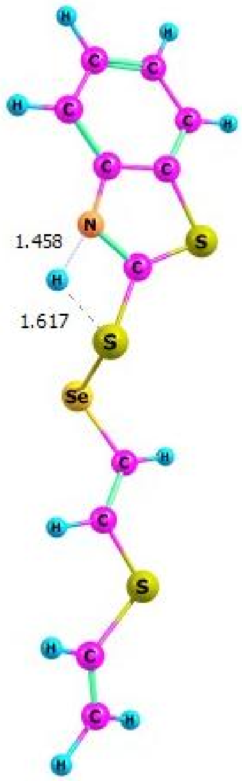

6	5.246272000	1.312375000	0.296076000
6	3.994286000	0.798376000	-0.037199000
6	3.815190000	-0.579030000	-0.314894000
6	4.901185000	-1.461047000	-0.264563000
6	6.142823000	-0.944408000	0.063489000
6	6.313236000	0.424111000	0.340662000
6	1.712853000	0.167823000	-0.579020000
7	2.498165000	-0.887341000	-0.606122000
1	5.387381000	2.364165000	0.510517000
1	4.759118000	-2.512876000	-0.478166000
1	7.000211000	-1.604397000	0.108921000
1	7.298356000	0.796171000	0.594340000
16	2.459538000	1.665974000	-0.182129000
16	0.116887000	-0.404477000	-1.089758000
34	-1.196380000	-0.687944000	0.935997000
6	-2.643037000	0.447206000	0.630740000
1	-2.534375000	1.487729000	0.912639000
6	-3.816572000	-0.054799000	0.158308000
1	-3.893770000	-1.103216000	-0.114256000
16	-5.241672000	0.894117000	-0.024135000
6	-6.419092000	-0.297642000	-0.623482000
1	-5.995694000	-1.175306000	-1.099237000
6	-7.727398000	-0.079968000	-0.540913000
1	-8.148409000	0.801122000	-0.070403000
1	-8.421786000	-0.798325000	-0.959465000
1	1.264220000	-1.541366000	-1.024818000

Temperature 298.150 Kelvin. Pressure 1.00000 Atm.
Zero-point correction= 0.175959 (Hartree/Particle)
Thermal correction to Energy= 0.192972
Thermal correction to Enthalpy= 0.193916
Thermal correction to Gibbs Free Energy= 0.125365
Sum of electronic and zero-point Energies= -4075.749163
Sum of electronic and thermal Energies= -4075.732149
Sum of electronic and thermal Enthalpies= -4075.731205
Sum of electronic and thermal Free Energies= -4075.799757

Table S12. Structure TS5

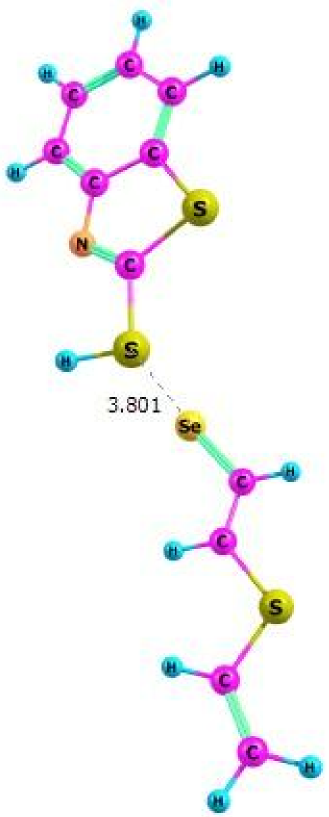

6	5.405324000	-0.344150000	-1.313267000
6	4.212254000	-0.510758000	-0.613462000
6	4.053571000	-0.025499000	0.712623000
6	5.117858000	0.639922000	1.343676000
6	6.300100000	0.805113000	0.646741000
6	6.442485000	0.318424000	-0.667990000
6	2.056546000	-0.901932000	0.454447000
7	2.820283000	-0.258733000	1.277420000
1	5.526851000	-0.715384000	-2.322896000
1	4.992750000	1.006217000	2.354772000
1	7.133021000	1.314981000	1.115057000
1	7.381634000	0.462162000	-1.188664000
16	2.734614000	-1.299509000	-1.128688000
16	0.412314000	-1.384190000	0.852736000
34	-1.601671000	1.703293000	-0.072037000
6	-2.999945000	0.812699000	-0.821212000
1	-3.032488000	0.689218000	-1.899795000
6	-4.043116000	0.339236000	-0.039740000
1	-4.003895000	0.449975000	1.040135000
16	-5.417272000	-0.391012000	-0.713813000
6	-6.437008000	-0.748085000	0.688030000
1	-6.024564000	-0.487607000	1.656054000
6	-7.635167000	-1.306403000	0.526058000
1	-8.041006000	-1.561701000	-0.446323000
1	-8.249250000	-1.523506000	1.391626000
1	0.519893000	-1.011224000	2.148147000

Temperature 298.150 Kelvin. Pressure 1.00000 Atm.
Zero-point correction= 0.177372 (Hartree/Particle)
Thermal correction to Energy= 0.194684
Thermal correction to Enthalpy= 0.195628
Thermal correction to Gibbs Free Energy= 0.122988
Sum of electronic and zero-point Energies= -4075.761363
Sum of electronic and thermal Energies= -4075.744051
Sum of electronic and thermal Enthalpies= -4075.743107
Sum of electronic and thermal Free Energies= -4075.815747

Table S13. Structure A⁺–2

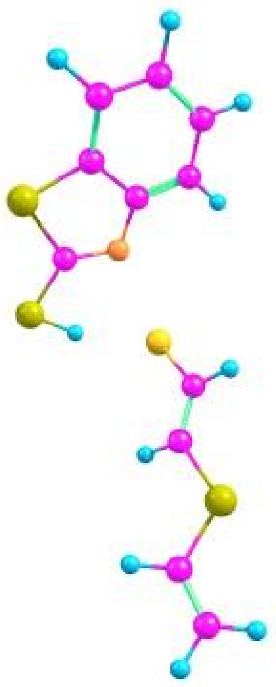

6	-4.155583000	-0.565903000	-1.371384000
6	-3.063952000	0.103779000	-0.823078000
6	-2.156217000	-0.542731000	0.026650000
6	-2.329427000	-1.888008000	0.353959000
6	-3.417333000	-2.556404000	-0.191418000
6	-4.319340000	-1.906429000	-1.045774000
6	-1.238005000	1.563543000	0.000349000
7	-1.127739000	0.318837000	0.462149000
1	-4.853918000	-0.059871000	-2.025990000
1	-1.641515000	-2.388724000	1.022448000
1	-3.572308000	-3.600045000	0.052556000
1	-5.159117000	-2.453825000	-1.455422000
16	-2.611637000	1.791510000	-1.033157000
16	-0.195520000	2.915210000	0.328765000
34	0.353576000	-0.316818000	1.649471000
6	1.534657000	-1.051108000	0.384385000
1	1.311889000	-2.051242000	0.032876000
6	2.675826000	-0.401882000	0.050173000
1	2.882457000	0.593462000	0.431158000
16	3.896879000	-1.092294000	-0.964660000
6	5.107296000	0.216334000	-1.029347000
1	4.707215000	1.222377000	-1.092568000
6	6.406690000	-0.049159000	-1.103806000
1	6.800845000	-1.057817000	-1.057361000
1	7.119320000	0.758072000	-1.225538000
1	0.574459000	2.214551000	1.201342000

Temperature 298.150 Kelvin. Pressure 1.00000 Atm.
Zero-point correction= 0.179043 (Hartree/Particle)
Thermal correction to Energy= 0.196183
Thermal correction to Enthalpy= 0.197127
Thermal correction to Gibbs Free Energy= 0.130473
Sum of electronic and zero-point Energies= -4075.791650
Sum of electronic and thermal Energies= -4075.774510
Sum of electronic and thermal Enthalpies= -4075.773566
Sum of electronic and thermal Free Energies= -4075.840220

Table S14. Structure B⁺–2

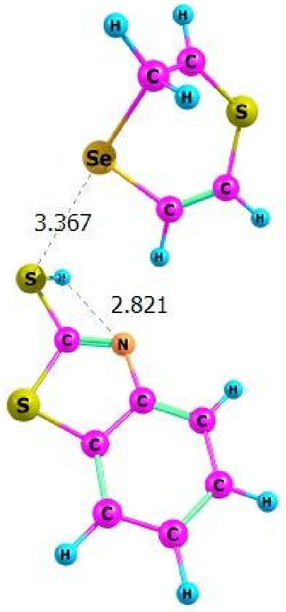

6	4.817824000	-0.474316000	-0.813290000
6	3.637959000	0.069428000	-0.303967000
6	2.668642000	-0.734428000	0.337624000
6	2.891413000	-2.110631000	0.478036000
6	4.063958000	-2.650638000	-0.025615000
6	5.016839000	-1.840870000	-0.666899000
6	1.641394000	1.212506000	0.521881000
7	1.539735000	-0.052616000	0.774018000
1	5.558955000	0.145562000	-1.302054000
1	2.157324000	-2.726949000	0.982639000
1	4.253584000	-3.712124000	0.078361000
1	5.926296000	-2.288005000	-1.049729000
16	3.105875000	1.740252000	-0.295019000
16	0.382731000	2.411322000	0.916549000
34	-2.351157000	0.942508000	-0.388076000
6	-1.619836000	-0.641722000	0.317860000
1	-0.566562000	-0.570900000	0.602108000
6	-2.304757000	-1.788329000	0.519012000
1	-1.824564000	-2.680731000	0.901897000
16	-4.051834000	-1.867137000	0.577750000
6	-4.541593000	-0.636047000	-0.437733000

1	-5.563349000	-0.301802000	-0.282469000
6	-3.704103000	-0.062078000	-1.484795000
1	-3.148992000	-0.793570000	-2.071599000
1	-4.230430000	0.652616000	-2.111692000
1	0.204126000	1.990686000	2.187822000

Temperature 298.150 Kelvin. Pressure 1.00000 Atm.
Zero-point correction= 0.180130 (Hartree/Particle)
Thermal correction to Energy= 0.196542
Thermal correction to Enthalpy= 0.197486
Thermal correction to Gibbs Free Energy= 0.131574
Sum of electronic and zero-point Energies= -4075.786028
Sum of electronic and thermal Energies= -4075.769616
Sum of electronic and thermal Enthalpies= -4075.768672
Sum of electronic and thermal Free Energies= -4075.834584

Table S15. Structure TS7

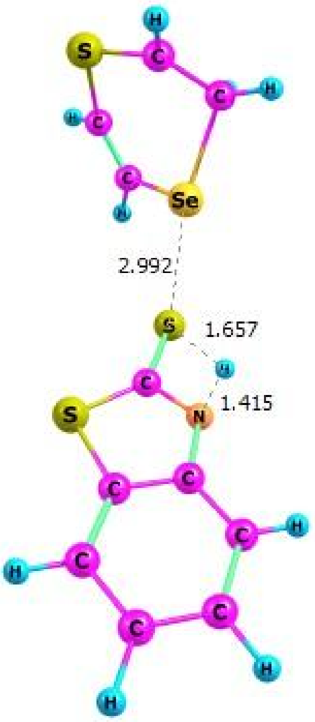

6	4.703604000	1.337182000	-0.890611000
6	3.607780000	0.806784000	-0.213926000
6	3.494764000	-0.581807000	0.028937000
6	4.493116000	-1.457812000	-0.408064000
6	5.583288000	-0.925720000	-1.079669000
6	5.688223000	0.454073000	-1.319210000
6	1.585407000	0.147366000	0.994737000
7	2.328810000	-0.903148000	0.707921000
1	4.791792000	2.400543000	-1.075227000
1	4.406470000	-2.520170000	-0.217447000
1	6.369958000	-1.585366000	-1.424850000
1	6.552651000	0.840389000	-1.845190000
16	2.210186000	1.665698000	0.457139000
16	0.212600000	-0.422225000	1.915919000
34	-2.001987000	-0.741903000	-0.069758000
6	-2.990385000	0.413917000	1.061015000
1	-2.534574000	0.595707000	2.026059000
6	-4.146818000	1.023656000	0.737297000
1	-4.676067000	1.648853000	1.446348000
16	-4.753885000	1.157853000	-0.902282000
6	-4.134408000	-0.225754000	-1.642687000
1	-4.052685000	-0.155379000	-2.723269000
6	-3.754485000	-1.420969000	-0.941824000
1	-4.381741000	-1.701394000	-0.098850000
1	-3.498749000	-2.256820000	-1.585820000
1	1.305892000	-1.566324000	1.426159000

Temperature 298.150 Kelvin. Pressure 1.00000 Atm.
Zero-point correction= 0.177249 (Hartree/Particle)
Thermal correction to Energy= 0.193240
Thermal correction to Enthalpy= 0.194184
Thermal correction to Gibbs Free Energy= 0.128640
Sum of electronic and zero-point Energies= -4075.744918
Sum of electronic and thermal Energies= -4075.728927
Sum of electronic and thermal Enthalpies= -4075.727983
Sum of electronic and thermal Free Energies= -4075.793527

Table S16. Structure 4·H+

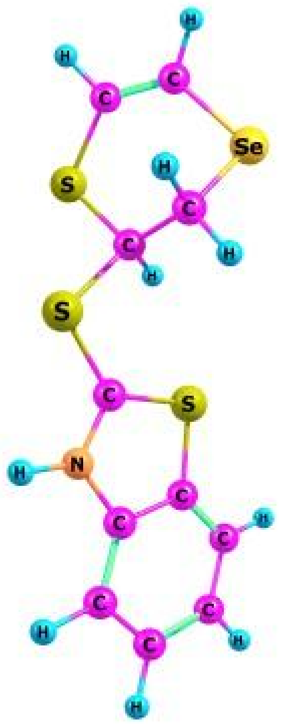

6	-4.267157000	-1.651111000	-0.637136000
6	-3.323638000	-0.685987000	-0.294059000
6	-3.708573000	0.539404000	0.260472000
6	-5.048982000	0.842092000	0.491947000
6	-5.989314000	-0.121019000	0.149804000
6	-5.604856000	-1.350018000	-0.406629000
6	-1.403719000	0.833284000	0.205928000
7	-2.597528000	1.348569000	0.522853000
1	-3.974131000	-2.600294000	-1.067268000
1	-5.346532000	1.791506000	0.920633000
1	-7.039668000	0.082775000	0.316115000
1	-6.362575000	-2.079972000	-0.662715000
16	-1.565418000	-0.759796000	-0.457667000
16	0.051720000	1.736791000	0.439161000
34	3.345776000	-1.519455000	0.259075000
6	4.580488000	-0.101026000	-0.063829000
1	5.618401000	-0.342191000	0.130493000
6	4.228308000	1.131533000	-0.428047000
1	4.972920000	1.915699000	-0.523707000
16	2.639159000	1.758607000	-0.893627000
6	1.416851000	0.572065000	-0.192355000
1	0.975211000	0.001833000	-1.008308000
6	1.921910000	-0.321605000	0.923184000
1	2.325310000	0.255760000	1.753761000
1	1.129973000	-0.975805000	1.288962000
1	-2.687874000	2.276882000	0.921956000

Temperature 298.150 Kelvin. Pressure 1.00000 Atm.
Zero-point correction= 0.185500 (Hartree/Particle)
Thermal correction to Energy= 0.200878
Thermal correction to Enthalpy= 0.201823
Thermal correction to Gibbs Free Energy= 0.139262
Sum of electronic and zero-point Energies= -4075.816181
Sum of electronic and thermal Energies= -4075.800802
Sum of electronic and thermal Enthalpies= -4075.799858
Sum of electronic and thermal Free Energies= -4075.862418

Table S17. Structure 4

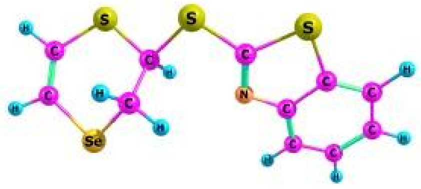

6	5.073492000	-0.036724000	-0.064657000
6	3.711369000	-0.327754000	-0.084099000
6	2.740478000	0.653613000	0.211486000
6	3.149582000	1.951594000	0.535124000
6	4.506148000	2.243359000	0.556398000
6	5.459739000	1.259327000	0.259374000
6	1.356230000	-1.025044000	-0.172555000
7	1.422891000	0.222137000	0.145488000
1	5.813018000	-0.794588000	-0.292491000
1	2.402138000	2.701529000	0.762860000
1	4.834239000	3.245522000	0.806198000
1	6.513976000	1.509245000	0.282501000
16	2.910431000	-1.850862000	-0.443843000
16	-0.129153000	-1.961829000	-0.354875000
6	-1.366123000	-0.695896000	0.216219000
6	-1.739548000	0.252514000	-0.908928000
6	-4.221032000	-0.787592000	0.575340000
6	-4.383675000	0.478597000	0.189388000
1	-2.202008000	-0.270176000	-1.744436000
1	-5.090317000	-1.404718000	0.782244000
1	-5.380396000	0.891318000	0.092437000

	34 -2.979006000 1.670686000 -0.308285000<br>16 -2.752742000 -1.728083000 0.867909000<br>1 -0.846000000 0.776691000 -1.244590000<br>1 -0.895210000 -0.156671000 1.032963000<br>Temperature 298.150 Kelvin. Pressure 1.00000 Atm.<br>Zero-point correction= 0.173233 (Hartree/Particle)<br>Thermal correction to Energy= 0.188271<br>Thermal correction to Enthalpy= 0.189216<br>Thermal correction to Gibbs Free Energy= 0.127406<br>Sum of electronic and zero-point Energies= -4075.455392<br>Sum of electronic and thermal Energies= -4075.440353<br>Sum of electronic and thermal Enthalpies= -4075.439409<br>Sum of electronic and thermal Free Energies= -4075.501218

Table S18. Structure TS8 (4 → 5)

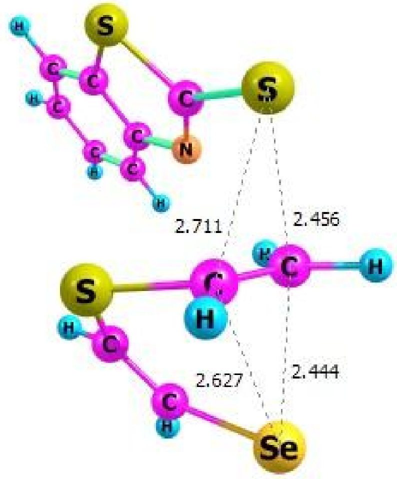	6 -1.388303000 -1.509306000 -1.076609000<br>6 -2.434815000 -1.860057000 -0.315846000<br>1 -0.639453000 -2.215925000 -1.410984000<br>1 -2.576356000 -2.900121000 -0.043249000<br>16 -1.207985000 0.111120000 -1.760067000<br>6 -1.974743000 1.086837000 -0.560623000<br>34 -3.716716000 -0.647345000 0.366594000<br>6 -1.891375000 0.905664000 0.846762000<br>1 -1.238692000 0.163067000 1.281245000<br>1 -2.452999000 1.559079000 1.493357000<br>6 4.466849000 -0.404448000 -0.581795000<br>6 3.265309000 0.173403000 -0.172971000<br>6 2.346722000 -0.532601000 0.641357000<br>6 2.647352000 -1.842766000 1.039380000<br>6 3.839917000 -2.417633000 0.627048000<br>6 4.743073000 -1.705136000 -0.177204000<br>6 1.193101000 1.360000000 0.489600000<br>7 1.206050000 0.161047000 0.992523000<br>1 5.167830000 0.142622000 -1.200606000<br>1 1.945495000 -2.377026000 1.668187000<br>1 4.082196000 -3.428800000 0.933231000<br>1 5.670416000 -2.173275000 -0.486486000<br>16 2.619505000 1.763147000 -0.516609000<br>16 -0.063989000 2.539781000 0.698734000<br>1 -2.471443000 1.964268000 -0.954095000<br>Temperature 298.150 Kelvin. Pressure 1.00000 Atm.<br>Zero-point correction= 0.169995 (Hartree/Particle)<br>Thermal correction to Energy= 0.185440<br>Thermal correction to Enthalpy= 0.186384<br>Thermal correction to Gibbs Free Energy= 0.123798<br>Sum of electronic and zero-point Energies= -4075.394499<br>Sum of electronic and thermal Energies= -4075.379054<br>Sum of electronic and thermal Enthalpies= -4075.378109<br>Sum of electronic and thermal Free Energies= -4075.440696

Table S19. Structure 5

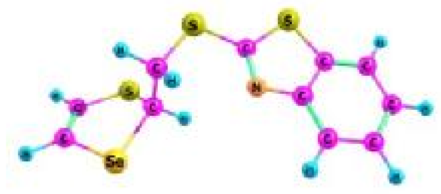

6	-4.360887000	0.706947000	-0.643953000
6	-4.499846000	-0.433660000	0.029210000
1	-5.177148000	1.385429000	-0.859850000
1	-5.434716000	-0.799083000	0.431948000
16	-2.777419000	1.175082000	-1.283688000
6	-1.777389000	0.052493000	-0.220451000
34	-2.933306000	-1.502958000	0.272805000
6	-1.247886000	0.722319000	1.043915000
1	-2.052108000	1.208387000	1.596555000
1	-0.760661000	-0.012036000	1.684595000
6	5.009226000	-0.002638000	-0.427198000
6	3.686738000	0.316669000	-0.128426000
6	2.714351000	-0.683675000	0.088594000
6	3.082778000	-2.030592000	0.004583000
6	4.400173000	-2.351109000	-0.292157000
6	5.355127000	-1.347392000	-0.506641000
6	1.403571000	1.070910000	0.387882000
7	1.438223000	-0.218356000	0.373711000
1	5.749581000	0.770348000	-0.593385000
1	2.335375000	-2.796120000	0.173155000
1	4.695824000	-3.391578000	-0.359114000
1	6.378180000	-1.620215000	-0.737657000
16	2.941470000	1.899642000	0.042076000
16	-0.020521000	2.056721000	0.715436000
1	-0.961700000	-0.340823000	-0.820919000

Temperature 298.150 Kelvin. Pressure 1.00000 Atm.
Zero-point correction= 0.173009 (Hartree/Particle)
Thermal correction to Energy= 0.188033
Thermal correction to Enthalpy= 0.188977
Thermal correction to Gibbs Free Energy= 0.127253
Sum of electronic and zero-point Energies= -4075.460298
Sum of electronic and thermal Energies= -4075.445275
Sum of electronic and thermal Enthalpies= -4075.444331
Sum of electronic and thermal Free Energies= -4075.506055

Table S20. Structure 3–3

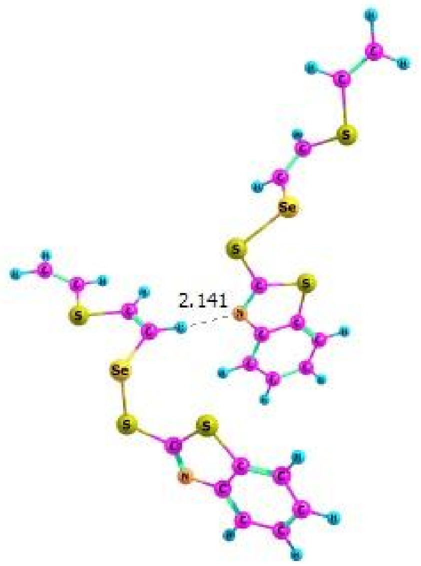

6	-0.634693000	-3.279040000	2.832531000
6	-0.892181000	-2.209961000	1.972851000
6	0.087555000	-1.222851000	1.714542000
6	1.344682000	-1.308060000	2.327281000
6	1.599028000	-2.373412000	3.183112000
6	0.619840000	-3.348548000	3.434266000
6	-1.521018000	-0.412003000	0.432217000
7	-0.310833000	-0.220030000	0.836453000
1	-1.387630000	-4.035670000	3.027610000
1	2.094587000	-0.551836000	2.122010000
1	2.570101000	-2.455039000	3.660906000
1	0.842568000	-4.171981000	4.105780000
16	-2.357401000	-1.850154000	1.066285000
16	-2.286062000	0.736974000	-0.695850000
34	-4.269345000	-0.212943000	-1.019553000
6	-5.232586000	0.645340000	0.400712000
1	-4.649801000	1.310716000	1.029410000
6	-6.534375000	0.422669000	0.599169000
1	-7.076324000	0.924118000	1.396445000
16	-7.408772000	-0.807768000	-0.338768000
6	-8.803024000	0.124681000	-0.937470000
1	-8.583271000	1.145806000	-1.236974000

6	-10.012602000	-0.415062000	-1.090966000
1	-10.233363000	-1.435439000	-0.792991000
1	-10.817455000	0.158693000	-1.539564000
6	3.471914000	-3.931649000	-1.559402000
6	3.905035000	-2.659245000	-1.183087000
6	5.096782000	-2.478464000	-0.439797000
6	5.861888000	-3.593076000	-0.071272000
6	5.428899000	-4.859702000	-0.447165000
6	4.245202000	-5.028344000	-1.183983000
6	4.518302000	-0.356514000	-0.611744000
7	5.406892000	-1.159998000	-0.134107000
1	2.558014000	-4.066158000	-2.129008000
1	6.774649000	-3.446302000	0.496367000
1	6.013684000	-5.731192000	-0.168749000
1	3.926003000	-6.026849000	-1.467007000
16	3.161439000	-1.098294000	-1.505247000
16	4.675649000	1.398312000	-0.368067000
34	2.879813000	2.187986000	-1.420310000
6	1.607161000	2.232066000	0.007727000
1	1.005488000	1.342210000	0.194893000
6	1.423000000	3.350025000	0.725017000
1	0.660771000	3.369766000	1.502408000
16	2.347772000	4.839245000	0.544204000
6	1.607248000	5.843763000	1.807796000
1	1.138790000	5.299866000	2.624741000
6	1.679203000	7.175136000	1.809186000
1	2.145549000	7.733269000	1.003145000
1	1.275743000	7.745213000	2.639441000

Temperature 298.150 Kelvin. Pressure 1.00000 Atm.
Zero-point correction= 0.342676 (Hartree/Particle)
Thermal correction to Energy= 0.377229
Thermal correction to Enthalpy= 0.378174
Thermal correction to Gibbs Free Energy= 0.262011
Sum of electronic and zero-point Energies= -8146.212625
Sum of electronic and thermal Energies= -8146.178072
Sum of electronic and thermal Enthalpies= -8146.177127
Sum of electronic and thermal Free Energies= -8146.293290

Table S21. Structure TS9

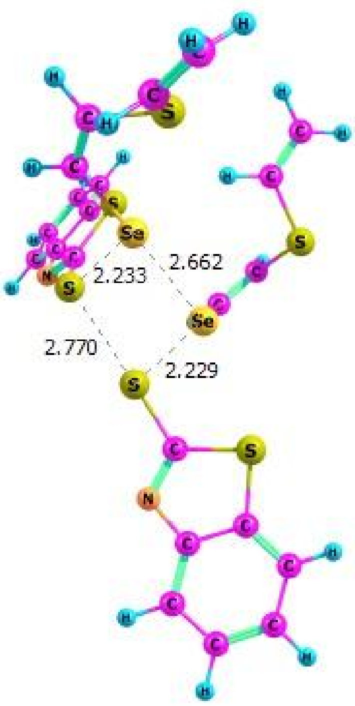

6	6.954833000	-0.470251000	1.009720000
6	5.755714000	-0.172740000	0.359779000
6	5.739571000	0.536862000	-0.866017000
6	6.946237000	0.956139000	-1.442148000
6	8.138999000	0.662998000	-0.791414000
6	8.143999000	-0.043079000	0.423018000
6	3.559933000	0.253031000	-0.654055000
7	4.478670000	0.755627000	-1.405219000
1	6.963466000	-1.016763000	1.947217000
1	6.925570000	1.499461000	-2.380796000
1	9.080552000	0.983325000	-1.226822000
1	9.087776000	-0.260765000	0.913718000
16	4.109631000	-0.572094000	0.831410000
16	1.831522000	0.488338000	-1.094644000
34	0.866151000	-0.869660000	0.386083000
6	0.492499000	0.432982000	1.727804000
1	0.736362000	1.461817000	1.481922000
6	-0.069732000	0.056562000	2.878166000
1	-0.337418000	0.775485000	3.647058000
16	-0.362697000	-1.675568000	3.215987000
6	-2.099591000	-1.827654000	2.814748000
1	-2.368785000	-1.459541000	1.828640000
6	-2.962008000	-2.452450000	3.614981000
1	-2.676395000	-2.827440000	4.592983000
1	-3.986255000	-2.617437000	3.293767000
6	-4.022964000	4.160810000	1.232620000
6	-3.164170000	3.463753000	0.381031000
6	-2.371520000	4.139469000	-0.578971000
6	-2.444365000	5.535010000	-0.682744000
6	-3.300857000	6.227320000	0.165418000
6	-4.083095000	5.547642000	1.113792000
6	-1.713550000	2.083907000	-1.031728000
7	-1.565323000	3.321636000	-1.359341000
1	-4.629182000	3.639648000	1.966469000
1	-1.833994000	6.045187000	-1.420132000
1	-3.367284000	7.308593000	0.094624000
1	-4.745863000	6.109209000	1.765046000
16	-2.865364000	1.733938000	0.285537000
16	-0.803967000	0.815305000	-1.882111000
34	-1.490694000	-1.035863000	-0.839977000
6	-3.155568000	-1.248307000	-1.881865000
1	-3.490763000	-0.426502000	-2.507673000
6	-3.850264000	-2.386270000	-1.813162000
1	-4.760528000	-2.547099000	-2.386175000
16	-3.394026000	-3.674263000	-0.666312000
6	-3.256939000	-5.081467000	-1.747130000
1	-2.858730000	-4.865754000	-2.735018000
6	-3.542553000	-6.323880000	-1.355180000
1	-3.939439000	-6.543287000	-0.368665000
1	-3.367321000	-7.164036000	-2.019686000

Temperature 298.150 Kelvin. Pressure 1.00000 Atm.
Zero-point correction= 0.343231 (Hartree/Particle)
Thermal correction to Energy= 0.376078
Thermal correction to Enthalpy= 0.377022
Thermal correction to Gibbs Free Energy= 0.272487
Sum of electronic and zero-point Energies= -8146.182441
Sum of electronic and thermal Energies= -8146.149595
Sum of electronic and thermal Enthalpies= -8146.148651
Sum of electronic and thermal Free Energies= -8146.253186

Table S22. Structure 6a–6b

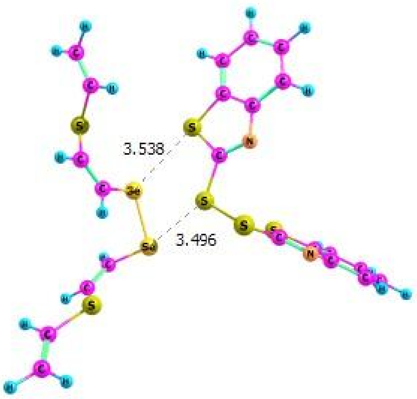

6	5.694568000	-2.285358000	-1.860873000
6	4.856187000	-1.974527000	-0.788956000
6	5.236435000	-2.246671000	0.547321000
6	6.477583000	-2.841899000	0.808762000
6	7.311283000	-3.152741000	-0.259221000
6	6.924449000	-2.877141000	-1.581109000
6	3.262776000	-1.358436000	0.963140000
7	4.306355000	-1.879615000	1.510141000
1	5.400317000	-2.074281000	-2.883780000
1	6.761980000	-3.047854000	1.835055000
1	8.275411000	-3.614678000	-0.070210000
1	7.592180000	-3.128059000	-2.399604000
16	3.260066000	-1.236155000	-0.812831000
16	1.918363000	-0.847069000	2.012163000
34	-1.728390000	-1.940881000	-1.173519000
6	-3.420660000	-2.725182000	-1.610821000
1	-3.805679000	-2.557200000	-2.610215000
6	-4.078246000	-3.501052000	-0.739377000
1	-5.004023000	-3.991530000	-1.033037000
16	-3.542188000	-3.832645000	0.912506000
6	-4.943321000	-4.731055000	1.535152000
1	-5.901144000	-4.475536000	1.087936000
6	-4.852686000	-5.600722000	2.541569000
1	-3.903520000	-5.862542000	2.998975000
1	-5.744926000	-6.068461000	2.944628000
6	1.195686000	5.382921000	-0.442263000
6	1.210649000	4.040770000	-0.059340000
6	2.284403000	3.497469000	0.683530000
6	3.360387000	4.316007000	1.051328000
6	3.344683000	5.653417000	0.672513000
6	2.273500000	6.182042000	-0.066694000
6	1.074708000	1.661334000	0.525153000
7	2.170188000	2.146940000	0.990781000
1	0.370786000	5.794936000	-1.014341000
1	4.180226000	3.891806000	1.620863000
1	4.171719000	6.299328000	0.950609000
1	2.283338000	7.229650000	-0.351731000
16	0.019089000	2.784489000	-0.374967000
16	0.531102000	-0.038048000	0.695835000
34	-2.358039000	0.322728000	-1.271865000
6	-3.270515000	0.403577000	0.410015000
1	-3.154305000	-0.474187000	1.037323000
6	-3.995760000	1.461922000	0.780522000
1	-4.485848000	1.492908000	1.750015000
16	-4.301222000	2.820834000	-0.328891000
6	-3.689587000	4.195059000	0.622385000
1	-2.789486000	3.992455000	1.196827000
6	-4.237879000	5.410134000	0.570515000
1	-5.138054000	5.615348000	-0.000864000
1	-3.781411000	6.242161000	1.097605000

Temperature 298.150 Kelvin. Pressure 1.00000 Atm.

Zero-point correction= 0.342345 (Hartree/Particle)
Thermal correction to Energy= 0.377861
Thermal correction to Enthalpy= 0.378805
Thermal correction to Gibbs Free Energy= 0.261014
Sum of electronic and zero-point Energies= -8146.209148
Sum of electronic and thermal Energies= -8146.173632
Sum of electronic and thermal Enthalpies= -8146.172688
Sum of electronic and thermal Free Energies= -8146.290479

Table S23. Structure A+

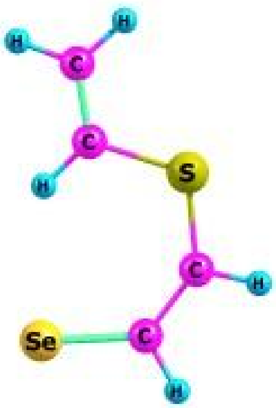

34	1.734659000	0.483697000	0.000077000
6	1.048228000	-1.166697000	-0.000221000
1	1.752038000	-1.995820000	-0.000463000
6	-0.311977000	-1.596791000	-0.000017000
1	-0.455056000	-2.677849000	0.000047000
16	-1.818010000	-0.893399000	0.000094000
6	-1.746345000	0.859506000	-0.000418000
1	-0.776323000	1.335106000	-0.001125000
6	-2.921868000	1.504645000	0.000117000
1	-3.880021000	0.994508000	0.000950000
1	-2.939117000	2.588757000	-0.000296000

Temperature 298.150 Kelvin. Pressure 1.00000 Atm.
Zero-point correction= 0.076341 (Hartree/Particle)
Thermal correction to Energy= 0.083656
Thermal correction to Enthalpy= 0.084600
Thermal correction to Gibbs Free Energy= 0.042557
Sum of electronic and zero-point Energies= -2954.809844
Sum of electronic and thermal Energies= -2954.802529
Sum of electronic and thermal Enthalpies= -2954.801585
Sum of electronic and thermal Free Energies= -2954.843628

Table S24. Structure TS (A$^+$ $\rightarrow$ B$^+$)

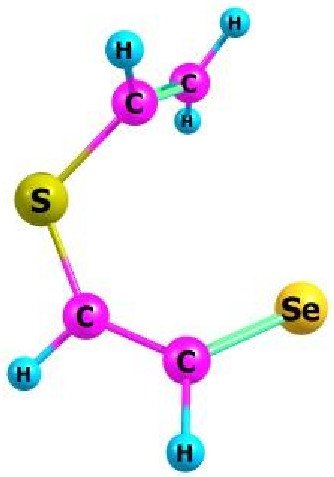

34	1.639926000	0.272384000	-0.036738000
6	0.746910000	-1.232830000	0.280300000
1	1.285222000	-2.078184000	0.709820000
6	-0.587491000	-1.547304000	-0.120550000
1	-0.761808000	-2.578821000	-0.436674000
16	-2.024617000	-0.710192000	-0.144751000
6	-1.796218000	0.936099000	0.532965000
1	-2.072459000	0.990805000	1.580993000
6	-1.526524000	1.988954000	-0.228781000
1	-1.309645000	1.919612000	-1.287822000
1	-1.524990000	2.979091000	0.215195000

Temperature 298.150 Kelvin. Pressure 1.00000 Atm.
Zero-point correction= 0.075482 (Hartree/Particle)
Thermal correction to Energy= 0.082148
Thermal correction to Enthalpy= 0.083092
Thermal correction to Gibbs Free Energy= 0.042603
Sum of electronic and zero-point Energies= -2954.800754
Sum of electronic and thermal Energies= -2954.794088
Sum of electronic and thermal Enthalpies= -2954.793144
Sum of electronic and thermal Free Energies= -2954.833634

Table S25. Structure B$^+$

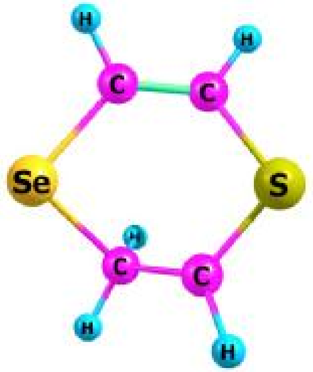

34	1.348694000	0.022678000	-0.164041000
6	0.173522000	1.439534000	0.195700000
1	0.641599000	2.418624000	0.230981000
6	-1.165913000	1.333152000	0.345191000
1	-1.788520000	2.188658000	0.578385000
16	-2.076748000	-0.043697000	-0.226888000
6	-1.012426000	-1.315457000	-0.137140000
1	-1.295274000	-2.182990000	-0.727252000
6	0.188114000	-1.327230000	0.712728000
1	0.006788000	-0.994324000	1.735683000
1	0.707991000	-2.281869000	0.690916000

Temperature 298.150 Kelvin. Pressure 1.00000 Atm.
Zero-point correction= 0.078375 (Hartree/Particle)
Thermal correction to Energy= 0.084420
Thermal correction to Enthalpy= 0.085364
Thermal correction to Gibbs Free Energy= 0.047337
Sum of electronic and zero-point Energies= -2954.846935
Sum of electronic and thermal Energies= -2954.840890
Sum of electronic and thermal Enthalpies= -2954.839946
Sum of electronic and thermal Free Energies= -2954.877973
